# A Four-Coordinate End-On Superoxocopper(II) Complex: Probing the Link between Coordination Number and Reactivity

**DOI:** 10.1021/jacs.3c12268

**Published:** 2024-08-14

**Authors:** Suman Debnath, Shoba Laxmi, Olivia McCubbin Stepanic, Sebastian Y. Quek, Maurice van Gastel, Serena DeBeer, Tobias Krämer, Jason England

**Affiliations:** † Division of Chemistry and Biological Chemistry, School of Chemistry, Chemical Engineering and Biotechnology, Nanyang Technological University, 21 Nanyang Link, 637371 Singapore; ‡ Max Planck Institute for Chemical Energy Conversion, Stiftstr. 34–36, Mülheim an der Ruhr D-45470, Germany; § Max-Planck-Institut für Kohlenforschung, Kaiser-Wilhelm-Platz, Mülheim an der Ruhr D-45470, Germany; ∥ Department of Chemistry, Maynooth University, Maynooth W23 F2H6, Co. Kildare, Ireland; ⊥ Hamilton Institute, Maynooth University, Maynooth W23 F2H6, Co. Kildare, Ireland; # School of Chemistry, University of Lincoln, Lincoln LN6 7TW, U.K.

## Abstract

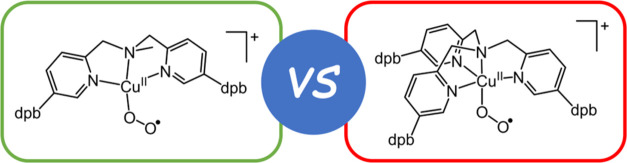

Although the reactivity of five-coordinate end-on superoxocopper(II) complexes, Cu^II^(η^1^-O_2_^•–^), is dominated by hydrogen atom transfer, the majority of four-coordinate Cu^II^(η^1^-O_2_^•–^) complexes published thus far display nucleophilic reactivity. To investigate the origin of this difference, we have developed a four-coordinate end-on superoxocopper(II) complex supported by a sterically encumbered bis(2-pyridylmethyl)amine ligand, dpb_2_-^Me^BPA (**1**), and compared its substrate reactivity with that of a five-coordinate end-on superoxocopper(II) complex ligated by a similarly substituted tris(2-pyridylmethyl)amine, dpb_3_-TMPA (**2**). Kinetic isotope effect (KIE) measurements and correlation of second-order rate constants (*k*_2_’s) versus oxidation potentials (*E*_ox_) for a range of phenols indicates that the complex [Cu^II^(η^1^-O_2_^•–^)(**1**)]^+^ reacts with phenols via a similar hydrogen atom transfer (HAT) mechanism to [Cu^II^(η^1^-O_2_^•–^)(**2**)]^+^. However, [Cu^II^(η^1^-O_2_^•–^)(**1**)]^+^ performs HAT much more quickly, with its *k*_2_ for reaction with 2,6-di-*tert*-butyl-4-methoxyphenol (MeO-ArOH) being >100 times greater. Furthermore, [Cu^II^(η^1^-O_2_^•–^)(**1**)]^+^ can oxidize C–H bond substrates possessing stronger bonds than [Cu^II^(η^1^-O_2_^•–^)(**2**)]^+^ is able to, and it reacts with *N*-methyl-9,10-dihydroacridine (^Me^AcrH_2_) approximately 200 times faster. The much greater facility for substrate oxidation displayed by [Cu^II^(η^1^-O_2_^•–^)(**1**)]^+^ is attributed to it possessing higher inherent electrophilicity than [Cu^II^(η^1^-O_2_^•–^)(**2**)]^+^, which is a direct consequence of its lower coordination number. These observations are of relevance to enzymes in which four-coordinate end-on superoxocopper(II) intermediates, rather than their five-coordinate congeners, are routinely invoked as the active oxidants responsible for substrate oxidation.

## Introduction

Mononuclear end-on superoxocopper(II) species, Cu^II^(η^1^-O_2_^•–^), have been invoked as hydrogen atom abstracting agents in a plethora of O_2_ activating copper enzymes.^1,2^ This includes the noncoupled binuclear copper enzymes PHM, DβM, and TβM, which catalyze hydroxylation of activated C–H bonds of their native substrates,^3^ and formylglycine-generating enzyme (FGE), which is responsible for the conversion of cysteine to formylglycine.^4^ Although the O_2_ activating sites in these two sets of enzymes are distinct, they are both widely believed to form four-coordinate Cu^II^(η^1^-O_2_^•–^) active oxidants.

In the case of FGE, the supporting ligands are three cysteinate donors, one of which is the substrate and the other two are active site residues.^5−8^ In contrast, the noncoupled binuclear copper monooxygenases possess active sites containing two separate Cu binding sites that are separated by about 11 Å.^9^ Both contain a single Cu ion coordinated by three amino acid residues and, according to the canonical mechanism, one (Cu_M_) is thought to be responsible for O_2_ activation and substrate oxidation, while the other (Cu_H_) acts solely as an electron transfer site.^10,11^ More recent studies have suggested that, instead, the Cu^II^(η^1^-O_2_^•–^) intermediate (at Cu_M_) reacts with the cosubstrate ascorbate to yield a hydroperoxocopper(II) species, which converts to a closed conformation containing the true C–H bond hydroxylating oxidant, believed to be a Cu^II^(μ–OH)(μ-O^•^)Cu^II^ species.^12−15^ Strong support for the intermediacy of a four-coordinate Cu^II^(η^1^-O_2_^•–^) species in this family of enzymes was provided by measurement of an (albeit photoreduced) X-ray crystal structure of an end-on O_2_ adduct in the Cu_M_ site of PHM.^16^ Incidentally, a recent neutron crystal structure of a lytic polysaccharide monooxygenase (LPMO), which catalyzes oxidative breakdown of recalcitrant polysaccharides, revealed a five-coordinate Cu^II^(η^1^-O_2_^•–^).^17^ However, contemporary studies concluded that LPMOs are, in fact, peroxygenases, rather than oxygenases, and the Cu^II^(η^1^-O_2_^•–^) intermediates observed in these enzymes are most likely formed during off-pathway reduction of O_2_ to H_2_O_2_ (i.e., they do not react directly with substrate).^18−24^

**Chart 1:**
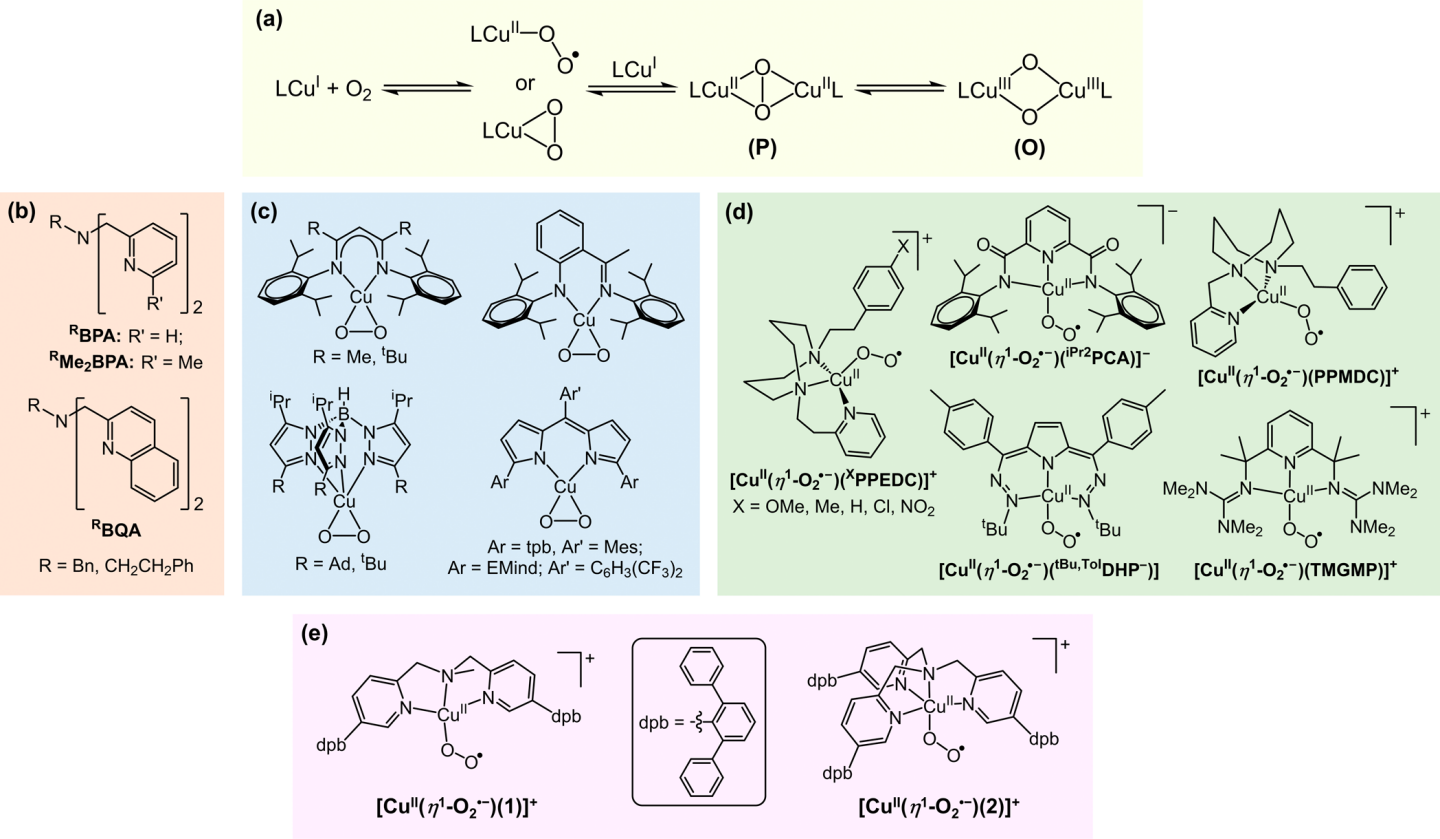
Selected Copper(I) + O_2_ Reactivity, Ligands, and Cu-O_2_ Adducts Relevant to this Worka

The vast majority of synthetic Cu^II^(η^1^-O_2_^•–^) model complexes published, thus far, are supported by tetradentate ligands and possess five-coordinate geometries.^25−27^ This leaves the copper centers coordinatively saturated and restricts the O_2_-derived ligand to end-on binding. Ligands of lower denticity usually yield μ-η^2^:η^2^-peroxodicopper(II), Cu^II^(μ-η^2^:η^2^-O_2_^2–^)Cu^II^ (**P**), and/or bis(μ-oxo)dicopper(III), Cu^III^(μ-O^2–^)_2_Cu^III^ (**O**), complexes (Chart 1a). These two species are in equilibrium with one another, and small changes in the supporting ligand can lead to a shift in the predominance of one over the other. This is exemplified by the tridentate *N*,*N*-bis(2-pyridylmethyl)amine (BPA) ligand framework, where the substitution patterns of the pyridine and/or the central tertiary amine donors (Chart 1b) can shift the equilibrium entirely from one species to the other.^28^ More specifically, introduction of steric bulk onto the BPA ligand framework can shift the equilibrium from exclusive formation of **O** (i.e., ^R^BPA, with R′ = H)^29−31^ to mixtures **O** and **P** (^Bn^BQA)^32^ and, in the bulkiest systems, to formation of only **P** (^R^Me_2_BPA and ^Phe^BQA).^32,33^ These observations have been attributed to steric inhibition of the close approach of the Cu ions, which is required for formation of the **O** core. Thus far, no mononuclear O_2_ adducts have been observed in these BPA complexes. However, kinetic data suggests that **O** and **P** are formed by preequilibrium binding of O_2_ to a copper(I) BPA complex, to give either a Cu^II^(η^1^-O_2_^•–^) or Cu^II^(η^2^-O_2_^•–^) complex, followed by rate-determining reaction with a second copper(I) center.^29,32,34^

a (a) General scheme for reaction of low-coordination number copper(I) complexes with O_2_ and (b) published BPA-derived ligands that support this chemistry. Previously reported examples of low-coordinate (c) side-on (η^2^) and (d) end-on (η^1^) 1:1 adducts of copper complex and O_2_. (e) The Cu^II^(η^1^-O_2_^•–^) complexes studied in this work.

Inclusion of bulky substituents onto bidentate and tridentate ligands can be used to inhibit the formation of dicopper-O_2_ adducts.^35−40^ However, in this scenario, side-on coordination to yield either η^2^-superoxocopper(II) or, in the case of more strongly reducing ligands, η^2^-peroxocopper(III) complexes is commonly observed (Chart 1c).^41^ This binding mode retards reactivity, which has allowed several of these complexes to be crystallographically characterized.^35,38−40,42^ All exceptions to this side-on O_2_ binding preference reported, thus far, were obtained using linear tridentate ligands (Chart 1d).^43−47^ Interestingly, the majority of these four-coordinate Cu^II^(η^1^-O_2_^•–^) complexes display nucleophilic reactivity. This is personified by Tolman’s complex [Cu^II^(η^1^-O_2_^•–^)(^iPr2^PCA)]^−^, which reacts with phenols primarily via proton transfer and subsequent electron transfer (i.e., PT/ET),^48^ and performs deformylation of a selection of electron-rich aldehydes.^49,50^ In contrast, Itoh’s and Anderson’s respective four-coordinate complexes [Cu^II^(η^1^-O_2_^•–^)(^X^PPEDC)]^+^ and [Cu^II^(η^1^-O_2_^•–^)(^*t*Bu,Tol^DHP^–^)] display predominant electrophilic character.^51^ Whereas [Cu^II^(η^1^-O_2_^•–^)(^*t*Bu,Tol^DHP^–^)] is a weak oxidant that shows no reactivity with C–H bonds,^47^ [Cu^II^(η^1^-O_2_^•–^)(^X^PPEDC)]^+^ can directly oxidize ferrocenes and intramolecularly hydroxylate an *N*-ethylphenyl substituent of the ligand.^44,52,53^ The latter is the only published example of C–H bond oxidation by a four-coordinate Cu^II^(η^1^-O_2_^•–^) complex and remains the only example of C–H bond hydroxylation by any synthetic Cu^II^(η^1^-O_2_^•–^) species.

The predominance of nucleophilic substrate reactivity in four-coordinate Cu^II^(η^1^-O_2_^•–^) complexes is in stark contrast to five-coordinate and enzymatic Cu^II^(η^1^-O_2_^•–^) species, whose substrate reactivity is characterized by electrophilic hydrogen atom abstractions.^25,54,55^ The origin of this difference has not been investigated but likely derives, in part, from the divergence in the nature/basicity of the donors in the supporting ligands. In the hope of gaining insight into the aforementioned disparity in reactivity, we sought a pair of four- and five-coordinate Cu^II^(η^1^-O_2_^•–^) complexes supported by tridentate and tetradentate ligands, respectively, that differ only in the presence/absence of a single donor. Given our success in stabilizing five-coordinate Cu^II^(η^1^-O_2_^•–^) complexes of tetradentate tris(2-pyridylmethyl)amine (TMPA) ligands by incorporating large aryl substituents onto the 5-position of their pyridine donors,^56^ we developed a similarly substituted tridentate bis(2-pyridylmethyl)amine (BPA). The resulting ligand dpb_2_-^Me^BPA (**1**) was found to support a Cu^II^(η^1^-O_2_^•–^) complex, and its reactivity was compared to that of dpb_3_-TMPA (**2**), which deviates from the former by replacement of one 2-pyridylmethyl arm by a methyl substituent (Chart 1e).

## Results and Discussion

**Figure 1:**
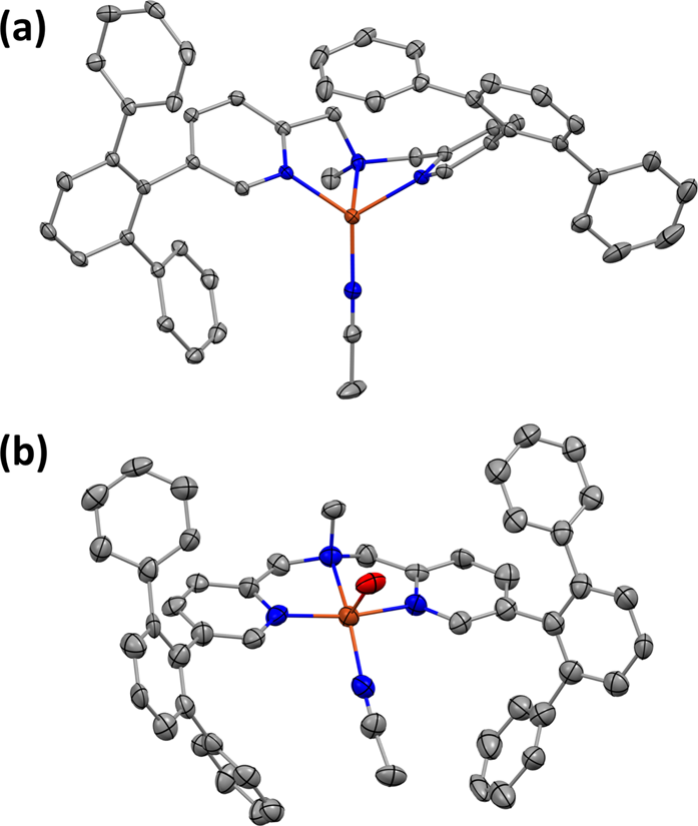
X-ray crystal structures of (a) [Cu^I^(**1**)(NCMe)](SbF_6_) and (b) [Cu^II^(**1**)(NCMe)(OH_2_)](ClO_4_)_2_, depicted using 50% thermal ellipsoids. For clarity, hydrogen atoms, counterions, and solvent molecules have been omitted. Gray, orange, blue, and red spheroids correspond to carbon, copper, nitrogen, and oxygen atoms, respectively.

Copper(I) complexes of formulation [Cu^I^(**1**)(NCMe)](X), where X = B(C_6_F_5_)_4_^–^ and SbF_6_^–^, were prepared by a combination of **1** with the corresponding [Cu^I^(NCMe)_4_](X) salts. Oxygenation studies were performed exclusively using [Cu^I^(**1**)(NCMe)][B(C_6_F_5_)_4_], but crystals suitable for X-ray crystallographic characterization could only be grown for [Cu^I^(**1**)(NCMe)](SbF_6_) (Figures 1a and S10, Table S2). The latter possesses a distorted trigonal pyramidal geometry (as indicated by the geometry index τ_4_ = 0.81),^57^ with the tertiary amine donor in a pseudoaxial position and Cu–N_amine_, average Cu–N_pyridine_, and Cu–N_MeCN_ bond distances of 2.206(2), 2.035(2), and 1.891(2) Å, respectively. This is typical for four-coordinate copper(I) complexes of BPA-type ligands that include acetonitrile (MeCN) coligands.^30,32,33,58−60^

In contrast, recrystallization of the product obtained from reaction of **1** with the copper(II) salt Cu^II^(ClO_4_)_2_·6H_2_O afforded the square-based pyramidal complex (τ_5_ = 0.05)^61^ [Cu^II^(**1**)(NCMe)(OH_2_)](ClO_4_)_2_ (Figures 1b and S11, Table S3). Therein, N-donors occupy the equatorial positions, and a H_2_O ligand coordinates in the axial site. As expected for a Jahn–Teller distorted copper(II) complex, the Cu–O distance is comparatively long, at 2.273(13) Å. Consistent with its higher oxidation state, the Cu–N_amine_ and average Cu–N_pyridine_ bond lengths in this complex, 2.016(13) and 1.958(6) Å, respectively, are significantly contracted relative to those of [Cu^I^(**1**)(NCMe)](SbF_6_). The shortening of the former distance (by approximately 0.19 Å) is particularly large and is likely, partly, due to the accompanying elongation of the Cu–N_MeCN_ distance (by approximately 0.12 Å) to 2.015(13) Å. This increase in Cu–N_MeCN_ bond length can be attributed to reduced π back-bonding in copper(II), relative to copper(I).

Cyclic voltammetry measurements for copper(II) complex [Cu^II^(**1**)(NCMe)(OH_2_)](ClO_4_)_2_ yielded a quasi-reversible Cu^II^/Cu^I^ redox couple in acetonitrile solution (Figure S12), with an *E*_1/2_ value of −0.30 V (vs Fc^+^/Fc^0^). This is more than 100 mV positive of the *E*_1/2_ recorded for [Cu^II^(**2**)(NCMe)]^2+^ (−0.41 V), but it is similar to values reported for [Cu^II^(dtbpb_3_-TMPA)(NCMe)]^2+^ and [Cu^II^(^Me^BPA)(NCMe)(OTf)]^+^ (−0.32 and −0.29 V, respectively).^56,62^ From this, we can expect [Cu^I^(**1**)(NCMe)]^+^ to have a similar affinity for O_2_ binding as [Cu^I^(dtbpb_3_-TMPA)]^+^, which displays saturation of O_2_ binding in THF solution at ≤−70 °C.

### Superoxocopper(II) Complex Formation and Characterization

**Figure 2:**
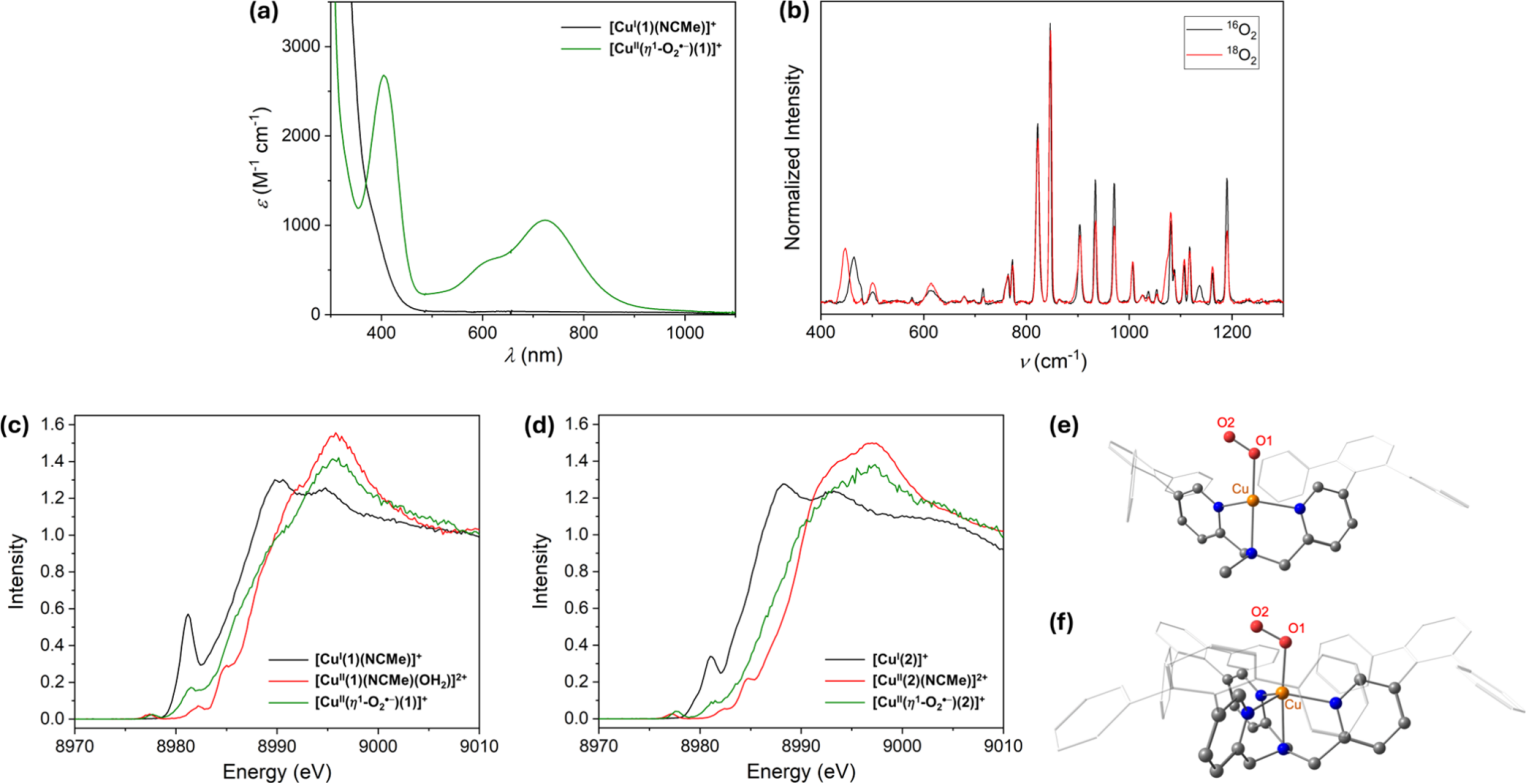
(a) UV–vis spectra of [Cu^I^(**1**)(NCMe)]^+^ and [Cu^II^(η^1^-O_2_^•–^)(**1**)]^+^ (black and green lines, respectively), recorded in THF solution at −90 °C. (b) Resonance Raman spectra of [Cu^II^(η^1^-^16^O_2_^•–^)(**1**)]^+^, prepared using natural abundance O_2_, and [Cu^II^(η^1^-^18^O_2_^•–^)(**1**)]^+^ (black and red lines, respectively), recorded in frozen *d*_8_-THF solution using λ_ex_ = 407 nm. K-edge HERFD XAS data recorded for (c) [Cu^I^(**1**)(NCMe)]^+^, [Cu^II^(**1**)(NCMe)(OH_2_)]^2+^, and [Cu^II^(η^1^-O_2_^•–^)(**1**)]^+^ (black, red, and green lines, respectively) and (d) [Cu^I^(**2**)]^+^, [Cu^II^(**2**)(NCMe)]^2+^, and [Cu^II^(η^1^-O_2_^•–^)(**2**)]^+^ (black, red, and green lines, respectively). DFT (B3LYP-D3(BJ)/def2-TZVP) geometry-optimized structures of (e) [Cu^II^(η^1^-O_2_^•–^)(**1**)]^+^ and (f) [Cu^II^(η^1^-O_2_^•–^)(**2**)]^+^. Hydrogen atoms have been omitted for clarity.

Bubbling O_2_ through pale yellow tetrahydrofuran solutions of [Cu^I^(**1**)(NCMe)][B(C_6_F_5_)_4_], at −90 °C (Figure 2a), led to formation of a green species displaying ultraviolet–visible (UV–vis) spectral features (λ_max_ = 404 and 722 nm; ε_max_ = 2680 and 1060 M^–1^ cm^–1^, respectively) characteristic of a Cu^II^(η^1^-O_2_^•–^) complex.^26,27^ For comparison, some published examples are listed in Table 1. In contrast to the time frame of <10 s (s) required for generation of [Cu^II^(η^1^-O_2_^•–^)(**2**)]^+^, maximum formation of [Cu^II^(η^1^-O_2_^•–^)(**1**)]^+^ took >180 s (Figure S14). This difference can be attributed to a divergence in mechanism, with the former involving oxygenation of a copper(I) complex possessing a vacant coordination site (i.e., [Cu^I^(**2**)]^+^, eq 2) and the latter requiring dissociation of a solvent ligand prior to reaction with O_2_ (eqs 1 and 2). Consistent with the need for dissociation of acetonitrile from [Cu^I^(**1**)(NCMe)]^+^ prior to oxygenation, the addition of small amounts of acetonitrile (1–5 equiv) results in significantly decreased yields of [Cu^II^(η^1^-O_2_^•–^)(**1**)]^+^ (Figure S15). Crucially, the observed Cu^II^(η^1^-O_2_^•–^) complex, [Cu^II^(η^1^-O_2_^•–^)(**1**)]^+^, is stable under the conditions of the experiment, with no evidence for conversion to higher nuclearity **P** or **O** species. Furthermore, warming solutions of [Cu^II^(η^1^-O_2_^•–^)(**1**)]^+^ led to self-decay, but without formation of chromophores characteristic of **P** or **O**.

**Table 1:** Summary of Spectroscopic Parameters for the End-On Superoxocopper(II) Complexes, [Cu^II^(η^1^-O_2_^•–^)(L)]*^n^*, Described Herein and Selected Published Examples

	L	λ_max_, nm (ε_max_, M^–1^ cm^–1^)	ν(O–O) (cm^–1^)	ν(Cu–O) (cm^–1^)
4-coordinate	**1**	404 (2680), 722 (1060)	1137	464
	^H^PPEDC^44^	397 (4200), 570 (850), 705 (1150)	1033	457
	PPMDC^45^	386 (4430), 558 (820), 725 (1160)	1064	464
	TMGMP^46^	359 (1800), 595 (970)	1136	
	^*t*Bu,Tol^DHP^–^^47^	420 (13,600), 670	1067	
	^iPr2^PCA^2–^^49^	628 (1400)	1102	
5-coordinate	**2**(56)	430 (3950), 584 (1150), 760 (1690)	1126	467
	^1PV^TMPA^63^	410 (3700), 585 (900), 741 (1150)	1130	482
	TMG_3_tren^64−66^	448 (3400), 676, 795	1120	435
	^TMG^N_3_S^67^	442 (3700), 642 (1525), 742 (1675)	1105	446


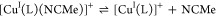
1
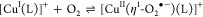
2

Confirmation of the formation of [Cu^II^(η^1^-O_2_^•–^)(**1**)]^+^ was obtained via resonance Raman spectroscopy (Figures 2b and S13). Using natural abundance O_2_ and an excitation wavelength (λ_ex_) of 407 nm, peaks at 1137 and 464 cm^–1^ were observed. Production of samples using ^18^O_2_ gas caused these peaks to shift by 63 and 17 cm^–1^, respectively, to 1074 and 447 cm^–1^. Based upon the energies of these peaks and the magnitudes of their isotope shifts, which are very similar to those of previously reported Cu^II^(η^1^-O_2_^•–^) complexes (see Table 1 for examples),^26,27^ they can be assigned as O–O and Cu–O stretches, respectively. These ν(O–O) and ν(Cu–O) values are distinct from those of their side-on bound congeners (i.e., Cu(η^2^-O_2_) complexes), which tend to be in the regions of ∼1000 cm^–1^ and 490–560 cm^–1^, respectively.^26^

The electronic structure of [Cu^II^(η^1^-O_2_^•–^)(**1**)]^+^ was probed by Cu K-edge high energy resolution fluorescence detection X-ray absorption spectroscopy (HERFD XAS) measurements. To provide a frame of reference, data were also collected for [Cu^II^(η^1^-O_2_^•–^)(**2**)]^+^, the copper(I) starting complexes [Cu^I^(**1**)(NCMe)]^+^ and [Cu^I^(**2**)]^+^, and the copper(II) salts [Cu^II^(**1**)(NCMe)(OH_2_)]^2+^ and [Cu^II^(**2**)(NCMe)]^2+^. The spectral features of the series of compounds supported by compounds **1** and **2**, respectively, are broadly similar (Figure 2c,d). Both Cu^I^ complexes display an intense feature in the rising edge at ∼8981 eV, which is characteristic of Cu^I^ and arises from 1s → 4p transitions. Although this appears to be a single and higher intensity feature in [Cu^I^(**1**)(NCMe)]^+^, which indicates that all 1s → 4p transitions in this complex occur at similar energies, the corresponding feature in [Cu^I^(**2**)]^+^ possesses a shoulder at ∼8979 eV. This difference can be attributed to the geometry of [Cu^I^(**2**)]^+^ being closer to a true trigonal pyramid than that of [Cu^I^(**1**)(NCMe)]^+^. The geometry of the latter is significantly distorted toward tetrahedral, which will manifest as a reduced energetic separation of the 4p orbitals. Additionally, a trend in the energy of the rising edge can be seen within both the **1** and **2** ligated series of complexes, with Cu^II^ salts > Cu^II^(η^1^-O_2_^•–^) > Cu^I^ complexes. This is concordant with expectations, as increasing the oxidation state leads to a contraction of core orbitals, thereby increasing the energy of the rising edge. The high metal–ligand covalency of the Cu^II^(η^1^-O_2_^•–^) moiety ameliorates this effect, leading to an intermediate rising edge energy.

Pre-edge and rising edge features attributable to ligand-to-metal charge transfers (LMCTs) are seen in the XAS spectra of the Cu^II^ salts, [Cu^II^(**1**)(NCMe)(OH_2_)]^2+^ and [Cu^II^(**2**)(NCMe)]^2+^. Although similar, albeit weaker, features are seen for the Cu^II^(η^1^-O_2_^•–^) complexes, they likely arise from residual Cu^I^ starting materials. More specifically, the XAS spectra of [Cu^II^(η^1^-O_2_^•–^)(**1**)]^+^ and [Cu^II^(η^1^-O_2_^•–^)(**2**)]^+^, depicted in Figure 2, are estimated to contain contributions from approximately 20% [Cu^I^(**1**)(NCMe)]^+^ and 5% [Cu^I^(**2**)]^+^, respectively. These impurities prevent any definitive statements regarding the greater intensity observed for the pre-edge of [Cu^II^(η^1^-O_2_^•–^)(**2**)]^+^ relative to that of [Cu^II^(η^1^-O_2_^•–^)(**1**)]^+^. However, we can confidently conclude that the pre-edge intensities and energies of the Cu^II^(η^1^-O_2_^•–^) complexes and Cu^II^ salts are markedly similar to one another. Additionally, the positions of the pre-edges of [Cu^II^(η^1^-O_2_^•–^)(**1**)]^+^ and [Cu^II^(η^1^-O_2_^•–^)(**2**)]^+^ are both shifted by +0.4 eV relative to those of the corresponding Cu^II^ salts.

Density functional theory (DFT) calculations support the formulation of the title Cu–O_2_ adduct as [Cu^II^(η^1^-O_2_^•–^)(**1**)]^+^. Consistent with previous reports,^43,52,63,64,67,68^ geometry optimizations revealed that the triplet (*S* = 1) state was most stable for end-on binding of the superoxo moiety (Figure S33 and Table S13). The geometry-optimized structure of the lowest energy triplet state calculated for [Cu^II^(η^1^-O_2_^•–^)(**1**)]^+^ (Figure 2e) displays a seesaw geometry (τ_4_ = 0.42). This contrasts with the distorted trigonal pyramidal geometries of the experimentally observed and calculated structures (both τ_4_ = 0.81) of starting complex [Cu^I^(**1**)(NCMe)]^+^ (Figures 1a and S32), but is similar to the DFT geometry-optimized structures of other four-coordinate Cu^II^(η^1^-O_2_^•–^) complexes (Table 2). The lowest energy triplet state of [Cu^II^(η^1^-O_2_^•–^)(**1**)]^+^ displays a Cu–O–O bond angle and an O–O bond length of 118.8° and 1.253 Å, respectively (Table S13). The latter is short for a superoxo anion. However, the functional B3LYP has a well-documented tendency to overestimate metal–ligand bond lengths, which will reduce the extent of charge transfer into π-acceptor ligands (e.g., O_2_). Thus, an underestimation of the O–O bond length is within expectations.

**Table 2:** Summary of Key Bond Metric Data for DFT Geometry-Optimized Structures of the [Cu^II^(η^1^-O_2_^•–^)(L)]*^n^* Complexes Described Herein and Selected Published Examples

	L	Cu–O1 (Å)	O1–O2 (Å)	Cu–O1–O2 (deg)	τ_4_^57^	τ_5_^61^
4-coordinate	**1**^a^	1.984	1.253	118.8	0.42	
	^H^PPEDC^b^	1.907	1.259	121.0	0.37	
	PPMDC^c^	1.937	1.297	91.2	0.36	
	TMGMP^d^	1.936	1.300	113.8	0.16	
	^*t*Bu,Tol^DHP^–^e	2.097	1.272	112.5	0.56	
	^iPr2^PCA^2–^f	1.966	1.292	115.1	0.16	
5-coordinate	**2**^a^	1.968	1.264	113.8		0.96
	TMPA^g^	1.976	1.285	116.5		0.92
	TMG_3_tren^h^	1.988	1.292	120.1		0.92
	^TMG^N_3_S^h^	1.994	1.286	113.0		0.89

a B3LYP-D3/def2-TZVP, this work.

b M11-L/SDD/6-31G(d), ref (52).

c B97x/SDD/D95, ref (45).

d TPSSh-D3/def2-TZVP, ref (46).

e M06L/def2-TZVPP/def2-TZVP, ref (47).

f mPW/SDD/6-311+G(d,p)/6-31+G(d,p), ref (43).

g ZORA-BP86-D3(BJ)/ZORA-def2-TZVP(-f)/ZORA-def2-SVP, ref (56).

h B3LYP-D3(BJ)/def2-TZVP, ref (67).

As might be expected, time-dependent DFT (TDDFT) calculated UV–vis spectra for the triplet and open-shell singlet states are, in terms of general appearance, very similar to one another (Figures S42–S44). Although the experimental spectral features are reasonably well reproduced for both spin states, the relative band intensities in the triplet state better fit experiment. Similarly, due to deviation from the experimental spectrum, a five-coordinate acetonitrile-coordinated formulation [Cu^II^(η^1^-O_2_^•–^)(**1**)(NCMe)]^+^ can also be safely discarded (see the Supporting Information). TDDFT calculated XAS spectra yield similar conclusions (Figure S49 and the accompanying discussion). Those of the triplet and singlet states of [Cu^II^(η^1^-O_2_^•–^)(**1**)]^+^ have rising edges that closely resemble one another. However, the calculated XAS spectrum of the triplet state more accurately reproduces the experimentally observed shift in pre-edge energy relative to [Cu^II^(**1**)(NCMe)(OH_2_)]^2+^ (0.4 eV in both experiment and calculation) and the relative differences with respect to the rising edge energies of [Cu^I^(**1**)(NCMe)]^+^ and [Cu^II^(**1**)(NCMe)(OH_2_)]^2+^. The reliability of our computational models is validated by the TDDFT calculated XAS spectra of [Cu^I^(**1**)(NCMe)]^+^ and [Cu^II^(**1**)(NCMe)(OH_2_)]^2+^, which closely reproduce their experimentally observed features. See the Supporting Information for a discussion of these results.

To better describe the electronic structure of [Cu^II^(η^1^-O_2_^•–^)(**1**)]^+^ and more accurately estimate its singlet–triplet energy splitting, multiconfigurational N-electron valence state perturbation theory calculations, based on a (12,12) complete active space reference wave function (CASSCF/NEVPT2), were performed. Active space natural orbitals and their occupation numbers are provided in the Supporting Information (Figures S40 and S41). As expected, the CASSCF/NEVPT2 calculated triplet state is characterized by a single determinant, with singly occupied 3d_*z*^2^_ and π*_v_ orbitals and full occupation of the remaining four Cu 3d orbitals (Table S16). In contrast, the singlet wave function is composed of two configurations with weights of 0.63 and 0.34 (Table S17), which indicates that this state possesses high biradical character. These singly occupied orbitals have occupation numbers of 1.294 and 0.704 (Figure S41), respectively, and show more mixing between the Cu 3d_*z*^2^_ and the O 2p orbitals than the triplet state. The other Cu 3d orbitals are doubly occupied. In essence, the CASSCF calculations yield a Cu^II^(η^1^-O_2_^•–^) description displaying either a triplet or singlet diradical state. While DFT predicts that the triplet state is significantly more stable than the open-shell singlet state, CASSCF/NEVPT2 places the former 1.2 kcal mol^–1^ higher in energy than the latter (Tables S18 and S19). Thus, it can be concluded that the two spin states are nearly degenerate. This outcome is consistent with reports made for related systems.^69,70^

### Reaction with O–H Bond Substrates

**Figure 3:**
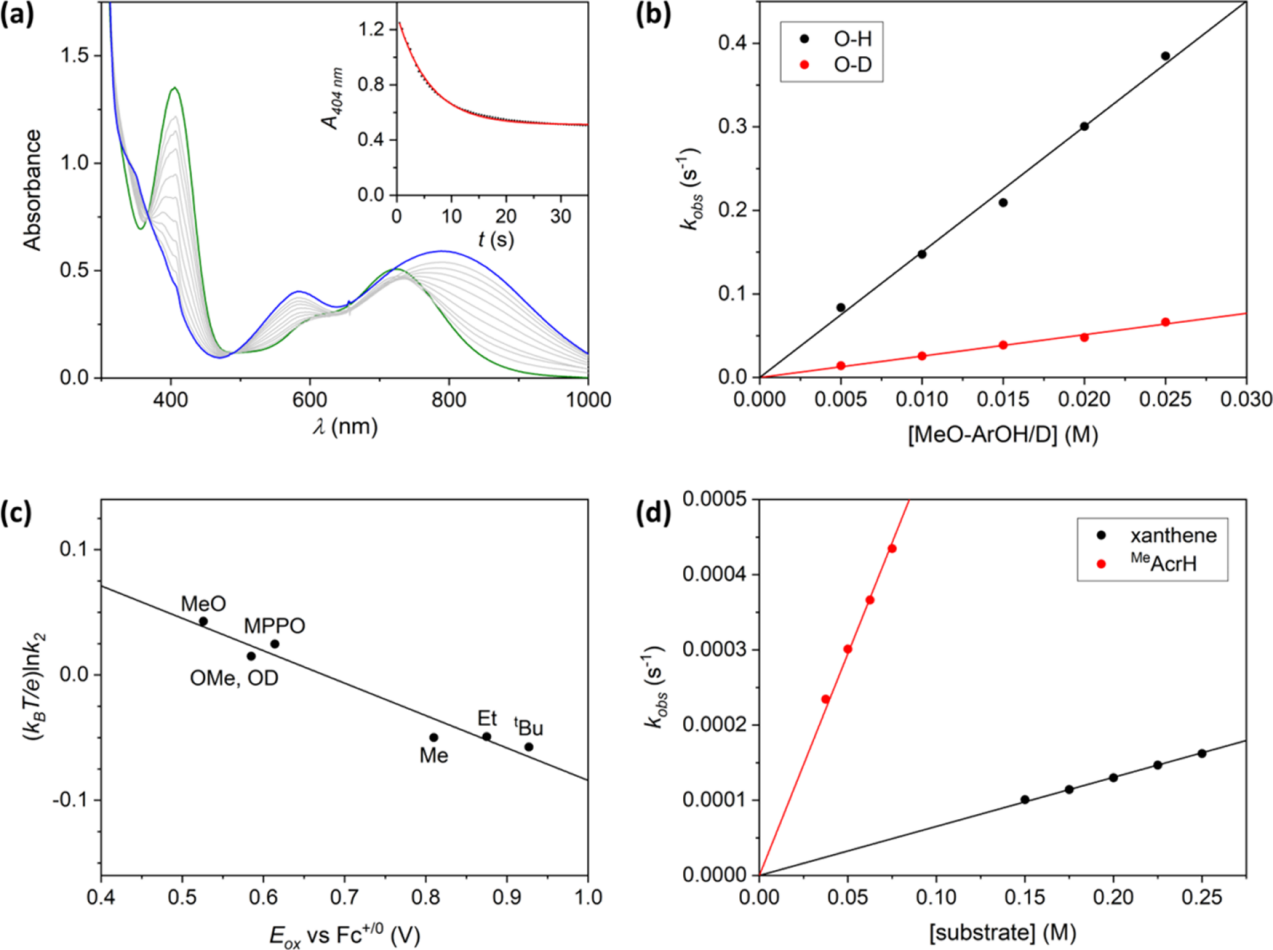
(a) Main: The UV–vis spectral changes observed upon reaction of [Cu^II^(η^1^-O_2_^•−)^(**1**)]^+^ with 2,6,-di-*tert*-butyl-4-methoxyphenol (MeO-ArOH). Green, black, and gray lines correspond to [Cu^II^(η^1^-O_2_^•–^)(**1**)]^+^, the product of reaction, and the spectra recorded in between, respectively. Inset: absorbance at 404 nm as a function of time and best-fit line (black dots and red line, respectively). (b) Plot of the observed rate constants (*k*_obs_, s^–1^) versus substrate concentration (M), including best-fit lines, recorded for reaction of [Cu^II^(η^1^-O_2_^•–^)(**1**)]^+^ with MeO-ArOH and MeO-ArOD (black and red circles/lines, respectively). (c) Marcus plot of (*k*_B_*T*/*e*) ln(*k*_2_) values obtained from reaction of [Cu^II^(η^1^-O_2_^•–^)(**1**)]^+^ with 4-substituted 2,6,-di-*tert*-butylphenols (X-ArOH) versus the oxidation potentials (*E*_ox_, V vs Fc^+^/Fc^0^) of the X-ArOH substrates. (d) Plot of the observed rate constants (*k*_obs_, s^–1^) versus substrate concentration (M) recorded for reaction of [Cu^II^(η^1^-O_2_^•–^)(**1**)]^+^ with *N*-methyl-9,10-dihydroacridine (^Me^AcrH_2_) and xanthene (black and red circles/lines, respectively). All reactions depicted were performed in THF solution at −90 °C.

To allow comparison with [Cu^II^(η^1^-O_2_^•–^)(**2**)]^+^, reaction of [Cu^II^(η^1^-O_2_^•–^)(**1**)]^+^ with a series of 4-substituted (X) 2,6-di-*tert*-butylphenols (X-ArOH) was studied at −90 °C. In all cases, this proceeded with first-order decay of the UV–vis features associated with the Cu^II^(η^1^-O_2_^•–^) unit and growth of two bands centered at around 530–590 and 730–790 nm (Figures 3a and S16), respectively. The precise spectral features (λ_max_ and ε_max_ values) of the products were found to depend upon the identity of substituent X. However, the intensities of the two bands suggest that they possess some ligand-to-metal charge transfer (LMCT) character. Additionally, electron paramagnetic resonance (EPR) spectra of the products of the reaction with X-ArOH (Figure S27) all display signals indicative of a copper(II) complex possessing a d_*x*^2^–*y*^2^_ ground state (i.e., *g*_*z*_ > *g*_*x*_ = *g*_*y*_). The para substituents (X) of the various X-ArOH substrates did not significantly impact the EPR spectral parameters (Table S5). This contrasts with observations made for the reaction of [Cu^II^(η^1^-O_2_^•–^)(**1**)]^+^ with TEMPO-H, which is complete within a few seconds and yields a product with UV–vis features typical of a hydroperoxocopper(II) complex (Figure S18; λ_max_ = 384 nm).^67,71−76^ Interestingly, this putative hydroperoxocopper(II) undergoes slow decay even at −90 °C, and efforts to generate it by treatment of copper(II) complex [Cu^II^(**1**)(NCMe)(OH_2_)](ClO_4_)_2_ with H_2_O_2_/triethylamine were not successful (i.e., it formed as a very short-lived species).

Taken together, the aforementioned findings are consistent with the formulation of the products of the reaction of [Cu^II^(η^1^-O_2_^•–^)(**1**)]^+^ with X-ArOH as [Cu^II^(OAr-X)(**1**)]^+^. This notion is supported by the striking similarity of their UV–vis spectra to those reported for the phenoxocopper(II) complexes [Cu^II^(OAr)(^iPr2^PCA)]^−^ and [Cu^II^(OAr)(^X^PPEDC)]^+^, which were obtained from reaction of the corresponding four-coordinate superoxocopper(II) complexes, [Cu^II^(η^1^-O_2_^•–^)(^iPr2^PCA)]^−^ and [Cu^II^(η^1^-O_2_^•–^)(^X^PPEDC)]^+^, with 4-substituted phenols.^48,53^ In particular, [Cu^II^(OAr)(^iPr2^PCA)]^−^ and [Cu^II^(OAr)(^X^PPEDC)]^+^ both display chromophores composed of the two moderately intense features at around 490–540 and 700–740 nm, respectively.

Unfortunately, the [Cu^II^(OAr-X)(**1**)]^+^ complexes are not thermally stable, which prevented their characterization by X-ray crystallography and collection of informative electrospray ionization mass spectrometry (ESI-MS) data. However, independent preparation, by the combination of [Cu^II^(**1**)(NCMe)(OH_2_)]^2+^ with X-ArOK (at −90 °C), afforded complexes possessing very similar UV–vis and EPR spectra (Figures S26 and S28, respectively). Additionally, a DFT exploration of the potential energy surface of four-coordinate [Cu^II^(OAr-Me)(**1**)]^+^ revealed three energetically close minima corresponding to different phenoxocopper(II) conformers (Figure S37). All three minima display distorted square planar or seesaw geometries (τ_4_ = 0.23–0.48; Table S15), near-axial EPR parameters (Table S21), and TDDFT calculated UV–vis spectra that bear a close resemblance to experiment (Figures S46–S48), wherein the transitions possess significant LMCT character. These results reaffirm our assignment.

Reaction between [Cu^II^(η^1^-O_2_^•–^)(**1**)]^+^ and X-ArOH is expected to proceed either via protonation of the superoxo moiety or by a hydrogen atom transfer (HAT) reaction. Protonation would yield [Cu^II^(OAr-X)(**1**)]^+^ and HO_2_^•^. The latter would likely either disproportionate to 1/2 equiv each of O_2_ and H_2_O_2_, as suggested by Itoh and co-workers,^53^ or react with phenol to yield 1 equiv each of phenoxyl radical, X-ArO^•^, and H_2_O_2_. In contrast, HAT would initially yield a hydroperoxocopper(II) complex, [Cu^II^(OOH)(**1**)]^+^, and X-ArO^•^ (eq 3). Subsequent, rapid protonolysis of [Cu^II^(OOH)(**1**)]^+^ by comparatively acidic X-ArOH would provide free H_2_O_2_ and experimentally observed [Cu^II^(OAr-X)(**1**)]^+^ (eq 4). In essence, protonolysis and reaction via HAT might yield 1 equiv of both X-ArO^•^ and H_2_O_2_, which would make them indistinguishable on the basis of product stoichiometry.

**Scheme 1:**
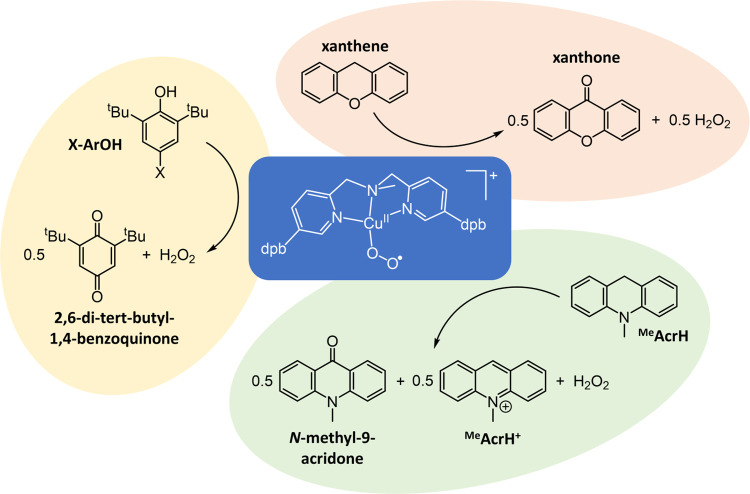
Summary of the Substrates Used in the Reaction Kinetic Studies with [Cu^II^(η^1^-O_2_^•–^)(**1**)]^+^ and the Products Obtained In X-ArOH, X = OMe, MPPO, Me, Et, and *t*Bu. MPPO = 2-methyl-1-phenylpropan-2-yloxy.

To confirm the feasibility of the aforementioned HAT and protonolysis mechanistic pathways, the other products of reactions of [Cu^II^(η^1^-O_2_^•–^)(**1**)]^+^ and X-ArOH were identified and quantified. Consistent with expectations and previous studies of reaction of Cu^II^(η^1^-O_2_^•–^) with phenols,^72,73^ iodometric titration confirmed the presence of 1 equiv of H_2_O_2_ for all X-ArOH substrates (Table S12). In contrast, the formation of X-ArO^•^ was not observed by UV–vis spectroscopy, and it was detected by EPR spectroscopy in only one case (i.e., MeO-ArO^•^), in minor quantities (Figure S27a). Although this would seem to preclude the aforementioned mechanisms, the absence of X-ArO^•^ can result from the rapid conversion to other products. Similar observations were made by Karlin and co-workers for the complex [Cu^II^(η^1^-O_2_^•–^)(DMM-TMPA)]^+^, and were rationalized by invocation of a mechanism revolving around reaction of X-ArO^•^ with a second molecule of [Cu^II^(η^1^-O_2_^•–^)(DMM-TMPA)]^+^.^77^ Instead, 1/2 equiv 2,6-di-*tert*-butyl-1,4-benzoquinone was reportedly formed as the product of reaction. GC-MS analysis of the product mixtures from reaction of [Cu^II^(η^1^-O_2_^•–^)(**1**)]^+^ and X-ArOH yielded a similar outcome, with 1/2 equiv 2,6-di-*tert*-butyl-1,4-benzoquinone (i.e., approximately 50% yield; Scheme 1 and Table S8) being obtained for all X-ArOH substrates.
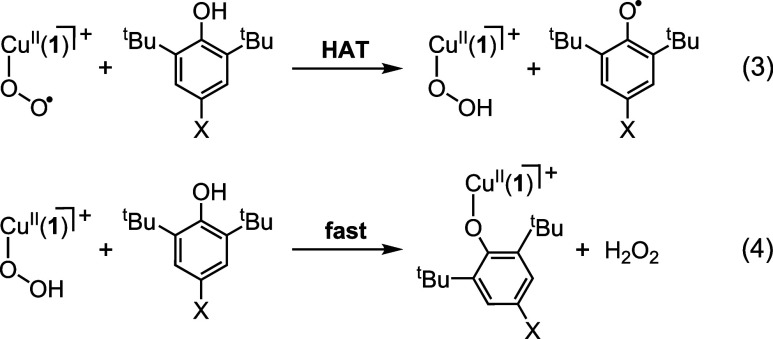
3

The observed pseudo-first-order rate constants (*k*_obs_) derived from the reaction of [Cu^II^(η^1^-O_2_^•–^)(**1**)]^+^ with the various X-ArOH were found to be linearly dependent upon substrate concentration (Figures 3b and S17), thereby yielding second-order rate constants (*k*_2_). These values were found to be significantly larger than those measured for the tetradentate ligand coordinated analogues [Cu^II^(η^1^-O_2_^•–^)(**2**)]^+^ and [Cu^II^(η^1^-O_2_^•–^)(tpb_3_-TMPA)]^+^ (Table S4 and Figure S22), which were previously confirmed to react with X-ArOH via HAT.^56^ For instance, the reaction of [Cu^II^(η^1^-O_2_^•–^)(**1**)]^+^ and [Cu^II^(η^1^-O_2_^•–^)(**2**)]^+^ with MeO-ArOH (at −90 °C) proceeds with *k*_2_ values of 15 and 0.13 M^–1^ s^–1^, respectively. This corresponds to >100-fold faster reaction of [Cu^II^(η^1^-O_2_^•–^)(**1**)]^+^ relative to [Cu^II^(η^1^-O_2_^•–^)(**2**)]^+^.

To determine whether the difference in the oxidative facility of [Cu^II^(η^1^-O_2_^•–^)(**1**)]^+^ and [Cu^II^(η^1^-O_2_^•–^)(**2**)]^+^ originates from a change in mechanism, *k*_2_ values for reaction with the deuterated substrate MeO-ArOD were measured (Figures 3b and S24a) and their primary kinetic isotope effects (KIEs) were calculated. The KIE of 5.9 obtained for [Cu^II^(η^1^-O_2_^•–^)(**1**)]^+^ at −90 °C is very similar to the value of 5.2 measured for [Cu^II^(η^1^-O_2_^•–^)(**2**)]^+^ at −40 °C and is suggestive of rate-determining hydrogen atom abstraction from the phenolic substrate. To verify this conclusion, a Marcus-type plot of log(*k*_2_) values obtained from the reaction of [Cu^II^(η^1^-O_2_^•–^)(**1**)]^+^ with a series of X-ArOH substrates versus the oxidation potential (*E*_ox_) of the substrates was constructed (Figure 3c). For such a plot, a slope with a gradient of 0 would be expected for a pure HAT reaction, whereas an initial discrete e^–^ transfer would yield a value between −0.5 and −1.0.^78−80^ Conversely, given that the acidity of phenols increases in parallel with their *E*_ox_ values, a positive slope would be obtained for a reaction involving an initial discrete proton transfer step (i.e., protonolysis of [Cu^II^(η^1^-O_2_^•–^)(**1**)]^+^). The Marcus-type plot for [Cu^II^(η^1^-O_2_^•–^)(**1**)]^+^ displays a linear correlation possessing a slope of −0.26, which is indicative of a HAT reaction that proceeds via a transition state involving significant charge transfer.^77,81,82^ This is very similar to the value of −0.24 obtained from an analogous plot for the complex [Cu^II^(η^1^-O_2_^•–^)(tpb_3_-TMPA)]^+^, whose reactivity is essentially identical to [Cu^II^(η^1^-O_2_^•–^)(**2**)]^+^.^56^

In essence, the reaction of [Cu^II^(η^1^-O_2_^•–^)(**1**)]^+^ and [Cu^II^(η^1^-O_2_^•–^)(**2**)]^+^ with X-ArOH substrates shares the same general mechanistic properties, with it proceeding via an initial HAT (eq 3). The resulting hydroperoxocopper(II) complex, [Cu^II^(OOH)(**1**)]^+^, then reacts with an additional molecule of X-ArOH (via protonolysis) to give [Cu^II^(OAr-X)(**1**)]^+^ and 1 equiv of H_2_O_2_ as products (eq 4). This differentiates [Cu^II^(η^1^-O_2_^•–^)(**1**)]^+^ from the four-coordinate complexes [Cu^II^(η^1^-O_2_^•–^)(^iPr2^PCA)]^−^ and [Cu^II^(η^1^-O_2_^•–^)(^X^PPEDC)]^+^, which both react with phenols (primarily) via protonolysis of the superoxo ligand.^48,53^

As a means of examining the impact of steric bulk upon the rate of reaction with [Cu^II^(η^1^-O_2_^•–^)(**1**)]^+^, the substrate 4-methoxyphenol was employed. Although this substrate has a much smaller steric profile than the X-ArOH substrates, which might be expected to result in faster reaction, it has a significantly higher bond dissociation enthalpy (BDE) of 87.6 kcal mol^–1^,^83^ which would be expected to retard reaction. For comparison, MeO-ArOH and ^t^Bu-ArOH have BDEs of 82.0 and 85.2 kcal mol^–1^, respectively.^83^ As with the X-ArOH substrates, the reaction of [Cu^II^(η^1^-O_2_^•–^)(**1**)]^+^ with 4-methoxyphenol was found to be first order in both the complex and substrate (Figure S19). Fitting of the data yielded a *k*_2_ of 0.0035 M^–1^ s^–1^ that is ∼10 times lower than the value measured for reaction with ^t^Bu-ArOH. This suggests that the rate of reaction of [Cu^II^(η^1^-O_2_^•–^)(**1**)]^+^ with phenolic substrates is largely controlled by thermodynamic factors, with sterics having little impact. It should be noted that under the same reaction conditions, at −90 °C, [Cu^II^(η^1^-O_2_^•–^)(**2**)]^+^ shows little or no reaction with ^t^Bu-ArOH and more oxidatively resistant phenols. From this, it can be inferred that the thermodynamic driving force for the reaction of [Cu^II^(η^1^-O_2_^•–^)(**1**)]^+^ is greater than [Cu^II^(η^1^-O_2_^•–^)(**2**)]^+^.

### Reaction with C–H Bond Substrates

Reaction of [Cu^II^(η^1^-O_2_^•–^)(**1**)]^+^ with substrates containing weak C–H bonds was also examined, but it presented a more complicated picture. This is because these substrates often possess olefinic functionality that are able to bind to copper(I). Given that O_2_ binding is an equilibrium process, such substrates can cause deoxygenation of [Cu^II^(η^1^-O_2_^•–^)(**1**)]^+^ and formation of a copper(I) olefin complex, [Cu^I^(**1**)(olefin)]^+^. Behavior of this type was observed for the [Cu^II^(η^1^-O_2_^•–^)(Ar_3_-TMPA)]^+^ complexes,^56^ but it is more pronounced in [Cu^II^(η^1^-O_2_^•–^)(**1**)]^+^. For instance, the addition of 10 equiv of 1,4-cyclohexadiene (1,4-CHD) to [Cu^II^(η^1^-O_2_^•–^)(**1**)]^+^ caused near-instantaneous loss of its chromophore and formation of a copper(I) complex (Figure S20b), which is presumed to be [Cu^I^(**1**)(1,4-CHD)]^+^. This complex can be made independently by the addition of 1,4-CHD to a THF solution of [Cu^I^(**1**)(NCMe)]^+^, which caused a change in color from pale yellow to colorless. The UV–vis spectral change associated with this reaction and the ^1^H NMR spectrum of the product are provided in the Supporting Information (Figures S5 and S20c).

A similarly fast reaction was observed (Figure S20a) upon the addition of 1-benzyl-1,4-dihydronicotinamide (BNAH), in place of 1,4-CHD, to [Cu^II^(η^1^-O_2_^•–^)(**1**)]^+^. It was initially assumed that this solely reflected the dissociation of O_2_ and the binding of BNAH in its place. However, reaction workup provided a 41(5) % yield of 1-benzylnicotinamidium (BNA^+^; eq S3 and Table S9). This suggests that binding of O_2_ and BNAH to the copper(I) center are both in equilibrium and small concentrations of [Cu^II^(η^1^-O_2_^•–^)(**1**)]^+^ are present, which rapidly oxidizes BNAH. Given the resulting mechanistic complexity, no efforts were made to deconvolute the kinetic data and, instead, we sought substrates with lower tendencies to bind to copper(I).

A survey of potential substrates containing weak C–H bonds was performed, and *N*-methyl-9,10-dihydroacridine (^Me^AcrH_2_) and xanthene were observed to have a minimal instantaneous impact upon the chromophore of [Cu^II^(η^1^-O_2_^•–^)(**1**)]^+^ (Figure S21), which implies comparatively weak coordination to copper(I). This renders them suitable for the measurement of nonambiguous reaction kinetics. The subsequent slow changes in UV–vis spectroscopic features (that accompany reaction with ^Me^AcrH_2_ and xanthene) display commonalities: the loss of [Cu^II^(η^1^-O_2_^•–^)(**1**)]^+^ and formation of a product containing ligand field transitions typical of copper(II) complexes. EPR spectra of the products obtained from reaction with ^Me^AcrH_2_ and xanthene (Figure S29 and Table S7) both contain near-identical axial signals corresponding to a single copper(II) complex possessing a d_*x*^2^–*y*^2^_ ground state (i.e., *g*_*z*_ > *g*_*x*_ = *g*_*y*_). Given the absence of evidence for the formation of [Cu^II^(OOH)(**1**)]^+^, this is suggested to be a hydroxocopper(II) complex, [Cu^II^(OH)(**1**)]^+^, formed by hydrolysis with adventitious water.

However, there are also readily discernible differences between reactions of [Cu^II^(η^1^-O_2_^•–^)(**1**)]^+^ with ^Me^AcrH_2_ and xanthene. The most obvious is the growth of sharp UV–vis features during reaction with ^Me^AcrH_2_, between 350 and 420 nm, that are indicative of contemporaneous formation of ^Me^AcrH^+^ and *N*-methyl-9-acridone (Figures S21a and S25). Consistent with this, analysis of the corresponding product mixtures confirmed the presence of 1 equiv of H_2_O_2_, 1/2 equiv of *N*-methyl-9-acridone (respective yields of 90(5) and 49(4) %), and, at least, 1/2 equiv of ^Me^AcrH^+^. In contrast, reaction with xanthene produces 1/2 equiv of both xanthone and H_2_O_2_ (yields of 48(4) and 59(5) %, respectively).

Although HAT is almost certainly the rate-determining step in the oxidation of xanthene, the substrate ^Me^AcrH_2_ can function as both a hydrogen atom donor and a hydride donor, with the observed behavior depending on the properties of the acceptor. Hydride donation by ^Me^AcrH_2_ should initially yield only *N*-methyl-acridinium (^Me^AcrH^+^) and, conversely, HAT would be expected to form oxygenated products, including acridone. On this basis alone, we can conclude that the reaction of [Cu^II^(η^1^-O_2_^•–^)(**1**)]^+^ with ^Me^AcrH_2_ also proceeds via HAT. Reaction of Cu^II^(η^1^-O_2_^•–^) complexes with more potent hydride donors than ^Me^AcrH_2_ (i.e., BNAH and BzImH) has also been reported to proceed via HAT.^56,63^ Consistent with expectations for HAT, the reaction of [Cu^II^(η^1^-O_2_^•–^)(**1**)]^+^ with ^Me^AcrH_2_ and xanthene was found to be first-order in both complex and substrates (Figure 3d), and the resulting *k*_2_ values (0.0059 and 0.00065 M^–1^ s^–1^, respectively) are dependent upon the relative C–H bond dissociation enthalpies, BDEs, of the substrates. More specifically, the ∼1 order of magnitude faster reaction with ^Me^AcrH_2_ reflects the smaller BDE of its reactive C–H bond compared to that of xanthene (73.0 and 77.9 kcal mol^–1^, respectively).^83,84^

**Scheme 2:**
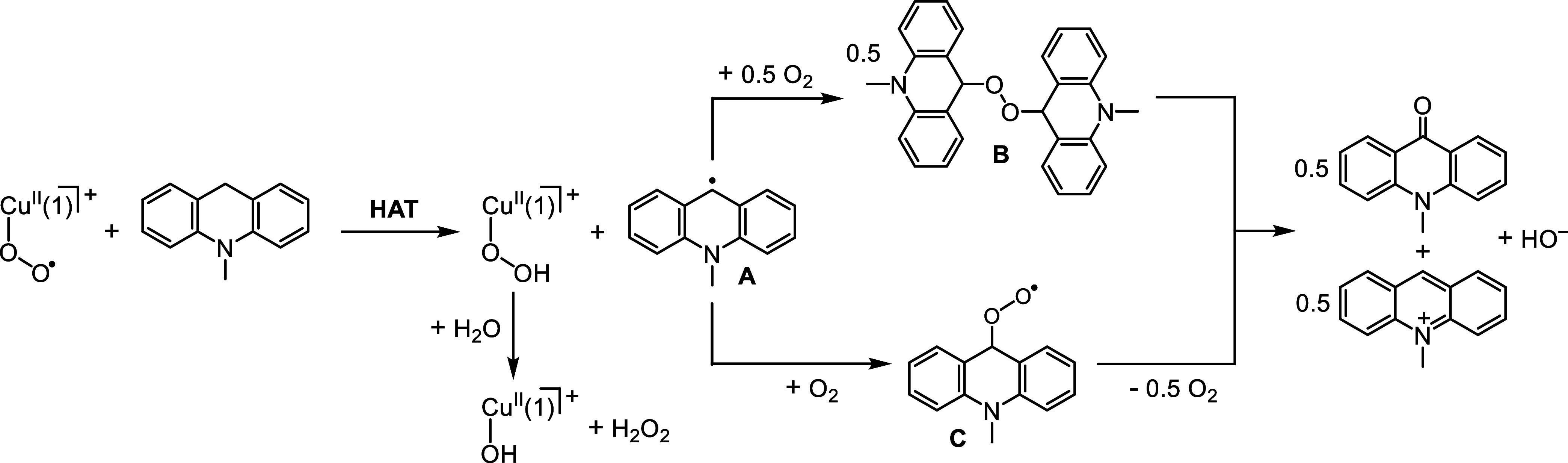
Possible Mechanisms for Equimolar Formation of *N*-Methylacridone and *N*-Methylacridinium from the Reaction of [Cu^II^(η^1^-O_2_^•–^)(**1**)]^+^ with ^Me^AcrH_2_

Stoichiometric formation of H_2_O_2_ from the reaction of [Cu^II^(η^1^-O_2_^•–^)(**1**)]^+^ with ^Me^AcrH_2_ implies that [Cu^II^(η^1^-O_2_^•–^)(**1**)]^+^ is converted solely to [Cu^II^(OOH)(**1**)]^+^ (Scheme 2), which is probably either hydrolyzed during reaction or upon workup. The associated HAT reaction would generate 1 equiv of the *N*-methyl-9-acridinyl radical (**A**), which would then, presumably, react with O_2_ to give either 1 equiv of the *N*-methyl-9-acridinylperoxy radical (**C**) or 1/2 equiv of di(*N*-methyl-9-acridinyl) peroxide (**B**). Disproportionation of the former and heterolytic cleavage of the latter would afford 1/2 equiv each of the *N*-methyl-9-acridone and 9,10-dihydro-10-methyl-9-acridinol. Instantaneous extrusion of the hydroxide anion from 9,10-dihydro-10-methyl-9-acridinol, driven by aromatization, would yield the observed 1/2 equiv ^Me^AcrH^+^.

Lastly, the reaction of [Cu^II^(η^1^-O_2_^•–^)(**2**)]^+^ with substrates containing weak C–H bonds is difficult to study at −90 °C because it is tremendously slow. Consequently, we have measured kinetics for these reactions only at −40 °C (Figures S23 and S24b). Even at this temperature, [Cu^II^(η^1^-O_2_^•–^)(**2**)]^+^ displayed no observable reaction with xanthene, and the reaction with BNAH and ^Me^AcrH_2_ proceeded slowly, affording respective *k*_2_ values of 0.14 and 0.00094 M^–1^ s^–1^. The latter *k*_2_ value is approximately 6 times smaller than that measured for [Cu^II^(η^1^-O_2_^•–^)(**1**)]^+^ at −90 °C. Taking the temperature difference into account, it is estimated that [Cu^II^(η^1^-O_2_^•–^)(**1**)]^+^ reacts with ^Me^AcrH_2_ ∼ 200 times faster than [Cu^II^(η^1^-O_2_^•–^)(**2**)]^+^.

## Conclusions

In previously published BPA-supported systems, mononuclear Cu–O_2_ adducts have been invoked as intermediates in the formation of dicopper-O_2_ adducts, **P** and **O**. However, they had never been directly observed, which left questions regarding their binding mode, electronic structure, and reactivity properties. Herein, by judicious incorporation of large aryl substituents onto the BPA ligand framework, we have developed a four-coordinate copper(I) complex, [Cu^I^(**1**)(NCMe)]^+^, that binds O_2_ to give a superoxocopper(II) complex that is stable against collapse to higher nuclearity **P** or **O** species. This superoxocopper(II) complex was spectroscopically and computationally characterized, and it is concluded that it is end-on bound and is thusly formulated as [Cu^II^(η^1^-O_2_^•–^)(**1**)]^+^. There are only a handful of four-coordinate Cu^II^(η^1^-O_2_^•–^) model complexes, with the vast majority reported over the past 30+ years being five-coordinate species. Interestingly, all of these four-coordinate complexes have geometries that are best described as seesaw, with all but one being closer to square planar than tetrahedral.

The ligand design strategy employed in this study was previously used in a series of tetradentate TMPA ligands and allowed us to synthesize a series of highly stable five-coordinate Cu^II^(η^1^-O_2_^•–^) complexes. This included **2**, which bears the same aryl substituents as **1**. The similarity of these two ligands, which differ by a single pyridine donor, provides a hitherto unavailable opportunity to directly compare the inherent reactivity of four- and five-coordinate Cu^II^(η^1^-O_2_^•–^) species. It was discovered that [Cu^II^(η^1^-O_2_^•–^)(**1**)]^+^ reacts with hydrogen atom donor substrates 2 orders of magnitude faster than [Cu^II^(η^1^-O_2_^•–^)(**2**)]^+^. More specifically, the former reacts with MeO-ArOH and ^Me^AcrH_2_ approximately 100 and 200 times faster, respectively, than the latter. Furthermore, [Cu^II^(η^1^-O_2_^•–^)(**1**)]^+^ is observed to react with the substrates 4-methoxyphenol and xanthene, which possess stronger O–H and C–H bonds, whereas [Cu^II^(η^1^-O_2_^•–^)(**2**)]^+^ cannot. From these observations, it can be inferred that the thermodynamic driving force for HAT to [Cu^II^(η^1^-O_2_^•–^)(**1**)]^+^ is greater than that to [Cu^II^(η^1^-O_2_^•–^)(**2**)]^+^. This is presumably due to the greater inherent electrophilicity of the four-coordinate center, which is a natural result of having a reduced number of ligand donors, and is evident in a 110 mV more positive Cu^II^/Cu^I^ redox potential of [Cu^I^(**1**)(NCMe)]^+^ compared with [Cu^I^(**2**)(NCMe)]^+^. Similar parallels can be (or have been) drawn between Cu^II/I^ reduction potentials and the reactivity of five-coordinate Cu^II^(η^1^-O_2_^•–^) complexes. For instance, Karlin and co-workers have shown, in two separate cases, that replacement of a nitrogen donor in a tripodal tetradentate N_4_ ligands by a thioether not only causes a significant increase in Cu^II^/Cu^I^ redox potential but also leads to enhanced substrate reactivity.^67,85^ Similarly, the precursors to the four-coordinate complexes [Cu^II^(η^1^-O_2_^•–^)(^X^PPEDC)]^+^ and [Cu^II^(η^1^-O_2_^•–^)(PPMDC)]^+^ have Cu^II^/Cu^I^ redox potentials of 0.4 and 0.17 V (vs SCE), respectively, and whereas the former is able to oxidize ferrocenes and intramolecularly hydroxylate the ligand’s phenylethyl substituent, the latter displays only nucleophilic reactivity.^45,51^

Nevertheless, there is wide variation between the reactivity of published Cu^II^(η^1^-O_2_^•–^) complexes, with some four- and five-coordinate species displaying greater HAT reactivity than others. This presumably arises from the nature of the supporting ligands, with charge appearing to be a major contributing factor, which implies that ligand design can be used to further enhance/tune reactivity. Crucially, we anticipate our observations of higher reactivity in similarly ligated four- vs five-coordinate Cu^II^(η^1^-O_2_^•–^) complexes will hold true elsewhere. By extension, it is likely no coincidence that four-coordinate Cu^II^(η^1^-O_2_^•–^) intermediates, not their five-coordinate congeners, are believed to be the active oxidants in noncoupled binuclear copper monooxygenases and FGE, where high oxidative capacities are required for hydroxylation of C–H bonds.

## Supporting Information Available

Experimental and synthetic procedures; X-ray crystallographic data collection and structural parameters; cyclic voltammetry; additional resonance Raman; XAS; and UV–vis spectra; reaction kinetics data; and full computational results (PDF)
DFT calculated atomic coordinates (XYZ)

## Supplementary Material

ja3c12268_si_001.pdf

ja3c12268_si_002.xyz

## References

[ref1] Solomon E. I.; Heppner D. E.; Johnston E. M.; Ginsbach J. W.; Cirera J.; Qayyum M.; Kieber-Emmons M. T.; Kjaergaard C. H.; Hadt R. G.; Tian L. Copper Active Sites in Biology. Chem. Rev. 2014, 114, 3659–3853. 10.1021/cr400327t. 24588098 PMC4040215

[ref2] Quist D. A.; Diaz D. E.; Liu J. J.; Karlin K. D. Activation of dioxygen by copper metalloproteins and insights from model complexes. J. Biol. Inorg. Chem. 2017, 22, 253–288. 10.1007/s00775-016-1415-2. 27921179 PMC5600896

[ref3] Klinman J. P. The copper-enzyme family of dopamine β-monooxygenase and peptidylglycine α-hydroxylating monooxygenase: Resolving the chemical pathway for substrate hydroxylation. J. Biol. Chem. 2006, 281, 3013–3016. 10.1074/jbc.R500011200. 16301310

[ref4] Appel M. J.; Bertozzi C. R. Formylglycine, a Post-Translationally Generated Residue with Unique Catalytic Capabilities and Biotechnology Applications. ACS Chem. Biol. 2015, 10, 72–84. 10.1021/cb500897w. 25514000 PMC4492166

[ref5] Leisinger F.; Miarzlou D. A.; Seebeck F. P. Non-Coordinative Binding of O_2_ at the Active Center of a Copper-Dependent Enzyme. Angew. Chem., Int. Ed. 2021, 60, 6154–6159. 10.1002/anie.202014981. 33245183

[ref6] Appel M. J.; Meier K. K.; Lafrance-Vanasse J.; Lim H.; Tsai C.-L.; Hedman B.; Hodgson K. O.; Tainer J. A.; Solomon E. I.; Bertozzi C. R. Formylglycine-generating enzyme binds substrate directly at a mononuclear Cu(I) center to initiate O_2_ activation. Proc. Natl. Acad. Sci. U.S.A. 2019, 116, 5370–5375. 10.1073/pnas.1818274116. 30824597 PMC6431200

[ref7] Miarzlou D. A.; Leisinger F.; Joss D.; Häussinger D.; Seebeck F. P. Structure of formylglycine-generating enzyme in complex with copper and a substrate reveals an acidic pocket for binding and activation of molecular oxygen. Chem. Sci. 2019, 10, 7049–7058. 10.1039/C9SC01723B. 31588272 PMC6676471

[ref8] Meury M.; Knop M.; Seebeck F. P. Structural Basis for Copper–Oxygen Mediated C–H Bond Activation by the Formylglycine-Generating Enzyme. Angew. Chem., Int. Ed. 2017, 56, 8115–8119. 10.1002/anie.201702901. 28544744

[ref9] Vendelboe T. V.; Harris P.; Zhao Y.; Walter T. S.; Harlos K.; El Omari K.; Christensen H. E. M. The crystal structure of human dopamine β-hydroxylase at 2.9 Å resolution. Sci. Adv. 2016, 2, e1500980 10.1126/sciadv.1500980. 27152332 PMC4846438

[ref10] Cowley R. E.; Tian L.; Solomon E. I. Mechanism of O_2_ activation and substrate hydroxylation in noncoupled binuclear copper monooxygenases. Proc. Natl. Acad. Sci. U.S.A. 2016, 113, 12035–12040. 10.1073/pnas.1614807113. 27790986 PMC5087064

[ref11] Chen P.; Solomon E. I. Oxygen Activation by the Noncoupled Binuclear Copper Site in Peptidylglycine α-Hydroxylating Monooxygenase. Reaction Mechanism and Role of the Noncoupled Nature of the Active Site. J. Am. Chem. Soc. 2004, 126, 4991–5000. 10.1021/ja031564g. 15080705

[ref12] Welch E. F.; Rush K. W.; Arias R. J.; Blackburn N. J. Copper monooxygenase reactivity: Do consensus mechanisms accurately reflect experimental observations?. J. Inorg. Biochem. 2022, 231, 111780 10.1016/j.jinorgbio.2022.111780. 35303611 PMC9183205

[ref13] Welch E. F.; Rush K. W.; Arias R. J.; Blackburn N. J. Pre-Steady-State Reactivity of Peptidylglycine Monooxygenase Implicates Ascorbate in Substrate Triggering of the Active Conformer. Biochemistry 2022, 61, 665–677. 10.1021/acs.biochem.2c00080. 35380039 PMC9064607

[ref14] Rush K. W.; Eastman K. A. S.; Welch E. F.; Bandarian V.; Blackburn N. J. Capturing the Binuclear Copper State of Peptidylglycine Monooxygenase Using a Peptidyl-Homocysteine Lure. J. Am. Chem. Soc. 2024, 146, 5074–5080. 10.1021/jacs.3c14705. 38363651 PMC11096088

[ref15] Wu P.; Fan F.; Song J.; Peng W.; Liu J.; Li C.; Cao Z.; Wang B. Theory Demonstrated a “Coupled” Mechanism for O_2_ Activation and Substrate Hydroxylation by Binuclear Copper Monooxygenases. J. Am. Chem. Soc. 2019, 141, 19776–19789. 10.1021/jacs.9b09172. 31746191

[ref16] Prigge S. T.; Eipper B. A.; Mains R. E.; Amzel L. M. Dioxygen Binds End-On to Mononuclear Copper in a Precatalytic Enzyme Complex. Science 2004, 304, 864–867. 10.1126/science.1094583. 15131304

[ref17] Schröder G. C.; O’Dell W. B.; Webb S. P.; Agarwal P. K.; Meilleur F. Capture of activated dioxygen intermediates at the copper-active site of a lytic polysaccharide monooxygenase. Chem. Sci. 2022, 13, 13303–13320. 10.1039/D2SC05031E. 36507176 PMC9683017

[ref18] Hagemann M. M.; Hedegård E. D. Molecular Mechanism of Substrate Oxidation in Lytic Polysaccharide Monooxygenases: Insight from Theoretical Investigations. Chem. - Eur. J. 2023, 29, e202202379 10.1002/chem.202202379. 36207279 PMC10107554

[ref19] Bissaro B.; Eijsink V. G. H. Lytic polysaccharide monooxygenases: enzymes for controlled and site-specific Fenton-like chemistry. Essays Biochem. 2023, 67, 575–584. 10.1042/EBC20220250. 36734231 PMC10154617

[ref20] Forsberg Z.; Sørlie M.; Petrović D.; Courtade G.; Aachmann F. L.; Vaaje-Kolstad G.; Bissaro B.; Røhr Å. K.; Eijsink V. G. H. Polysaccharide degradation by lytic polysaccharide monooxygenases. Curr. Opin. Struct. Biol. 2019, 59, 54–64. 10.1016/j.sbi.2019.02.015. 30947104

[ref21] Wang B.; Walton P. H.; Rovira C. Molecular Mechanisms of Oxygen Activation and Hydrogen Peroxide Formation in Lytic Polysaccharide Monooxygenases. ACS Catal. 2019, 9, 4958–4969. 10.1021/acscatal.9b00778. 32051771 PMC7007194

[ref22] Bissaro B.; Várnai A.; Åsmund K. R.; Eijsink V. G. H. Oxidoreductases and Reactive Oxygen Species in Conversion of Lignocellulosic Biomass. Microbiol. Mol. Biol. Rev. 2018, 82, e00029-18 10.1128/MMBR.00029-18. 30257993 PMC6298611

[ref23] Tandrup T.; Frandsen K. E. H.; Johansen K. S.; Berrin J.-G.; Lo Leggio L. Recent insights into lytic polysaccharide monooxygenases (LPMOs). Biochem. Soc. Trans. 2018, 46, 1431–1447. 10.1042/BST20170549. 30381341

[ref24] Meier K. K.; Jones S. M.; Kaper T.; Hansson H.; Koetsier M. J.; Karkehabadi S.; Solomon E. I.; Sandgren M.; Kelemen B. Oxygen Activation by Cu LPMOs in Recalcitrant Carbohydrate Polysaccharide Conversion to Monomer Sugars. Chem. Rev. 2018, 118, 2593–2635. 10.1021/acs.chemrev.7b00421. 29155571 PMC5982588

[ref25] Kim B.; Karlin K. D. Ligand–Copper(I) Primary O_2_-Adducts: Design, Characterization, and Biological Significance of Cupric–Superoxides. Acc. Chem. Res. 2023, 56, 2197–2212. 10.1021/acs.accounts.3c00297. 37527056 PMC11152209

[ref26] Elwell C. E.; Gagnon N. L.; Neisen B. D.; Dhar D.; Spaeth A. D.; Yee G. M.; Tolman W. B. Copper-Oxygen Complexes Revisited: Structures, Spectroscopy, and Reactivity. Chem. Rev. 2017, 117, 2059–2107. 10.1021/acs.chemrev.6b00636. 28103018 PMC5963733

[ref27] Mirica L. M.; Ottenwaelder X.; Stack T. D. P. Structure and Spectroscopy of Copper-Dioxygen Complexes. Chem. Rev. 2004, 104, 1013–1045. 10.1021/cr020632z. 14871148

[ref28] Itoh S.; Tachi Y. Structure and O_2_-reactivity of copper(I) complexes supported by pyridylalkylamine ligands. Dalton Trans. 2006, 4531–4538. 10.1039/b607964d. 17016563

[ref29] Osako T.; Ueno Y.; Tachi Y.; Itoh S. Structures and Redox Reactivities of Copper Complexes of (2-Pyridyl)alkylamine Ligands. Effects of the Alkyl Linker Chain Length. Inorg. Chem. 2003, 42, 8087–8097. 10.1021/ic034958h. 14632530

[ref30] Lucas H. R.; Li L.; Sarjeant A. A. N.; Vance M. A.; Solomon E. I.; Karlin K. D. Toluene and Ethylbenzene Aliphatic C-H Bond Oxidations Initiated by a Dicopper(II)-μ-1,2-Peroxo Complex. J. Am. Chem. Soc. 2009, 131, 3230–3245. 10.1021/ja807081d. 19216527 PMC2765497

[ref31] Tahsini L.; Kotani H.; Lee Y.-M.; Cho J.; Nam W.; Karlin K. D.; Fukuzumi S. Electron-Transfer Reduction of Dinuclear Copper Peroxo and Bis-μ-oxo Complexes Leading to the Catalytic Four-Electron Reduction of Dioxygen to Water. Chem. - Eur. J. 2012, 18, 1084–1093. 10.1002/chem.201103215. 22237962 PMC3316124

[ref32] Kunishita A.; Osako T.; Tachi Y.; Teraoka J.; Itoh S. Syntheses, structures, and O_2_-reactivities of copper(I) complexes with bis(2-pyridylmethyl)amine and bis(2-quinolylmethyl)amine tridentate ligands. Bull. Chem. Soc. Jpn. 2006, 79, 1729–1741. 10.1246/bcsj.79.1729.

[ref33] Osako T.; Terada S.; Tosha T.; Nagatomo S.; Furutachi H.; Fujinami S.; Kitagawa T.; Suzuki M.; Itoh S. Structure and dioxygen-reactivity of copper(I) complexes supported by bis(6-methylpyridin-2-ylmethyl)amine tridentate ligands. Dalton Trans. 2005, 3514–3521. 10.1039/b500202h. 16234933

[ref34] Lucas H. R.; Meyer G. J.; Karlin K. D. CO and O_2_ Binding to Pseudo-tetradentate Ligand-Copper(I) Complexes with a Variable N-Donor Moiety: Kinetic/Thermodynamic Investigation Reveals Ligand-Induced Changes in Reaction Mechanism. J. Am. Chem. Soc. 2010, 132, 12927–12940. 10.1021/ja104107q. 20726586 PMC2952189

[ref35] Fujisawa K.; Tanaka M.; Moro-oka Y.; Kitajima N. A Monomeric Side-On Superoxocopper(II) Complex: Cu(O_2_)(HB(3-tBu-5-iPrpz)_3_). J. Am. Chem. Soc. 1994, 116, 12079–12080. 10.1021/ja00105a069.

[ref36] Spencer D. J. E.; Aboelella N. W.; Reynolds A. M.; Holland P. L.; Tolman W. B. β-Diketiminate Ligand Backbone Structural Effects on Cu(I)/O_2_ Reactivity: Unique Copper-Superoxo and Bis(μ-oxo) Complexes. J. Am. Chem. Soc. 2002, 124, 2108–2109. 10.1021/ja017820b. 11878952

[ref37] Aboelella N. W.; Lewis E. A.; Reynolds A. M.; Brennessel W. W.; Cramer C. J.; Tolman W. B. Snapshots of Dioxygen Activation by Copper: The Structure of a 1:1 Cu/O_2_ Adduct and Its Use in Syntheses of Asymmetric Bis(μ-oxo) Complexes. J. Am. Chem. Soc. 2002, 124, 10660–10661. 10.1021/ja027164v. 12207513

[ref38] Reynolds A. M.; Gherman B. F.; Cramer C. J.; Tolman W. B. Characterization of a 1:1 Cu-O_2_ Adduct Supported by an Anilido Imine Ligand. Inorg. Chem. 2005, 44, 6989–6997. 10.1021/ic050280p. 16180861

[ref39] Iovan D. A.; Wrobel A. T.; McClelland A. A.; Scharf A. B.; Edouard G. A.; Betley T. A. Reactivity of a stable copper–dioxygen complex. Chem. Commun. 2017, 53, 10306–10309. 10.1039/C7CC05014C. PMC5605793 28869644

[ref40] Carsch K. M.; Iliescu A.; McGillicuddy R. D.; Mason J. A.; Betley T. A. Reversible Scavenging of Dioxygen from Air by a Copper Complex. J. Am. Chem. Soc. 2021, 143, 18346–18352. 10.1021/jacs.1c10254. 34672573 PMC9351470

[ref41] Sarangi R.; Aboelella N.; Fujisawa K.; Tolman W. B.; Hedman B.; Hodgson K. O.; Solomon E. I. X-ray Absorption Edge Spectroscopy and Computational Studies on LCuO_2_ Species: Superoxide-Cu^II^ versus Peroxide-Cu^III^ Bonding. J. Am. Chem. Soc. 2006, 128, 8286–8296. 10.1021/ja0615223. 16787093 PMC2556900

[ref42] Aboelella N. W.; Kryatov S. V.; Gherman B. F.; Brennessel W. W.; Young V. G. Jr.; Sarangi R.; Rybak-Akimova E. V.; Hodgson K. O.; Hedman B.; Solomon E. I.; Cramer C. J.; Tolman W. B. Dioxygen Activation at a Single Copper Site: Structure, Bonding, and Mechanism of Formation of 1:1 Cu-O_2_ Adducts. J. Am. Chem. Soc. 2004, 126, 16896–16911. 10.1021/ja045678j. 15612729

[ref43] Donoghue P. J.; Gupta A. K.; Boyce D. W.; Cramer C. J.; Tolman W. B. An Anionic, Tetragonal Copper(II) Superoxide Complex. J. Am. Chem. Soc. 2010, 132, 15869–15871. 10.1021/ja106244k. 20977226 PMC3013377

[ref44] Kunishita A.; Kubo M.; Sugimoto H.; Ogura T.; Sato K.; Takui T.; Itoh S. Mononuclear Copper(II)-Superoxo Complexes that Mimic the Structure and Reactivity of the Active Centers of PHM and DβM. J. Am. Chem. Soc. 2009, 131, 2788–2789. 10.1021/ja809464e. 19209864

[ref45] Abe T.; Hori Y.; Shiota Y.; Ohta T.; Morimoto Y.; Sugimoto H.; Ogura T.; Yoshizawa K.; Itoh S. Cupric-superoxide complex that induces a catalytic aldol reaction-type C–C bond formation. Commun. Chem. 2019, 2, 12 10.1038/s42004-019-0115-6.

[ref46] Schön F.; Biebl F.; Greb L.; Leingang S.; Grimm-Lebsanft B.; Teubner M.; Buchenau S.; Kaifer E.; Rübhausen M. A.; Himmel H.-J. On the Metal Cooperativity in a Dinuclear Copper–Guanidine Complex for Aliphatic C–H Bond Cleavage by Dioxygen. Chem. - Eur. J. 2019, 25, 11257–11268. 10.1002/chem.201901906. 31131927

[ref47] Czaikowski M. E.; McNeece A. J.; Boyn J.-N.; Jesse K. A.; Anferov S. W.; Filatov A. S.; Mazziotti D. A.; Anderson J. S. Generation and Aerobic Oxidative Catalysis of a Cu(II) Superoxo Complex Supported by a Redox-Active Ligand. J. Am. Chem. Soc. 2022, 144, 15569–15580. 10.1021/jacs.2c04630. 35977083 PMC10017013

[ref48] Bailey W. D.; Dhar D.; Cramblitt A. C.; Tolman W. B. Mechanistic Dichotomy in Proton-Coupled Electron-Transfer Reactions of Phenols with a Copper Superoxide Complex. J. Am. Chem. Soc. 2019, 141, 5470–5480. 10.1021/jacs.9b00466. 30907590 PMC6584633

[ref49] Bailey W. D.; Gagnon N. L.; Elwell C. E.; Cramblitt A. C.; Bouchey C. J.; Tolman W. B. Revisiting the Synthesis and Nucleophilic Reactivity of an Anionic Copper Superoxide Complex. Inorg. Chem. 2019, 58, 4706–4711. 10.1021/acs.inorgchem.9b00090. 30901201 PMC6548509

[ref50] Pirovano P.; Magherusan A. M.; McGlynn C.; Ure A.; Lynes A.; McDonald A. R. Nucleophilic Reactivity of a Copper(II)-Superoxide Complex. Angew. Chem., Int. Ed. 2014, 53, 5946–5950. 10.1002/anie.201311152. 24753290

[ref51] Itoh S. Developing Mononuclear Copper-Active-Oxygen Complexes Relevant to Reactive Intermediates of Biological Oxidation Reactions. Acc. Chem. Res. 2015, 48, 2066–2074. 10.1021/acs.accounts.5b00140. 26086527

[ref52] Kunishita A.; Ertem M. Z.; Okubo Y.; Tano T.; Sugimoto H.; Ohkubo K.; Fujieda N.; Fukuzumi S.; Cramer C. J.; Itoh S. Active Site Models for the CuA Site of Peptidylglycine α-Hydroxylating Monooxygenase and Dopamine β-Monooxygenase. Inorg. Chem. 2012, 51, 9465–9480. 10.1021/ic301272h. 22908844

[ref53] Tano T.; Okubo Y.; Kunishita A.; Kubo M.; Sugimoto H.; Fujieda N.; Ogura T.; Itoh S. Redox Properties of a Mononuclear Copper(II)-Superoxide Complex. Inorg. Chem. 2013, 52, 10431–10437. 10.1021/ic401261z. 24004030

[ref54] Noh H.; Cho J. Synthesis, characterization and reactivity of non-heme 1st row transition metal-superoxo intermediates. Coord. Chem. Rev. 2019, 382, 126–144. 10.1016/j.ccr.2018.12.006.

[ref55] Fukuzumi S.; Lee Y.-M.; Nam W. Structure and reactivity of the first-row d-block metal-superoxo complexes. Dalton Trans. 2019, 48, 9469–9489. 10.1039/C9DT01402K. 31112168

[ref56] Quek S. Y.; Debnath S.; Laxmi S.; van Gastel M.; Krämer T.; England J. Sterically Stabilized End-On Superoxocopper(II) Complexes and Mechanistic Insights into Their Reactivity with O–H, N–H, and C–H Substrates. J. Am. Chem. Soc. 2021, 143, 19731–19747. 10.1021/jacs.1c07837. 34783549

[ref57] Yang L.; Powell D. R.; Houser R. P. Structural variation in copper(i) complexes with pyridylmethylamide ligands: structural analysis with a new four-coordinate geometry index, τ_4_. Dalton Trans. 2007, 955–964. 10.1039/B617136B. 17308676

[ref58] Shimazaki Y.; Yokoyama H.; Yamauchi O. Copper(I) Complexes with a Proximal Aromatic Ring: Novel Copper–Indole Bonding. Angew. Chem., Int. Ed. 1999, 38, 2401–2403. 10.1002/(SICI)1521-3773(19990816)38:16<2401::AID-ANIE2401>3.0.CO;2-V. 10458801

[ref59] Lionetti D.; Day M. W.; Agapie T. Metal-templated ligand architectures for trinuclear chemistry: tricopper complexes and their O_2_ reactivity. Chem. Sci. 2013, 4, 785–790. 10.1039/C2SC21758A. 23539341 PMC3607385

[ref60] Li S. T.; Braun-Cula B.; Hoof S.; Dürr M.; Ivanović-Burmazović I.; Limberg C. Ligands with Two Different Binding Sites and O_2_ Reactivity of their Copper(I) Complexes. Eur. J. Inorg. Chem. 2016, 2016, 4017–4027. 10.1002/ejic.201600420.

[ref61] Addison A. W.; Rao T. N.; Reedijk J.; van Rijn J.; Verschoor G. C. Synthesis, structure, and spectroscopic properties of copper(II) compounds containing nitrogen–sulphur donor ligands; the crystal and molecular structure of aqua[1,7-bis(N-methylbenzimidazol-2′-yl)-2,6-dithiaheptane]copper(II) perchlorate. J. Chem. Soc., Dalton Trans. 1984, 1349–1356. 10.1039/DT9840001349.

[ref62] Ribelli T. G.; Rahaman S. M. W.; Daran J. C.; Krys P.; Matyjaszewski K.; Poli R. Effect of Ligand Structure on the Cu^II^–R OMRP Dormant Species and Its Consequences for Catalytic Radical Termination in ATRP. Macromolecules 2016, 49, 7749–7757. 10.1021/acs.macromol.6b01334.

[ref63] Peterson R. L.; Himes R. A.; Kotani H.; Suenobu T.; Tian L.; Siegler M. A.; Solomon E. I.; Fukuzumi S.; Karlin K. D. Cupric Superoxo-Mediated Intermolecular C-H Activation Chemistry. J. Am. Chem. Soc. 2011, 133, 1702–1705. 10.1021/ja110466q. 21265534 PMC3091961

[ref64] Woertink J. S.; Tian L.; Maiti D.; Lucas H. R.; Himes R. A.; Karlin K. D.; Neese F.; Würtele C.; Holthausen M. C.; Bill E.; Sundermeyer J.; Schindler S.; Solomon E. I. Spectroscopic and Computational Studies of an End-on Bound Superoxo-Cu(II) Complex: Geometric and Electronic Factors That Determine the Ground State. Inorg. Chem. 2010, 49, 9450–9459. 10.1021/ic101138u. 20857998 PMC2963092

[ref65] Peterson R. L.; Ginsbach J. W.; Cowley R. E.; Qayyum M. F.; Himes R. A.; Siegler M. A.; Moore C. D.; Hedman B.; Hodgson K. O.; Fukuzumi S.; Solomon E. I.; Karlin K. D. Stepwise Protonation and Electron-Transfer Reduction of a Primary Copper-Dioxygen Adduct. J. Am. Chem. Soc. 2013, 135, 16454–16467. 10.1021/ja4065377. 24164682 PMC3874213

[ref66] Schatz M.; Raab V.; Foxon S. P.; Brehm G.; Schneider S.; Reiher M.; Holthausen M. C.; Sundermeyer J.; Schindler S. Dioxygen complexes: Combined spectroscopic and theoretical evidence for a persistent end-on copper superoxo complex. Angew. Chem., Int. Ed. 2004, 43, 4360–4363. 10.1002/anie.200454125. 15368393

[ref67] Bhadra M.; Transue W. J.; Lim H.; Cowley R. E.; Lee J. Y. C.; Siegler M. A.; Josephs P.; Henkel G.; Lerch M.; Schindler S.; Neuba A.; Hodgson K. O.; Hedman B.; Solomon E. I.; Karlin K. D. A Thioether-Ligated Cupric Superoxide Model with Hydrogen Atom Abstraction Reactivity. J. Am. Chem. Soc. 2021, 143, 3707–3713. 10.1021/jacs.1c00260. 33684290 PMC8023764

[ref68] Lanci M. P.; Smirnov V. V.; Cramer C. J.; Gauchenova E. V.; Sundermeyer J.; Roth J. P. Isotopic Probing of Molecular Oxygen Activation at Copper(I) Sites. J. Am. Chem. Soc. 2007, 129, 14697–14709. 10.1021/ja074620c. 17960903

[ref69] Huber S. M.; Shahi A. R. M.; Aquilante F.; Cramer C. J.; Gagliardi L. What Active Space Adequately Describes Oxygen Activation by a Late Transition Metal? CASPT2 and RASPT2 Applied to Intermediates from the Reaction of O_2_ with a Cu(I)-α-Ketocarboxylate. J. Chem. Theory Comput. 2009, 5, 2967–2976. 10.1021/ct900282m. 26609978

[ref70] Larsson E. D.; Dong G.; Veryazov V.; Ryde U.; Hedegård E. D. Is density functional theory accurate for lytic polysaccharide monooxygenase enzymes?. Dalton Trans. 2020, 49, 1501–1512. 10.1039/C9DT04486H. 31922155

[ref71] Paria S.; Ohta T.; Morimoto Y.; Sugimoto H.; Ogura T.; Itoh S. Structure and Reactivity of Copper Complexes Supported by a Bulky Tripodal N4 Ligand: Copper(I)/Dioxygen Reactivity and Formation of a Hydroperoxide Copper(II) Complex. Z. Anorg. Allg. Chem. 2018, 644, 780–789. 10.1002/zaac.201800083.

[ref72] Bhadra M.; Lee J. Y. C.; Cowley R. E.; Kim S.; Siegler M. A.; Solomon E. I.; Karlin K. D. Intramolecular Hydrogen Bonding Enhances Stability and Reactivity of Mononuclear Cupric Superoxide Complexes. J. Am. Chem. Soc. 2018, 140, 9042–9045. 10.1021/jacs.8b04671. 29957998 PMC6217813

[ref73] Diaz D. E.; Quist D. A.; Herzog A. E.; Schaefer A. W.; Kipouros I.; Bhadra M.; Solomon E. I.; Karlin K. D. Impact of Intramolecular Hydrogen Bonding on the Reactivity of Cupric Superoxide Complexes with O–H and C–H Substrates. Angew. Chem., Int. Ed. 2019, 58, 17572–17576. 10.1002/anie.201908471. PMC6878127 31469942

[ref74] Choi Y. J.; Cho K.-B.; Kubo M.; Ogura T.; Karlin K. D.; Cho J.; Nam W. Spectroscopic and computational characterization of Cu^II^-OOR (R = H or cumyl) complexes bearing a Me_6_-tren ligand. Dalton Trans. 2011, 40, 2234–2241. 10.1039/c0dt01036g. 21258722 PMC3318924

[ref75] Yamaguchi S.; Nagatomo S.; Kitagawa T.; Funahashi Y.; Ozawa T.; Jitsukawa K.; Masuda H. Copper Hydroperoxo Species Activated by Hydrogen-Bonding Interaction with Its Distal Oxygen. Inorg. Chem. 2003, 42, 6968–6970. 10.1021/ic035080x. 14577757

[ref76] Kunishita A.; Scanlon J. D.; Ishimaru H.; Honda K.; Ogura T.; Suzuki M.; Cramer C. J.; Itoh S. Reactions of Copper(II)-H_2_O_2_ Adducts Supported by Tridentate Bis(2-pyridylmethyl)amine Ligands: Sensitivity to Solvent and Variations in Ligand Substitution. Inorg. Chem. 2008, 47, 8222–8232. 10.1021/ic800845h. 18698765

[ref77] Lee J. Y.; Peterson R. L.; Ohkubo K.; Garcia-Bosch I.; Himes R. A.; Woertink J.; Moore C. D.; Solomon E. I.; Fukuzumi S.; Karlin K. D. Mechanistic Insights into the Oxidation of Substituted Phenols via Hydrogen Atom Abstraction by a Cupric-Superoxo Complex. J. Am. Chem. Soc. 2014, 136, 9925–9937. 10.1021/ja503105b. 24953129 PMC4102632

[ref78] Ram M. S.; Hupp J. T. Linear free energy relations for multielectron transfer kinetics: a brief look at the Broensted/Tafel analogy. J. Phys. Chem. A 1990, 94, 2378–2380. 10.1021/j100369a035.

[ref79] Weatherly S. C.; Yang I. V.; Thorp H. H. Proton-Coupled Electron Transfer in Duplex DNA: Driving Force Dependence and Isotope Effects on Electrocatalytic Oxidation of Guanine. J. Am. Chem. Soc. 2001, 123, 1236–1237. 10.1021/ja003788u. 11456681

[ref80] Osako T.; Ohkubo K.; Taki M.; Tachi Y.; Fukuzumi S.; Itoh S. Oxidation mechanism of phenols by dicopper-dioxygen (Cu_2_/O_2_) complexes. J. Am. Chem. Soc. 2003, 125, 11027–11033. 10.1021/ja029380+. 12952484

[ref81] Guttenplan J. B.; Cohen S. G. Triplet energies, reduction potentials, and ionization potentials in carbonyl-donor partial charge-transfer interactions. I.. J. Am. Chem. Soc. 1972, 94, 4040–4042. 10.1021/ja00766a079.

[ref82] Wagner P. J.; Lam H. M. H. Charge-transfer quenching of triplet.alpha.-trifluoroacetophenones. J. Am. Chem. Soc. 1980, 102, 4167–4172. 10.1021/ja00532a031.

[ref83] Warren J. J.; Tronic T. A.; Mayer J. M. Thermochemistry of Proton-Coupled Electron Transfer Reagents and its Implications. Chem. Rev. 2010, 110, 6961–7001. 10.1021/cr100085k. 20925411 PMC3006073

[ref84] Zhu X.-Q.; Zhang M.-T.; Yu A.; Wang C.-H.; Cheng J.-P. Hydride, Hydrogen Atom, Proton, and Electron Transfer Driving Forces of Various Five-Membered Heterocyclic Organic Hydrides and Their Reaction Intermediates in Acetonitrile. J. Am. Chem. Soc. 2008, 130, 2501–2516. 10.1021/ja075523m. 18254624

[ref85] Kim S.; Lee J. Y.; Cowley R. E.; Ginsbach J. W.; Siegler M. A.; Solomon E. I.; Karlin K. D. A N_3_S_(thioether)_-Ligated Cu^II^-Superoxo with Enhanced Reactivity. J. Am. Chem. Soc. 2015, 137, 2796–2799. 10.1021/ja511504n. 25697226 PMC4482613

