# Supplementary material for: A Four-Coordinate End-On Superoxocopper(II) Complex: Probing the Link between Coordination Number and Reactivity
Source: J Am Chem Soc. 2024 Aug 14;146(34):23704–16. doi: 10.1021/jacs.3c12268 (PMC11363018; doi:10.1021/jacs.3c12268)
Supplement: Supplementary file 1 — ja3c12268_si_001.pdf [file ja3c12268_si_001.pdf]

A Four-Coordinate End-On Superoxocopper(II) Complex: Probing the Link Between Coordination Number and Reactivity.

Suman Debnath,[a,‡] Shoba Laxmi,[a,‡] Olivia McCubbin Stepanic,[b] Sebastian Y. Quek,[a] Maurice van Gastel,[c] Serena DeBeer,[b,*] Tobias Krämer,[d,e,*] and Jason England.[a,f,*]

Contents: **Page**

	General Methods	S2
Scheme S1	Synthesis of Ligand and Complexes	S3
Figures S1 – S5	NMR Spectra	S7
Figures S6 – S9	Electrospray Ionization Mass Spectrometry (ESI-MS) of Complexes	S12
Figures S10 & S11<br>Tables S1 – S3	X-ray Crystallography	S16
Figures S12	Cyclic Voltammetry	S19
Figures S13	Resonance Raman Spectroscopy	S20
	XAS Measurements	S21
Figures S14 – S25<br>Table S4	Kinetics Studies for Reaction With Substrates	S22
Figure S26	UV-Vis Spectra of $[Cu^{II}(OAr\text{-}X)(\mathbf{1})]^+$	S32
Figure S27 – S29<br>Tables S5 – S7	EPR Spectra	S33
Eqns S1 – S5<br>Figure S30<br>Tables S8 – S12	Quantification of Products from Reaction With Substrates	S37
Figures S31 – S49<br>Tables S13 – S21	Computational Studies	S42
	References	S82

General Methods

Unless otherwise stated, all reactions were performed under dry and oxygen-free conditions, using a combination of standard Schlenk techniques and an inert atmosphere glovebox. Prior to use, toluene and diethyl ether were treated with sodium-benzophenone, distilled, and degassed. Similarly, pentane was dried by refluxing over sodium metal, distilled, and degassed prior to use. Anhydrous acetonitrile, dichloromethane and tetrahydrofuran were purchased from Kanto Chemical Co., Inc. and used without further purification. Thin-layer chromatography (TLC) was performed using silica gel glass plates. Flash column chromatography was undertaken with Merck silica gel. The compounds 2,6-diphenylbenzeneboronic acid, $[Cu^{I}(\mathbf{2})][B(C_6F_5)_4]$, and $[Cu^{I}(NCMe)_4][B(C_6F_5)_4]$ were synthesized via previously published procedures.[1-2] All other reagents were purchased from commercial vendors.

NMR spectra were recorded using either JEOL ECA400 or Bruker Advance III 400 spectrometers in deuterated solvents, with chemical shift values (ppm) referenced against residual protic solvent peaks. UV-Vis spectroscopic experiments were performed using an Agilent Cary 8454 diode-array spectrophotometer equipped with a Unisoku cryostat. Elemental analysis was conducted using a Perkin Elmer Series II CHNS/O analyser. Mass spectra were recorded using a JEOLT100LP electrospray ionization mass spectrometer (ESI-MS). Respective ion source desolvating chamber and orifice 1 temperatures of 40 and 35 °C were used. The X-band (9.63 GHz) continuous wave (cw) EPR experiments were performed at 30 K (power = 2 mW; modulation amplitude = 0.5 mT) using a Bruker Elexsys E500 EPR spectrometer equipped with a Bruker ST9402 standard resonator, a Bruker dual-mode cavity (ER4116DM), an Oxford Instruments ESR 900 helium flow cryostat, and a Bruker ER-049X Super-X high-sensitivity microwave bridge with integrated microwave frequency counter. The ER032T magnetic field controller was calibrated externally using a Bruker ER035M NMR field probe.

Ligand Synthesis

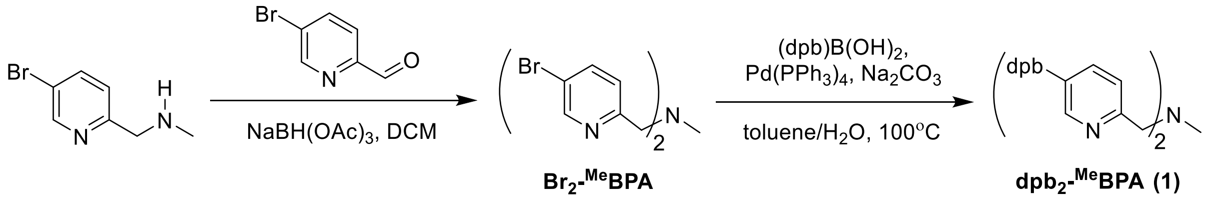

Scheme S1. Synthetic route used in the preparation of dpb$_2$-^{Me}BPA (**1**).

Synthesis of 1-(5-bromopyridin-2-yl)-*N*-methylmethanamine

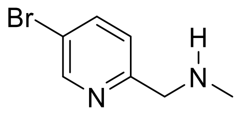

A 40% aqueous solution of methylamine (4.60 mL, 53.3 mmol) was added dropwise to an ice bath cooled solution of 5-bromo-2-pyridinecarboxaldehyde (5.00 g, 26.8 mmol) in methanol (10 mL). The resulting mixture was allowed to warm to room temperature, with continuous stirring for 3 h. Subsequently, it was cooled using an ice bath, once again, and $NaBH_4$ (2.03 g, 53.7 mmol) was added portion-wise to it. After stirring the resulting reaction mixture overnight at room temperature, all solvents were removed using a rotary evaporator, and the residue obtained was dissolved in a mixture of diethyl ether and saturated aqueous sodium sulfite solution. The organic layer was separated, and the basic aqueous layer (pH ≥ 12) was extracted twice with diethyl ether. The combined organic layers were washed with saturated aqueous sodium sulfite solution (20 mL) and brine (20 mL), and dried over magnesium sulfate. Removal of all volatiles using a rotary evaporator gave the crude product as a clear yellow oil (5.10 g, 94 %), which was used without further purification. ^{1}H NMR (400 MHz, $CDCl_3$): δ 1.69 (bs, 1H), 2.46 (s, 3H), 3.82 (s, 2H), 7.22 (dd, 1H, J = 0.6, 8.2 Hz), 7.76 (dd, 1H, J = 2.4, 8.3 Hz), 8.60 (d, 1H, J = 2.3 Hz). ^{13}C NMR (100 MHz, $CDCl_3$): δ 35.75, 56.10, 118.56, 123.37, 138.76, 150.01, 158.00.

Synthesis of *N,N*-bis(5-bromo-2-pyridylmethyl)methylamine (Br$_2$-^{Me}BPA). Sodium triacetoxyborohydride (4.89 g, 22.9 mmol) was added portion-wise to a solution of 5-bromo-2-pyridinecarboxaldehyde (3.15 g, 16.9 mmol) and 1-(5-bromopyridin-2-yl)-*N*-methylmethanamine (3.15 g, 15.7 mmol) in dichloromethane (20 mL), at room temperature.

The resulting mixture was left to stir for 12 h. 2M aqueous sodium hydroxide was subsequently added (20 mL) and the reaction was left to stir for 1 h, after which the reaction mixture was extracted with dichloromethane (50 mL). The organic layer was dried over sodium sulfate, filtered, and all volatiles removed from the filtrate using a rotary evaporator. This gave a dark brown oil, which was dissolved in anhydrous tetrahydrofuran (20 mL) and stirred with 5 equiv. of KH for 1.5 h. After filtration, the filtrate was reduced to dryness. This gave a reddish-brown solid, which was extracted with several portions of hot pentane. The extracts were combined, and the solvent removed using a rotary evaporator to give the product as a pale tan powder (3.95 g, 68 %). ^{1}H NMR (400 MHz, $CDCl_3$): δ 2.30 (s, 3H), 3.70 (s, 4H), 7.39 (d, 2H, J = 8.3 Hz), 7.79 (dd, 2H, J = 2.3, 8.3 Hz), 8.61 (d, 2H, J = 2.2 Hz); ^{13}C NMR (100 MHz, $CDCl_3$): δ 42.83, 62.84, 119.10, 124.40, 139.13, 150.22, 157.75. MS (+ESI): m/z 371.97 $[M+H]^+$ (calculated = 371.95).

Synthesis of *N,N*-bis(5-(2,6-diphenylbenzene)-2-pyridylmethyl)methylamine (dpb$_2$-^{Me}BPA, 1). A three-neck round-bottomed flask fitted with a reflux condenser was charged with Br$_2$-^{Me}BPA (2.25 g, 0.006 mol), 3 equiv. of 2,6-diphenylbenzeneboronic acid (4.92 g, 0.018 mol), 25 mL of toluene, and a magnetic stir bar. Stirring this mixture for 20 min gave a yellowish milky emulsion, to which 25 mL of methanol and 20 mL of a 2 M aqueous solution of sodium carbonate were added. After degassing this biphasic mixture three times by the freeze-pump-thaw method, solid tetrakis(triphenylphosphine)palladium (0.900 g, 0.0006 mol) was added to the flask under a stream of argon, and the resulting reaction mixture was heated at 100°C for 48 h. Subsequent to cooling to room temperature, the organic and aqueous layers were separated, and the aqueous layer was extracted three times with dichloromethane. The combined organic layers were washed three times with 2 M aqueous sodium carbonate solution, dried over sodium sulfate, filtered, and all solvent was removed from the filtrate using a rotary evaporator. This yielded a thick brown oil. Trituration of this oil with hot hexane for 12 h afforded a pale yellow precipitate, which was isolated by filtration and dried under vacuum. Next, the solid was dissolved in diethyl ether, acidified by addition of a few drops of 12 M HCl, and stirred for 3 h. The light-yellow solid obtained was isolated by filtration, dissolved in dichloromethane, and treated with 2 M aqueous sodium hydroxide. The organic layer was separated, washed with 2 M aqueous sodium carbonate solution and brine several times each, dried over sodium sulfate, and reduced to dryness using a rotary evaporator to

give the product (**1**) as a pale beige solid (2.20 g, 55 %). ^{1}H NMR (400 MHz, $CDCl_3$): δ 2.02 (s, 3H), 3.40 (s, 4H), 6.95 (d, 2H, J = 8.0 Hz), 7.03 – 7.12 (m, 22H), 7.45 – 7.47 (m, 4H), 7.50 – 7.54 (m, 2H), 8.00 (d, 2H, J = 1.6 Hz). ^{13}C NMR (100 MHz, $CDCl_3$): δ 42.35, 62.88, 122.26, 126.71, 127.89, 128.10, 129.72, 130.11, 134.05, 135.50, 139.17, 141.30, 142.45, 151.13, 156.29. MS (+ESI): m/z 670.33 $[M+H]^+$ (calculated = 670.32)

Synthesis of Copper Complexes

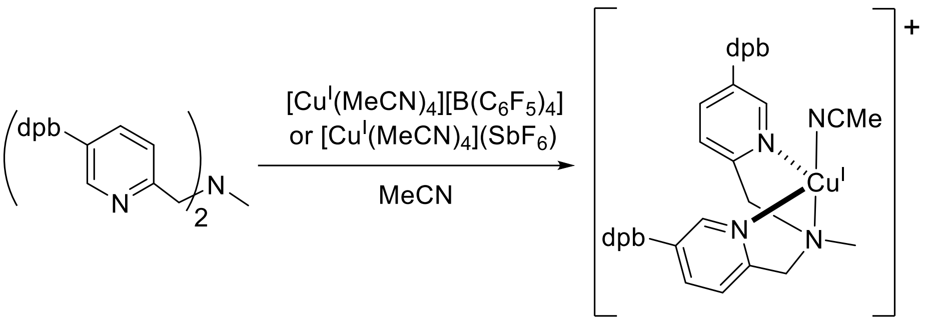

dpb$_2$-^{Me}BPA (1)　　**[Cu^I(1)(MeCN)](X)**

[Cu^I(1)(NCMe)][B(C_6F_5)$_4$]. Equimolar quantities of $[Cu^I(NCMe)_4][B(C_6F_5)_4]$ (0.100 g, 0.110 mmol) and **1** (0.074 g, 0.110 mmol) were dissolved in acetonitrile (5 mL) and stirred for 30 min at room temperature. Subsequently, the reaction mixture was filtered, and the filtrate reduced to dryness in vacuo. This provided the product as a yellow powder (0.159 g, 99 %), which did not require further purification. ^{1}H NMR (400 MHz, CD_3CN): δ 2.23 (s, 3H), 3.39 (s, 4H), 6.98 - 7.14 (m, 22H), 7.33 (d, 2H, J = 8.0 Hz), 7.48 (m, 4H), 7.58 (m, 2H), 7.87 (s, 2H). ^{13}C NMR (100 MHz, $CDCl_3$): δ 44.15, 61.23, 123.39, 127.64, 128.78, 129.59, 130.54, 130.90, 135.10, 137.20, 141.48, 141.95, 143.24, 152.04, 154.75. MS (+ESI): m/z 732.22 [Cu(**1**)]$^+$ (calculated = 732.24).

[Cu^I(1)(NCMe)][SbF_6]. Equimolar quantities of $[Cu^I(NCMe)_4][SbF_6]$ (0.053 g, 0.115 mmol) and **1** (0.077 g, 0.115 mmol) were dissolved in acetonitrile (5 mL) and stirred overnight at room temperature. Subsequently, the reaction mixture was reduced to dryness in vacuo. The solid obtained was triturated with diethyl ether (10 mL) for 3 h, isolated by filtration, and dried in vacuo to give the product as a yellow-green powder (0.082 g, 70.6 %). Single crystals suitable for X-ray crystallography were grown by slow diffusion of diethyl ether into a saturated solution of the complex in acetonitrile. NMR and ESI-MS characterization data were identical to that of [Cu^I(**1**)(NCMe)][B(C_6F_5)$_4$].

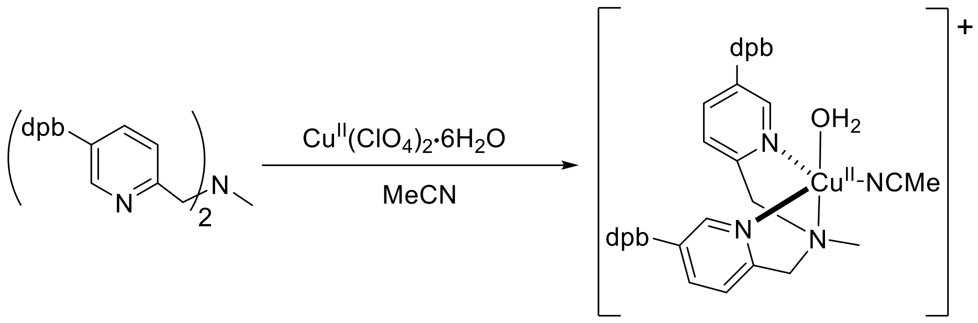

dpb$_2$-^{Me}BPA (1) **[Cu^{II}(1)(NCMe)(OH$_2$)](ClO$_4$)**

[Cu^{II}(1)(NCMe)(OH$_2$)](ClO$_4$)$_2$. A solution of Cu(ClO$_4$)$_2$.6H$_2$O (0.055 g, 0.119 mmol) in acetonitrile (10 mL) was added to a vial containing **1** (0.0795 g, 0.119 mmol) and the resulting mixture was stirred overnight, under ambient conditions. Subsequently, it was filtered, and the volume of the filtrate was reduced (using a rotary evaporator) until precipitate began to form. To spur further precipitation, a copious amount of diethyl ether (50 mL) was added. After stirring for 30 min, the solid obtained was isolated by filtration, washed with diethyl ether, and dried under vacuum to give the product as a blue powder (0.106 g, 92 %). Blue, single crystals suitable for X-ray crystallographic studies were grown by diffusion of diethyl ether vapour into a saturated solution of complex in dichloromethane. MS (+ESI): *m/z* 831.10 [Cu(**1**)(ClO$_4$)]$^+$ (calculated = 831.19). Anal. Calcd. for [Cu(**1**)(CH$_3$CN)(OH$_2$)](ClO$_4$)$_2$ (C$_{51}$H$_{44}$Cl$_2$CuN$_4$O$_9$): C, 61.79; H, 4.47; N, 5.65. Found: C, 61.40; H, 4.78; N, 5.38.

NMR Spectra

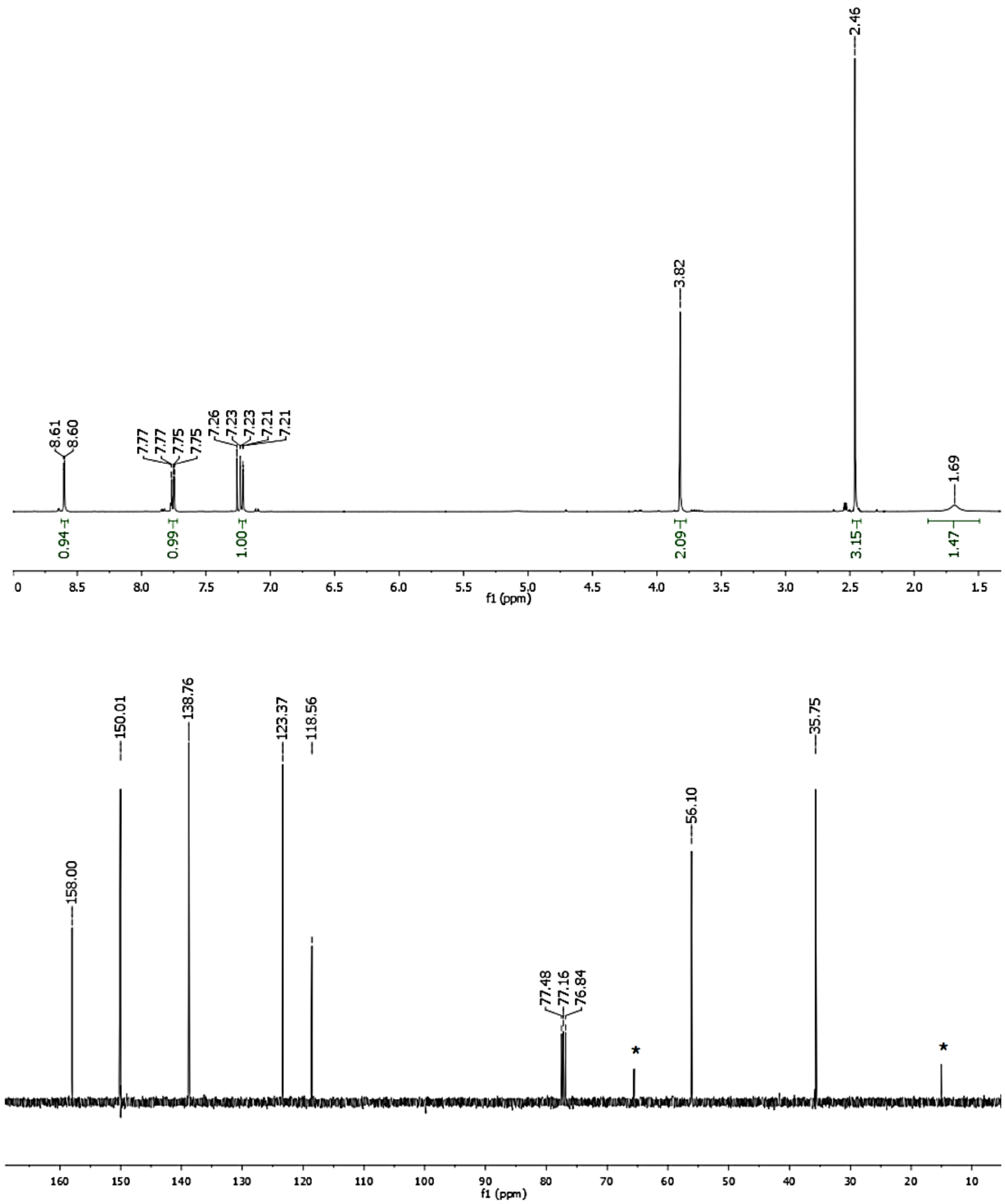

Figure S1. ^{1}H and ^{13}C NMR spectra (top and bottom, respectively) of 5-bromopyridin-2-yl-N-methylmethanamine, in $CDCl_3$ solution at room temperature (* = diethyl ether).

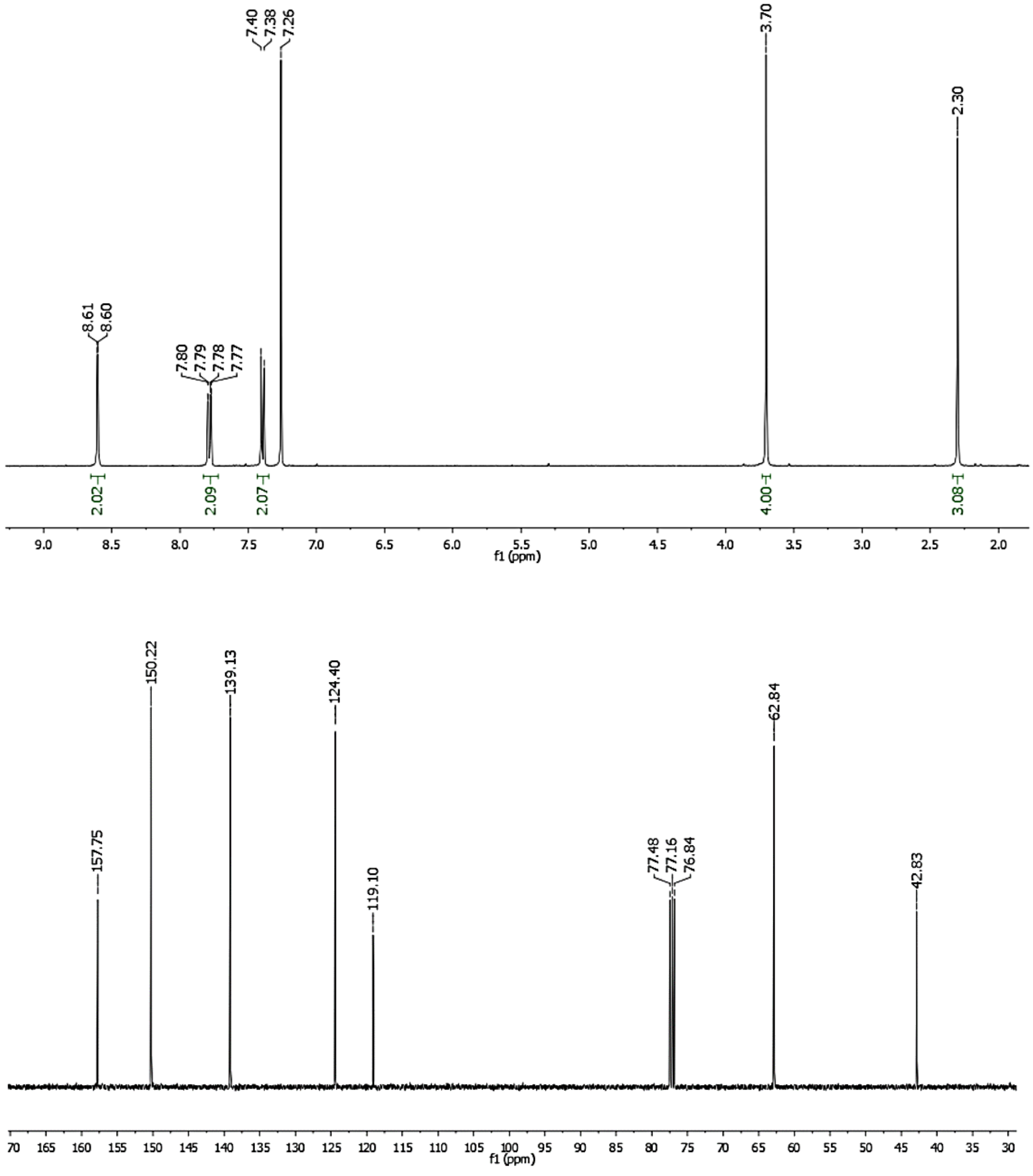

Figure S2. ^{1}H and ^{13}C NMR spectra (top and bottom, respectively) of Br_2-^{Me}BPA, in $CDCl_3$ solution at room temperature.

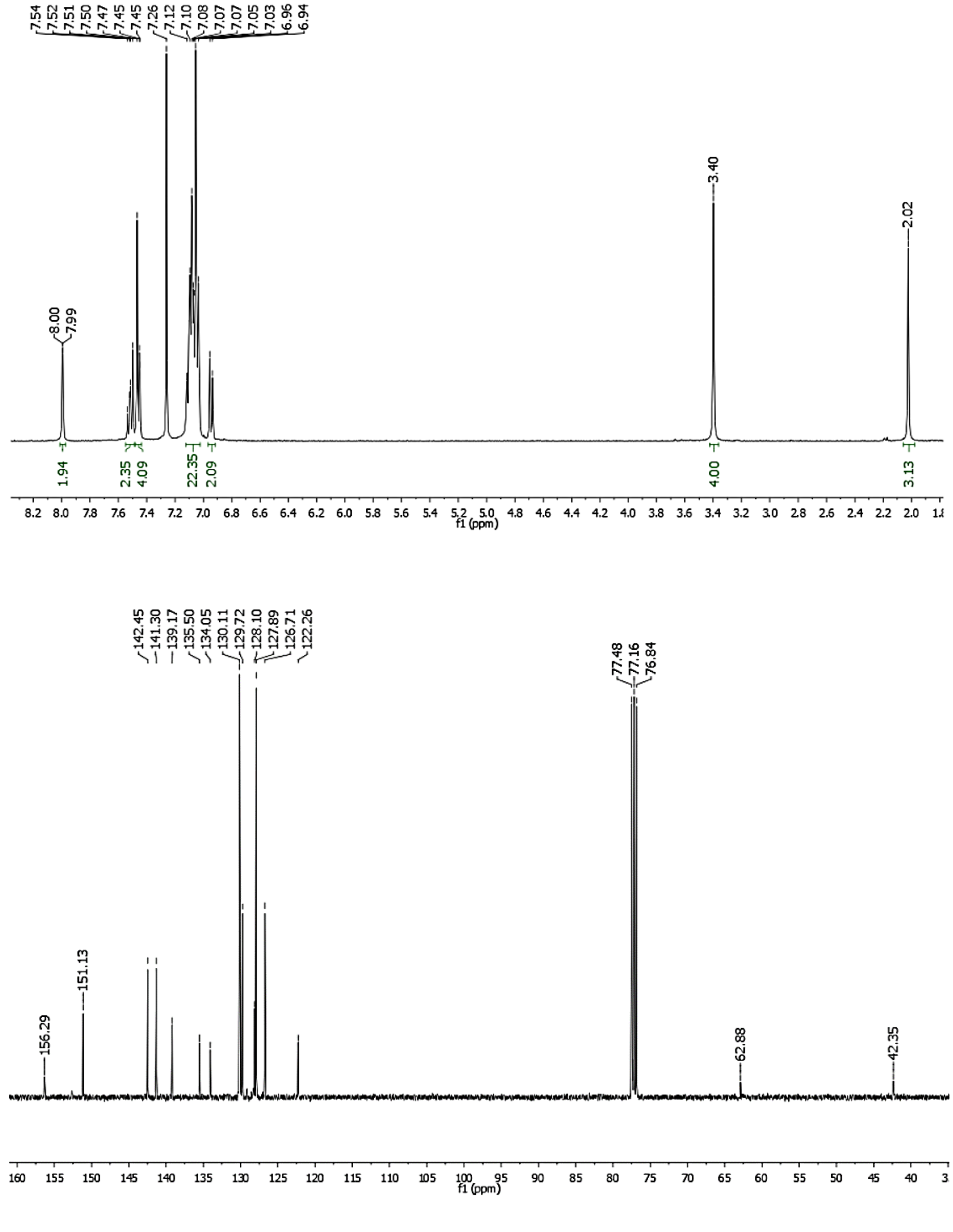

Figure S3. ^{1}H and ^{13}C NMR spectra (top and bottom, respectively) of **1**, in $CDCl_3$ solution at room temperature.

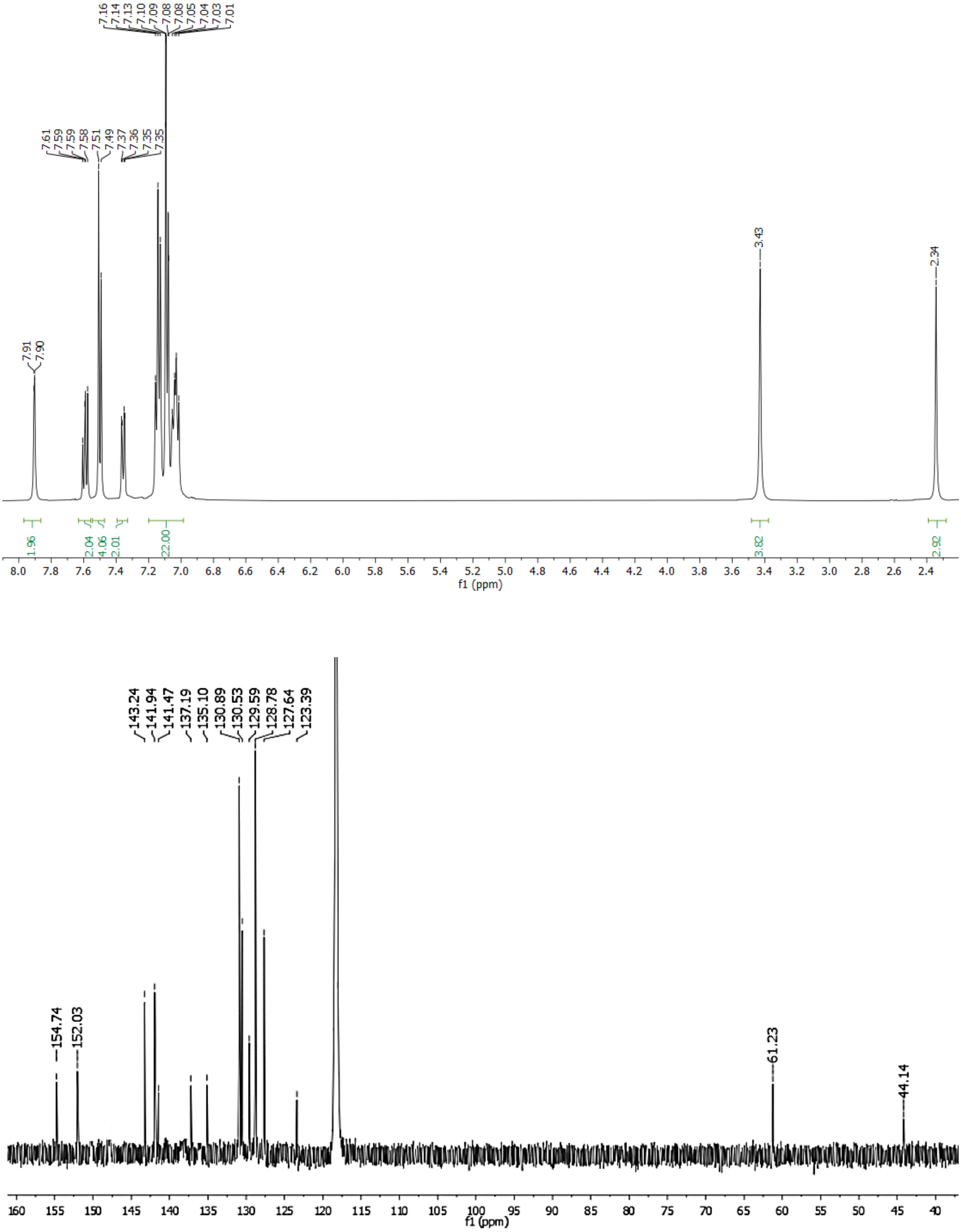

Figure S4. ^{1}H and ^{13}C NMR spectra (top and bottom, respectively) of $[Cu^{I}(\mathbf{1})(NCMe)][B(C_6F_5)_4]$, in CD_3CN solution at room temperature.

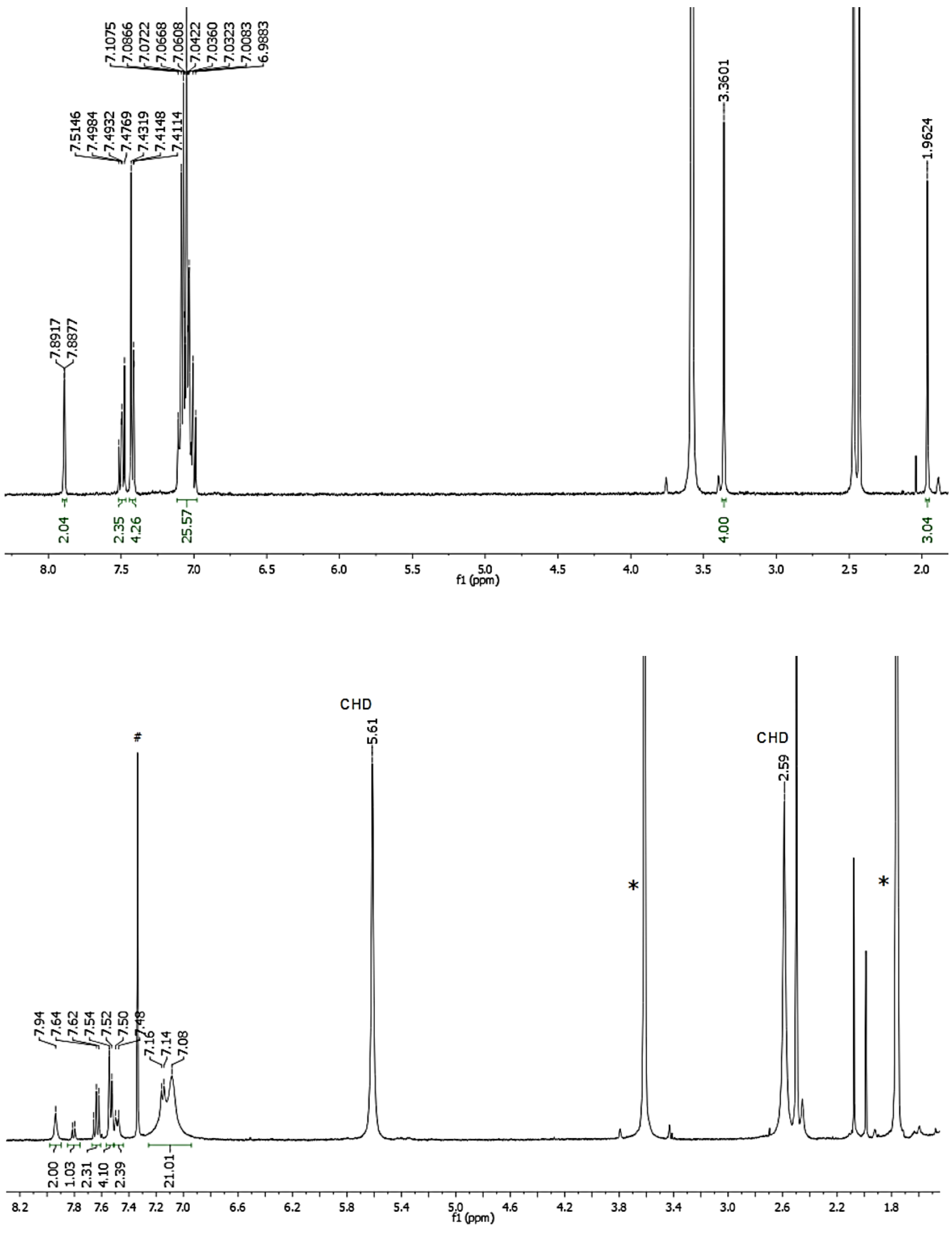

Figure S5. ^{1}H NMR spectra of **1** (top) and [Cu^{I}(**1**)(NCMe)]$^{+}$ + 1,4-CHD (bottom), recorded in THF-d_8 solution at room temperature (* = THF, # = benzene).

Electrospray Ionization Mass Spectrometry (ESI-MS) of Complexes

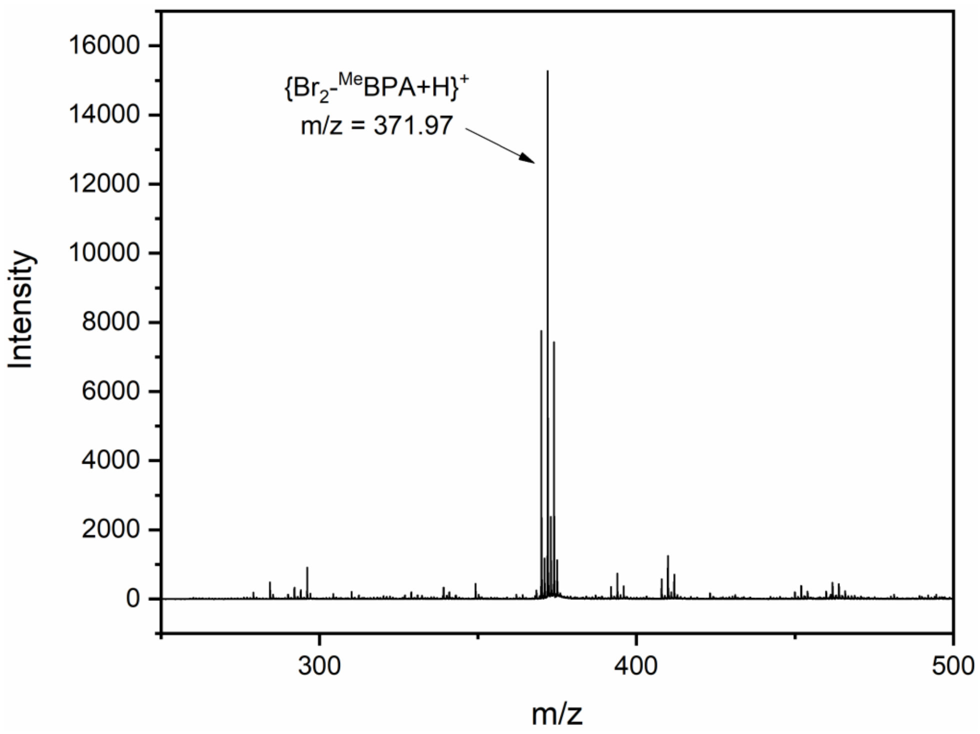

Parent ion fragment $\{Br_2\text{-}^{Me}BPA+H\}^+$:

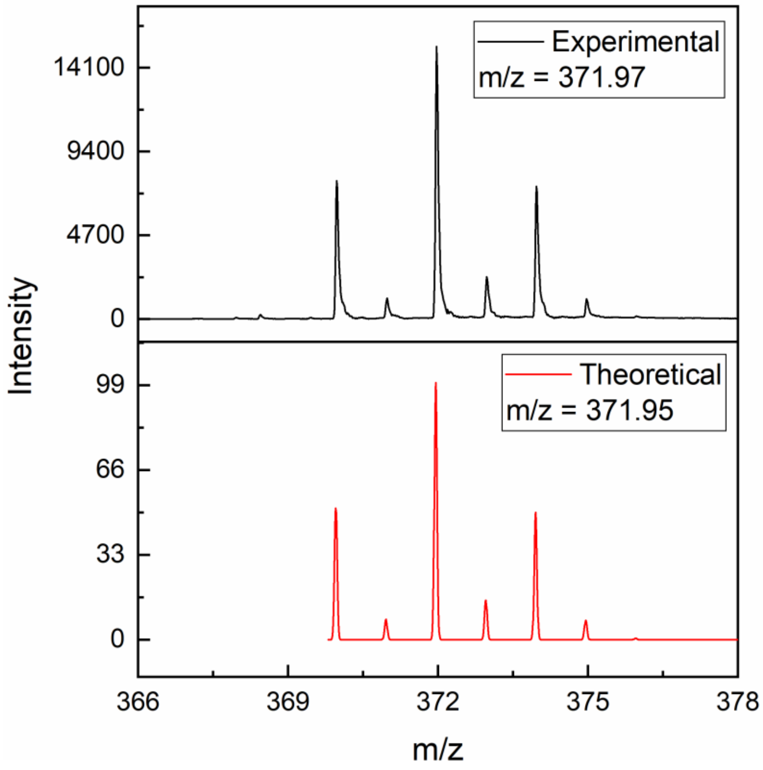

Figure S6. ESI-MS data for $Br_2\text{-}^{Me}BPA$, recorded in chloroform solution, plus expansion and simulation of the parent ion.

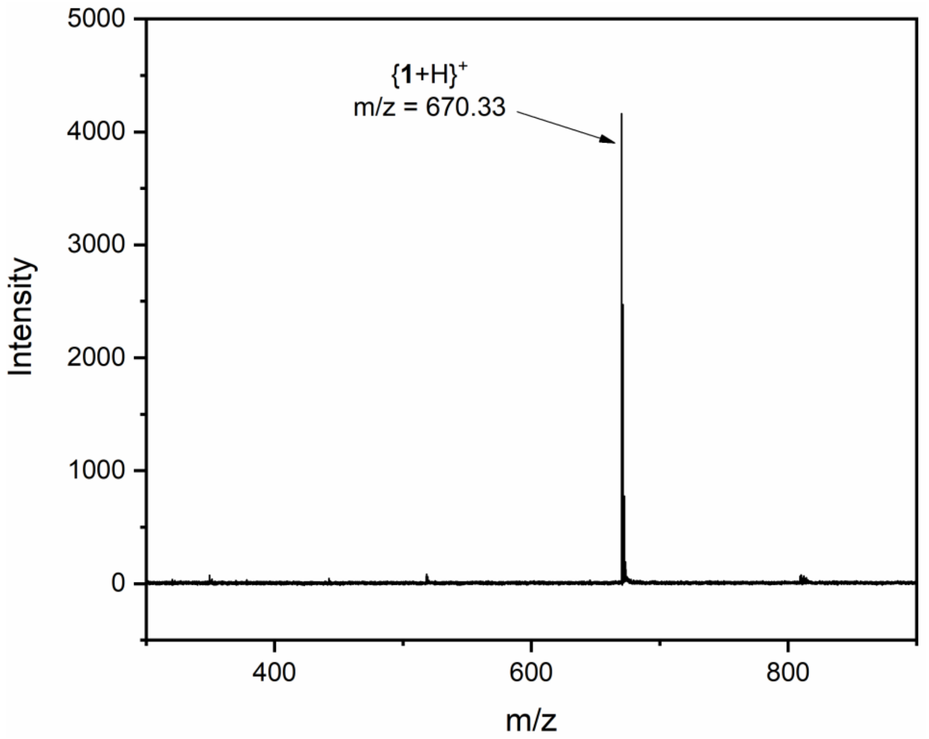

Parent ion fragment {**1**+H}$^{+}$:

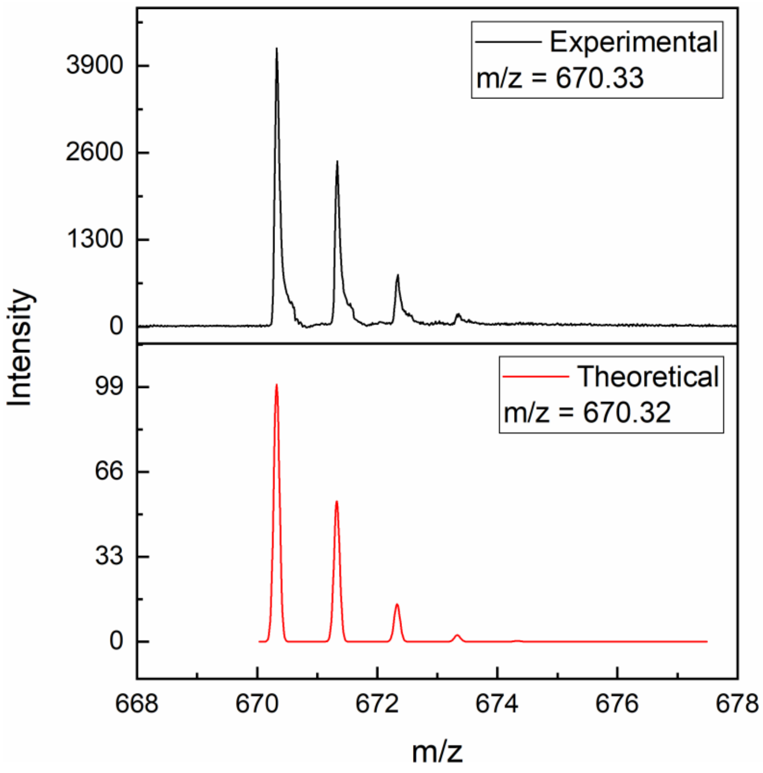

Figure S7. ESI-MS data for **1**, recorded in chloroform solution, plus expansion and simulation of the parent ion.

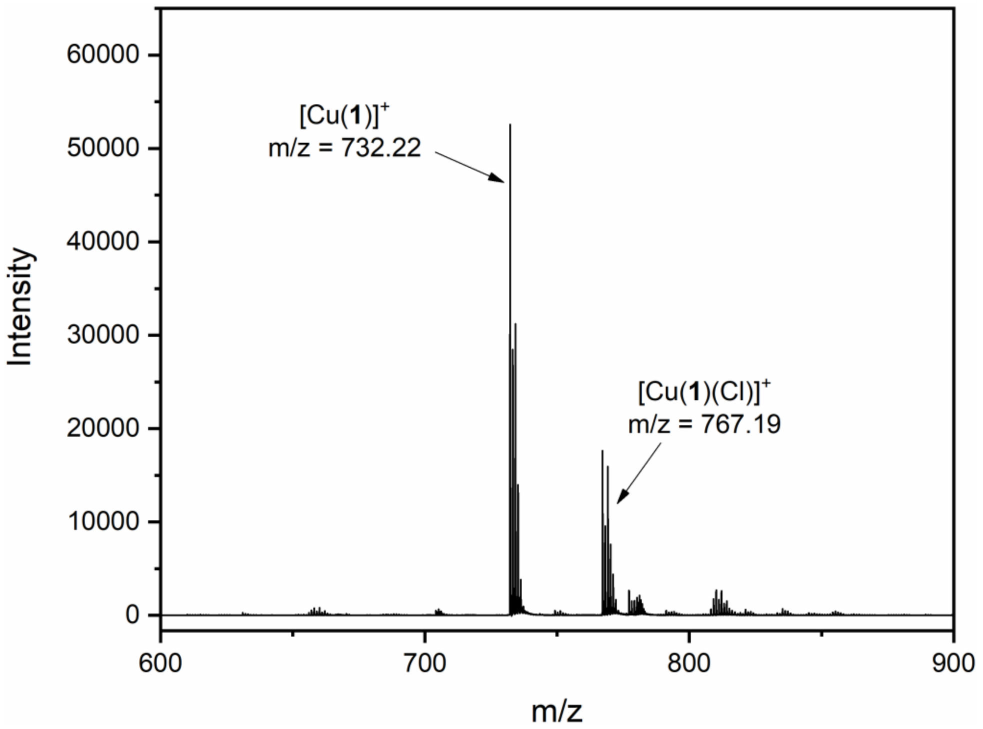

Prominent ion fragments $[Cu(\mathbf{1})]^+$ and $[Cu(\mathbf{1})(Cl)]^+$:

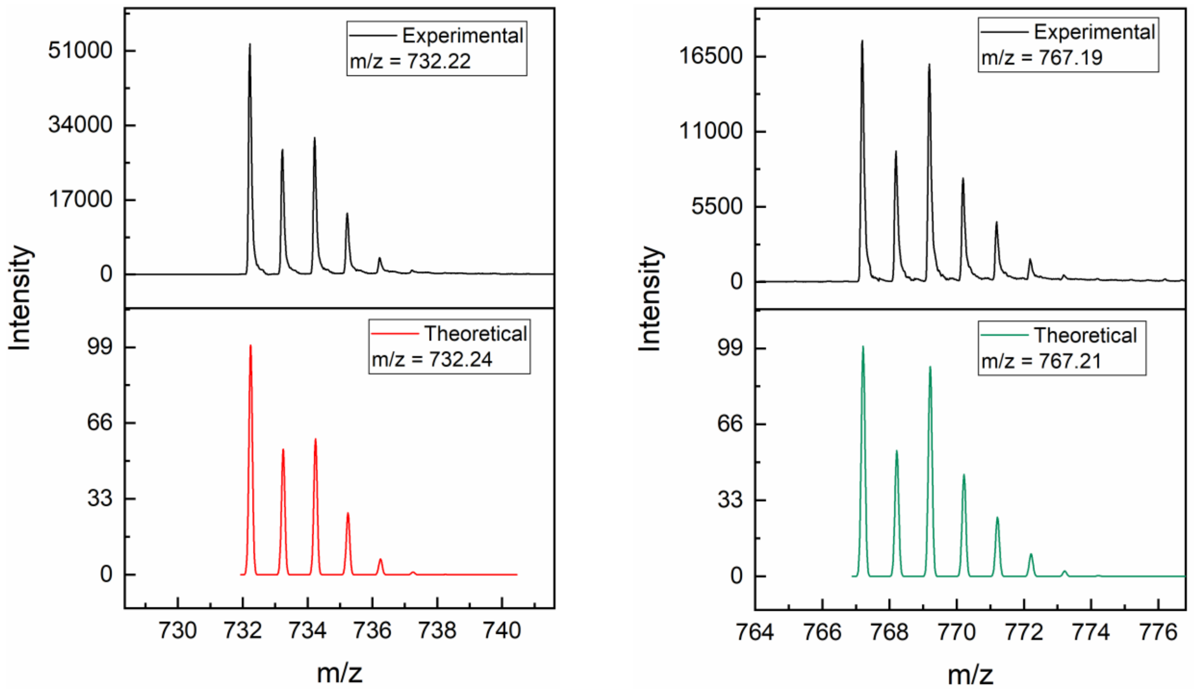

Figure S8. ESI-MS data for $[Cu^{I}(\mathbf{1})(NCMe)][B(C_6F_5)_4]$, recorded in THF solution, plus expansion and simulation of the most prominent ions. The presence of the ion fragment at *m/z* = 767.21, which corresponds to $[Cu^{II}(\mathbf{1})(Cl)]^+$, can be attributed to adventitious chloride ions.

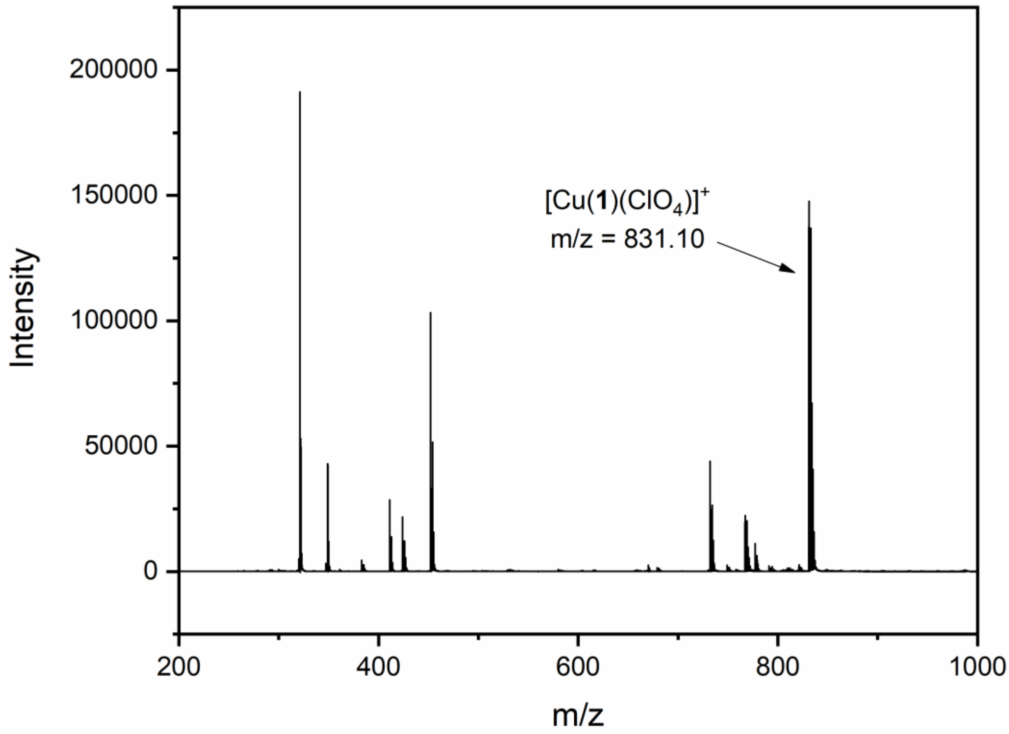

Prominent ion fragment [Cu(**1**)(ClO_4)]$^+$:

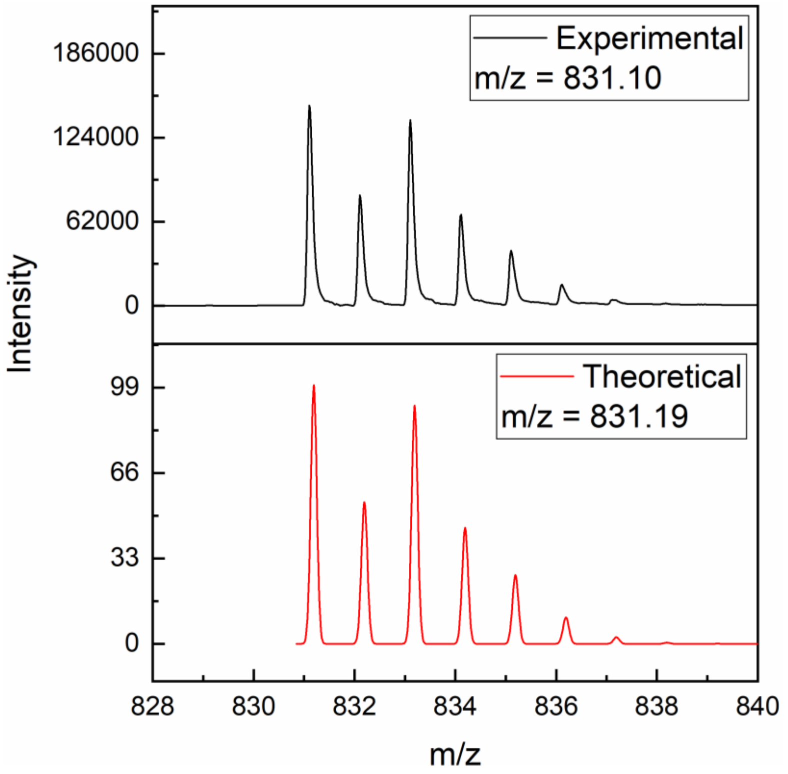

Figure S9. ESI-MS data for [Cu^{II}(**1**)(NCMe)][ClO_4], recorded in THF solution, plus expansion and simulation of the most prominent ion.

X-Ray Crystallography

X-ray crystallographic data was collected using a Bruker APEX II diffractometer equipped with a graphite-monochromated Mo-Kα source (λ = 0.71073 Å) and a CCD area detector (Bruker X8 CCD diffractometer). Data reduction and absorption corrections were performed with the SAINT and SADABS software packages, respectively.[3] Using the Bruker SHELXTL software package,[4-5] the structures were solved by direct methods and refined via full-matrix least squares/difference on F^2. All non-hydrogen atoms were anisotropically refined, after which hydrogen atoms were placed at calculated positions and refined as riding atoms with isotropic displacement parameters.

Table S1. Crystallographic and structure refinement data for the complexes $[Cu^{I}(\mathbf{1})(NCMe)](SbF_6)$ and $[Cu^{II}(\mathbf{1})(NCMe)(OH_2)](ClO_4)_2\cdot MeCN$.

	**$[Cu^{I}(1)(NCMe)](SbF_6)$**	**$[Cu^{II}(1)(NCMe)(OH_2)](ClO_4)_2\cdot MeCN$**
Appearance	Colorless block-like	Blue block-like
Formula	$C_{51}H_{42}CuF_6N_4Sb$	$C_{53}H_{47}Cl_2CuN_5O_9$
FW/ g mol^{-1}	1010.17	1032.39
Cryst. Syst.	triclinic	monoclinic
Space Group	P -1	P 1 21 1
Size/ mm	0.130 · 0.200 · 0.200	0.200 · 0.220 · 0.240
T/ K	100(2)	100(2)
Wavelength/ Å	0.71073	0.71073
a/ Å	12.5313(6)	12.0679(13)
b/ Å	13.6363(7)	14.684(2)
c/ Å	13.6821(7)	13.8465(15)
α / °	95.6584(18)	90
β / °	99.447(2)	95.969(9)
γ / °	101.2000(17)	90
V/ Å^3	2241.7(2)	2440.4(5)
Z	2	2
d_{calc} / g cm^{-3}	1.497	1.405
μ / mm^{-1}	1.142	0.620
Refl. Collected	54487	4447
Goodness of Fit	1.024	0.997
$R; I>2\sigma(I)$	0.0524	0.1069
Largest diff. peak and hole, eÅ^{-3}	1.562 / -1.428	1.132 / -0.732
R.M.S. deviation from mean/ eÅ^{-3}	0.127	0.137

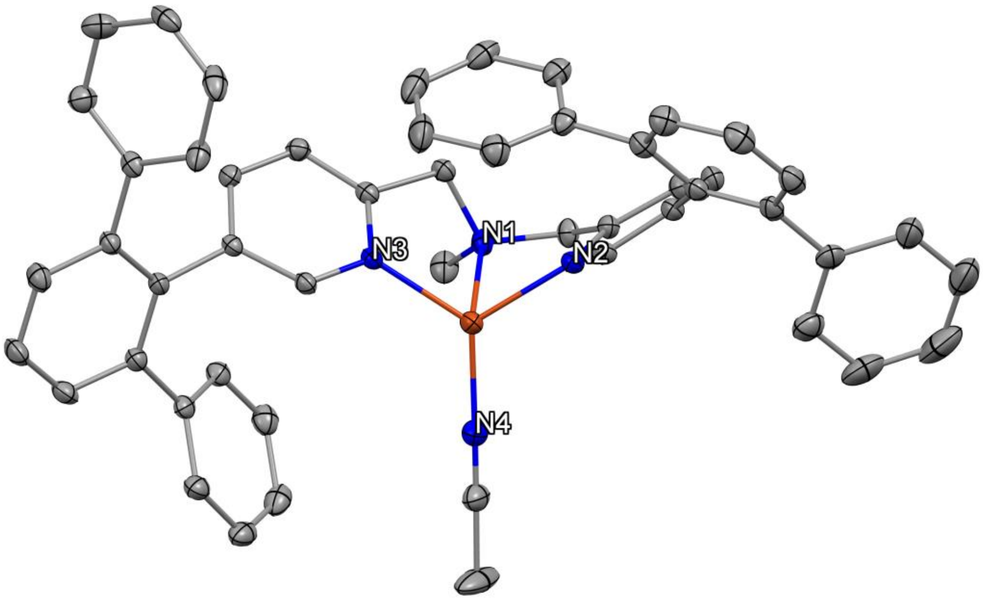

Figure S10. X-ray crystal structure of [Cu^{I}(**1**)(NCMe)](SbF_6), depicted using 50% probability thermal ellipsoids. Hydrogen atoms and counteranions have been omitted for clarity. Gray, orange, and blue spheroids correspond to carbon, copper, and nitrogen atoms, respectively.

Table S2. Selected bond lengths (Å) and bond angles (°) of [Cu^{I}(**1**)(NCMe)](SbF_6).

**Bond Lengths (Å)**		**Bond Angles (°)**	
Cu_1-N_1	2.206(2)	N_1-Cu_1-N_2	79.49(8)
Cu_1-N_2	2.049(2)	N_1-Cu_1-N_3	81.42(8)
Cu_1-N_3	2.020(2)	N_1- Cu_1-N_4	116.64(9)
Cu_1-N_4	1.891(2)	N_2-Cu_1-N_3	113.18(8)
		N_2-Cu_1-N_4	120.56(9)
		N_3-Cu_1-N_4	125.29(9)

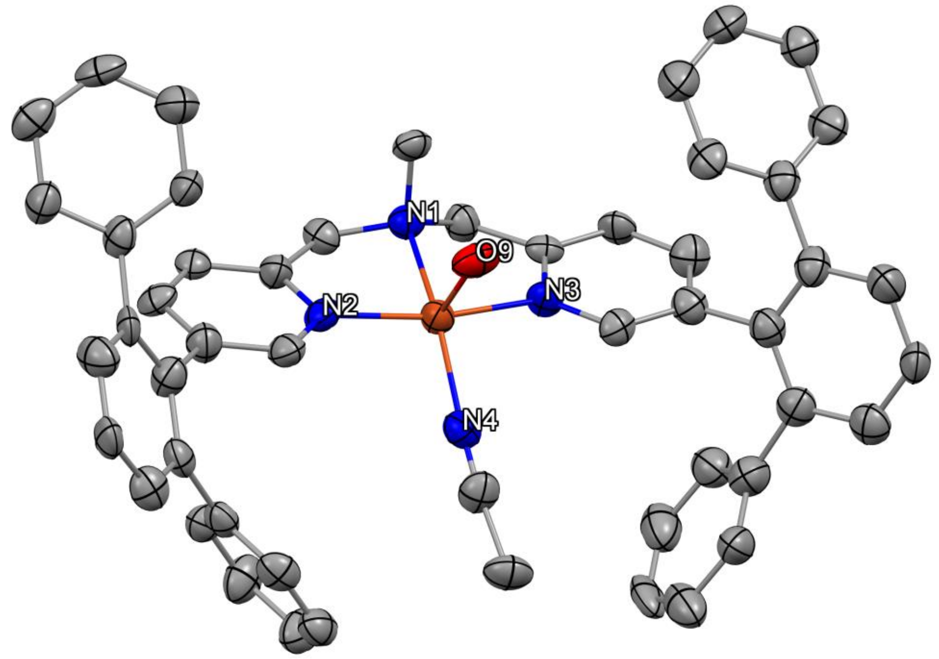

Figure S11. X-ray crystal structure of [Cu^{II}(**1**)(NCMe)(OH_2)](ClO_4)$_2$·MeCN, depicted using 50% probability thermal ellipsoids. Hydrogen atoms, solvent molecules, and counteranions have been omitted for clarity. Gray, orange, blue, and red spheroids correspond to carbon, copper, nitrogen, and oxygen atoms, respectively.

Table S3. Selected bond lengths (Å) and bond angles (°) of [Cu^{II}(**1**)(NCMe)(OH_2)](ClO_4)$_2$·MeCN.

**Bond Lengths (Å)**		**Bond Angles (°)**	
Cu_1-N_1	2.016(13)	N_1-Cu_1-N_2	82.4(4)
Cu_1-N_2	1.963(6)	N_1-Cu_1-N_3	83.9(4)
Cu_1-N_3	1.952(6)	N_1- Cu_1-N_4	163.0(6)
Cu_1-N_4	2.015(13)	N_1- Cu_1-O_9	104.0(5)
Cu_1-O_9	2.273(13)	N_2-Cu_1-N_3	166.2(3)
		N_2-Cu_1-N_4	98.0(4)
		N_2-Cu_1-O_9	93.6(4)
		N_3-Cu_1-N_4	94.7(4)
		N_3-Cu_1-O_9	91.3(4)
		N_4-Cu_1-O_9	92.9(4)

Cyclic Voltammetry

Cyclic voltammograms were recorded using an Autolab PGSTAT204 potentiostat, with a standard three electrode set-up comprised of a glassy carbon working electrode, platinum wire counter electrode, and a silver reference elecrode. The latter was composed of a silver wire inserted into a glass housing stoppered at one end by a porous vycor tip and filled by a 0.1 M acetonitrile solution of $[Bu_4N]PF_6$. All measurements were performed using acetonitrile solutions of $[Cu^{II}(\mathbf{1})(NCMe)(OH_2)](ClO_4)_2 \cdot MeCN$ containing 0.1 M $[Bu_4N]PF_6$ electrolyte, at room temperature, in an argon filled glovebox. Ferrocene was used as internal standard and all data is referenced against the ferrocenium/ferrocene redox couple.

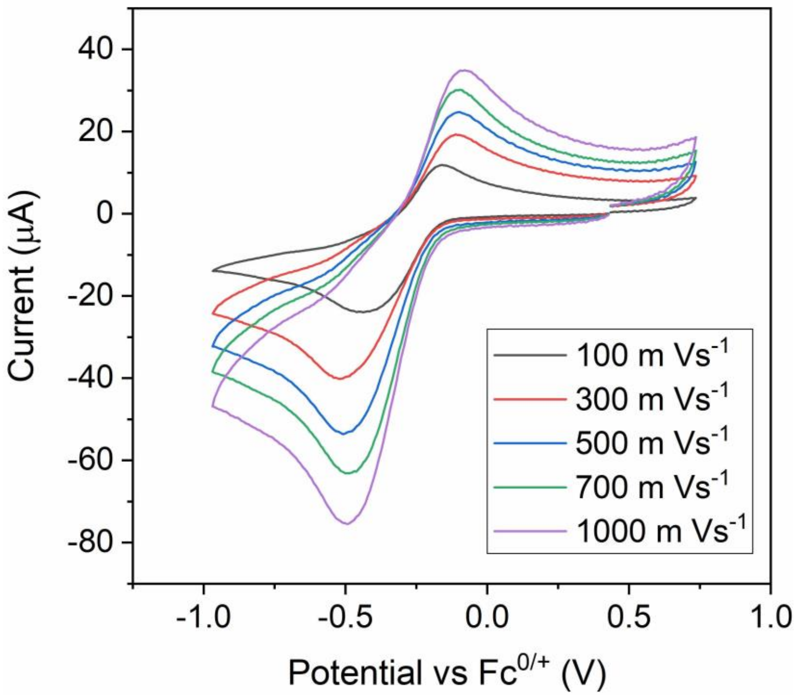

Figure S12. Cyclic voltammogram of $[Cu^{II}(\mathbf{1})(NCMe)(OH_2)](ClO_4)_2$, recorded at various scan rates.

Resonance Raman Spectroscopy

Resonance Raman spectra were recorded using a TriVista 555 triple monochromator equipped with a liquid nitrogen-cooled Roper Scientific 400BR Excelon CCD camera. Excitation was provided by a Millennia – Matisse – Wavetrain triple laser system at 407 nm. The Raman light was collected in a 180° backscattering geometry using an off-axis parabolic mirror, which also minimizes spherical aberrations. The Raman light was focused onto the entrance slit of the Trivista system with a 100 mm diameter f/4 lens. The scattered light was dispersed with gratings of 900 mm^{-1}, 900 mm^{-1} and 1800 mm^{-1} at the three different stages, respectively, giving rise to a spectral resolution at the CCD camera of about 0.8 cm^{-1}.

Samples were prepared directly in EPR tubes by bubbling natural abundance O_2 or high purity $^{18}O_2$ gas through precooled (at -90°C using an ethanol/liquid N_2 bath) 5 mM solutions of [Cu^{I}(**1**)(NCMe)][B(C_6F_5)$_4$] in THF-d$_8$, after which they were frozen using liquid nitrogen. The solutions of copper(I) precursor were prepared inside an Ar filled glovebox and pipetted into quartz EPR tubes, which were sealed with rubber septa prior to removal. During measurement, the samples were kept frozen using a liquid nitrogen-cooled EPR quartz finger.

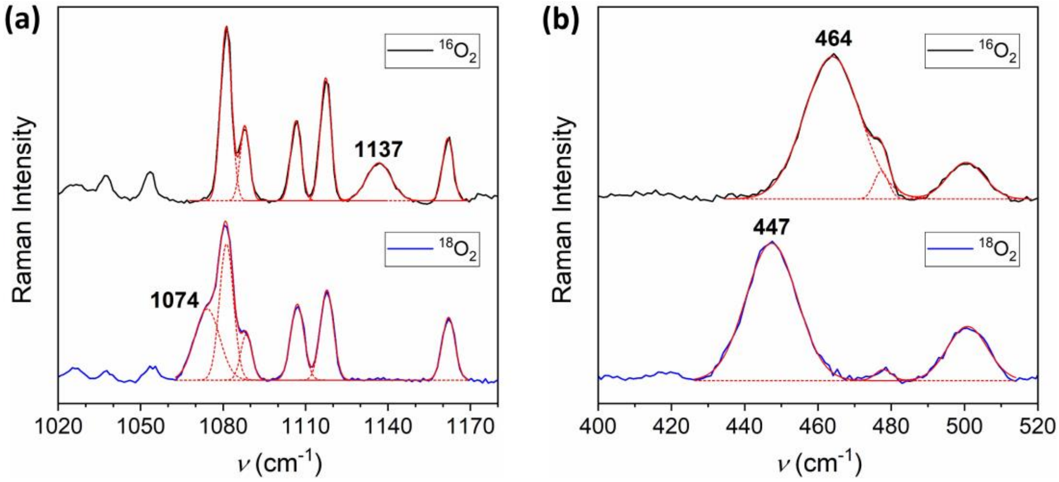

Figure S13. Expansions of the **(a)** ν(O-O) and **(b)** ν(Cu-O) containing regions of the resonance Raman spectra of [Cu^{II}(η^1-$^{16}O_2^{\bullet-}$)(**1**)]$^+$ and [Cu^{II}(η^1-$^{18}O_2^{\bullet-}$)(**1**)]$^+$ (black and blue lines, respectively). Simulations of the spectra and the Gaussians that comprise them are depicted using solid and dashed red lines, respectively.

XAS Measurements

Measurements were performed on solution samples frozen in Delrin sample holders possessing measurement windows sealed with 38 micron thick Kapton tape. Samples of the superoxocopper(II) complexes $[Cu^{II}(\eta^1\text{-}O_2^{\bullet-})(\mathbf{1})]^+$ and $[Cu^{II}(\eta^1\text{-}O_2^{\bullet-})(\mathbf{2})]^+$ were generated by oxygenation of 5 mM solutions of the corresponding copper(I) starting compounds $[Cu^{I}(\mathbf{1})(NCMe)][B(C_6F_5)_4]$ and $[Cu^{I}(\mathbf{2})][B(C_6F_5)_4]$ in THF, at $\leq$ -90 °C. For comparison, data was also recorded for 20 mM acetonitrile solutions of the aforementioned copper(I) starting complexes and the copper(II) salts $[Cu^{II}(\mathbf{1})(NCMe)(OH_2)](ClO_4)_2$ and $[Cu^{II}(\mathbf{2})(NCMe)](ClO_4)_2$.

All HERFD (High Energy Resolution Fluorescence Detected) XAS spectra were measured at the PIPOXS beamline at CHESS, operating at 100 mA at 6 GeV. The beam size was 150 (v) by 450 (h) µm. A Si(311) monochromator was used to scan from 8900 – 9420 eV, with 5 eV steps for 8900 – 8970 eV, 0.2 eV steps for 8970 – 9020 eV, 1 eV steps for 9020 – 9120 eV and 3 eV steps for 9120 – 9420 eV. Incident energy was calibrated to a first inflection point of 8980.3 eV using a copper foil between the second and third ion chambers. Four Si(444) crystals with an 850 mm radius of curvature were used as analyzer crystals, in combination with a DAVES Pilatus 100K spectrometer.[6] The spectrometer was set to the maximum energy for the Kα mainline of each sample. Samples were cooled to 15 K using a He cryostat, and beam flux was attenuated by insertion of aluminium foil, as needed, to mitigate sample damage. Damage studies were performed on each sample by repeatedly scanning on a single spot and observing spectral changes to edge and pre-edge features. All data are the averaged result of multiple scans on fresh spots of each sample. Normalization of XAS experimental spectra were performed using pyMCA.[7]

Kinetics Studies for Reaction With Substrates

In the majority of cases, kinetic studies for reaction of $[Cu^{II}(\eta^1\text{-}O_2^{\bullet-})(\mathbf{1})]^+$ and $[Cu^{II}(\eta^1\text{-}O_2^{\bullet-})(\mathbf{2})]^+$ with substrates were conducted, at the requisite temperatures, by injecting THF solutions of excess substrate (> 10 eq) into preformed solutions of the superoxocopper(II) complexes. The $[Cu^{II}(\eta^1\text{-}O_2^{\bullet-})(\mathbf{1})]^+$ and $[Cu^{II}(\eta^1\text{-}O_2^{\bullet-})(\mathbf{2})]^+$ complexes were prepared by bubbling O_2 into pre-cooled 2 mL portions of 0.5 mM solutions of $[Cu^{I}(\mathbf{1})(NCMe)]^+$ and $[Cu^{I}(\mathbf{2})]^+$, respectively, in THF. Solutions of the copper(I) complexes and substrates were prepared inside an Ar filled glovebox and inserted into 1 cm pathlength quartz cuvettes and samples vials, respectively, which were both stoppered with rubber septa prior to removal. Injection of concentrated solutions of xanthene into pre-cooled solutions of superoxocopper(II) complex at -90°C resulted in precipitation of the substrate. To avoid this, it was necessary to pre-mix the substrate and copper(I) complex at room temperature. Thermal equilibration of this mixture at -90°C (for 5 min) and subsequent oxygenation did not result in formation of precipitate or hinder superoxocopper(II) complex formation.

Reaction of $[Cu^{II}(\eta^1\text{-}O_2^{\bullet-})(\mathbf{1})]^+$ and $[Cu^{II}(\eta^1\text{-}O_2^{\bullet-})(\mathbf{2})]^+$ with the substrates was monitored by measuring decay of their respective UV-Vis spectral bands centred at 404 and 762 nm. For $[Cu^{II}(\eta^1\text{-}O_2^{\bullet-})(\mathbf{2})]^+$, all reactions proceeded with pseudo-first-order behaviour for at least three half-lives. A similar outcome was observed for $[Cu^{II}(\eta^1\text{-}O_2^{\bullet-})(\mathbf{1})]^+$ in reaction with the 4-substituted (X) 2,6-di-tert-butylphenols (X-ArOH, where X = OMe, OMMP, Me, Et, and ^{t}Bu) and 10-methyl-9,10-dihydroacridine. For both complexes, fitting of the aforementioned data provided observed rate constants (k_{obs}). Reaction between $[Cu^{II}(\eta^1\text{-}O_2^{\bullet-})(\mathbf{1})]^+$ and xanthene was comparatively slow and did not strictly adhere to pseudo-first-order kinetics for a full three half-lives. In this case, k_{obs} values were estimated using initial rates, with fitting performed for the first ≤ 35 % of reaction. For all substrates, the k_{obs} values were found to be linearly dependent upon substrate concentration and fitting of this data yielded second order rate constants (k_2). The k_2 values were estimated to have standard errors of 5%, which is significantly larger than the standard errors obtained from fitting the k_{obs} values.

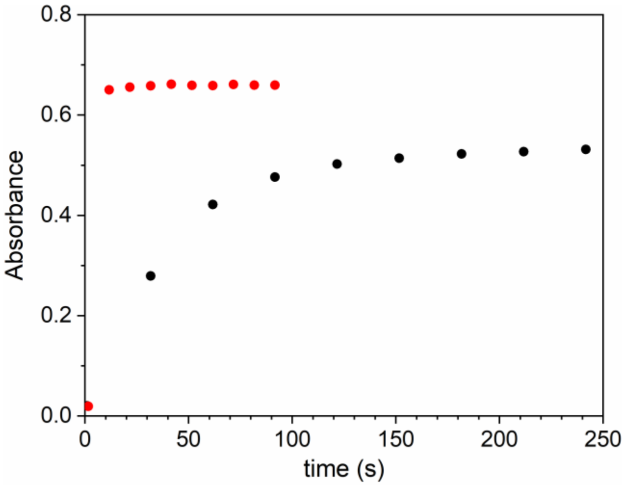

Figure S14. Time traces for oxygenation of [Cu^{I}(**1**)(NCMe)]$^{+}$ and [Cu^{I}(**2**)]$^{+}$ at -90°C (black and red circles, respectively), monitored by UV-Vis spectroscopy at respective wavelengths of 722 and 760 nm.

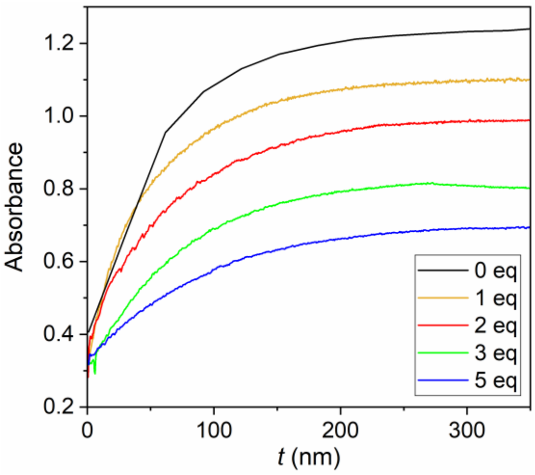

Figure S15. Time traces for oxygenation of 0.5 mM solution of [Cu^{I}(**1**)(NCMe)]$^{+}$ in THF, at -90 °C, with *n* equivalents of acetonitrile added (*n* = 0, black line; 1, yellow line; 2, red line; 3, green line; 5, blue line). Monitored at a wavelength of 404 nm.

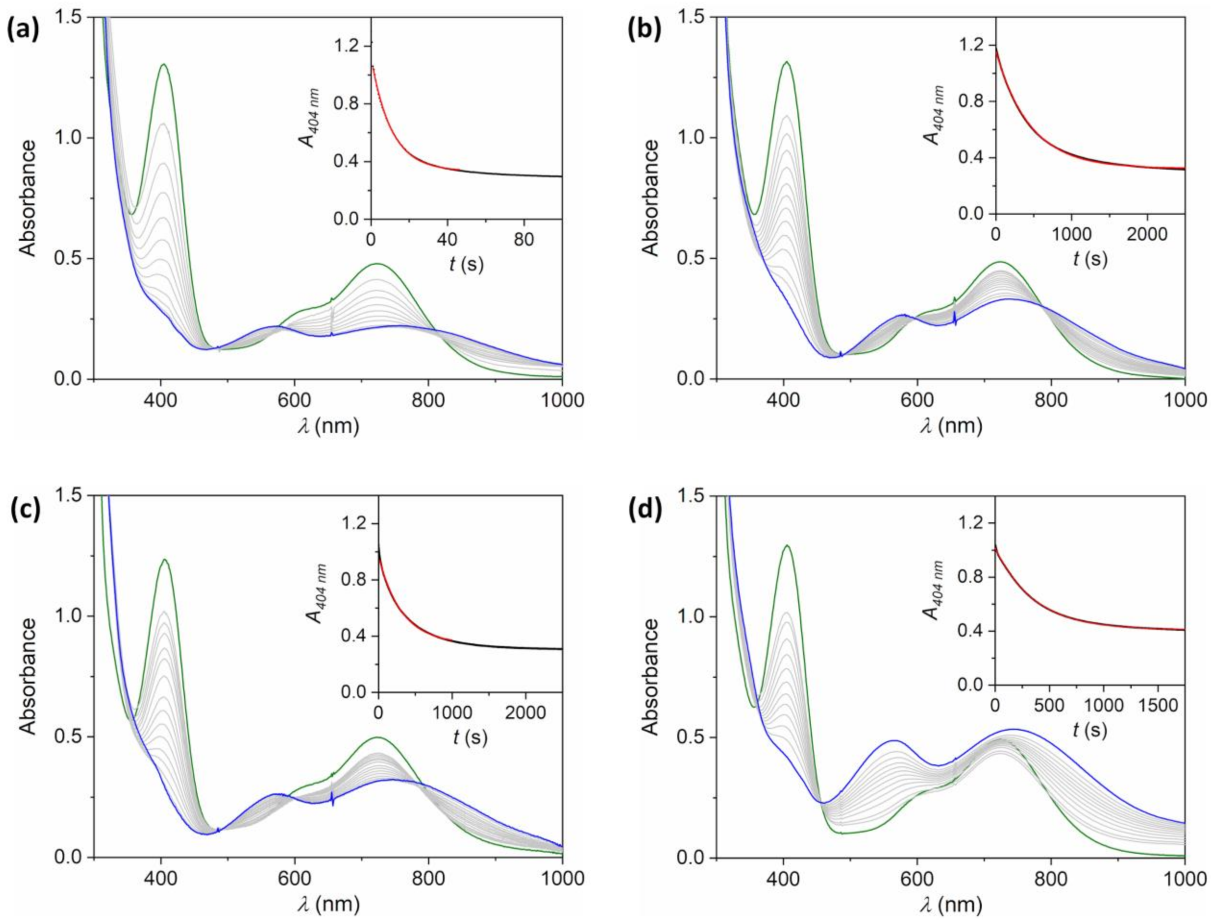

Figure S16. UV-Vis spectral changes accompanying reaction of $[Cu^{II}(\eta^1\text{-}O_2^{\bullet-})(\mathbf{1})]^+$ with the substrates **(a)** MPPO-ArOH, **(b)** Me-ArOH, **(c)** Et-ArOH, and **(d)** ^{t}Bu-ArOH, in THF solution at -90°C. Main: green, blue, and gray lines correspond to $[Cu^{II}(\eta^1\text{-}O_2^{\bullet-})(\mathbf{1})]^+$, the product of reaction, and the spectra recorded in between, respectively. Inset: Absorbance at 404 nm as a function of time and best-fit line (black dots and red lines, respectively).

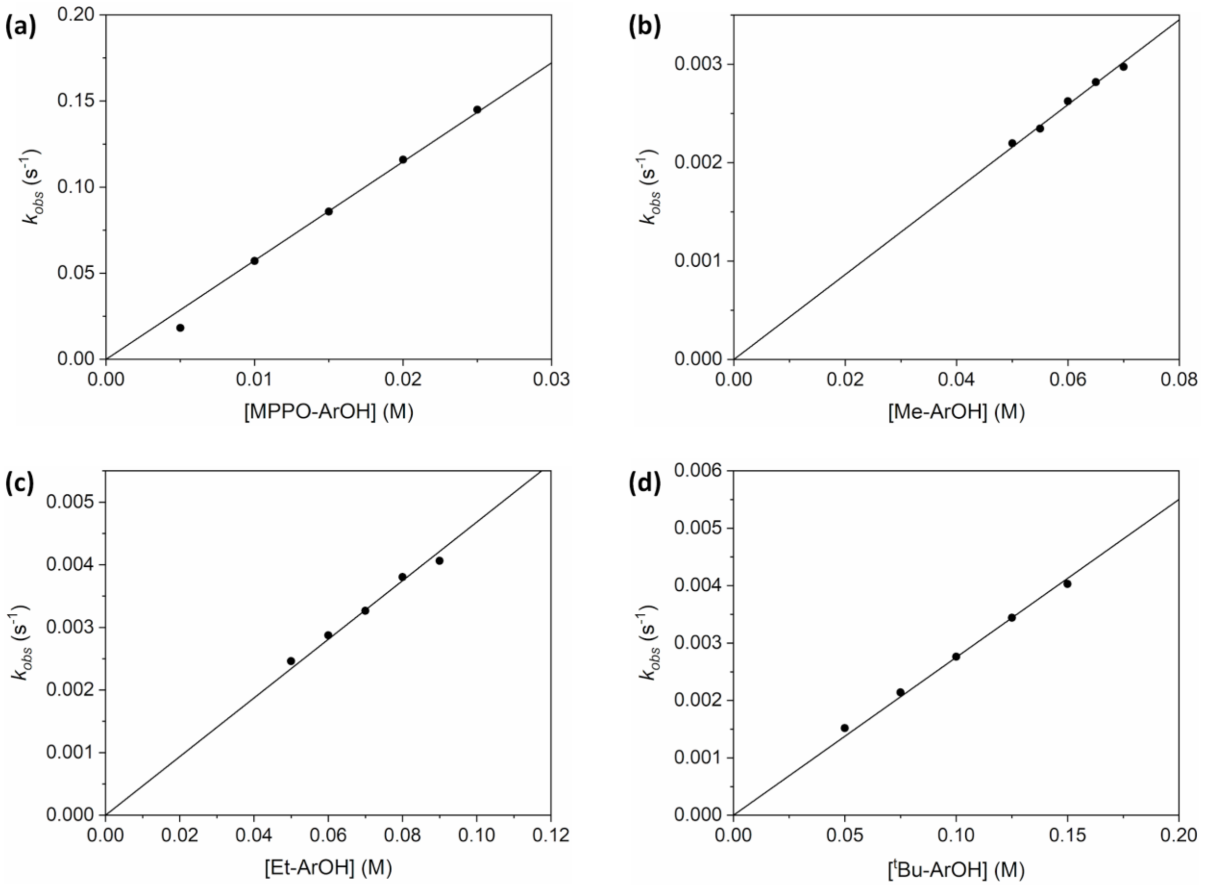

Figure S17. Plots of observed rate constants (k_{obs}, s^{-1}) versus substrate concentration (M), including linear best-fit lines, for reaction of $[Cu^{II}(\eta^1\text{-}O_2^{\bullet-})(\mathbf{1})]^+$ with the substrates **(a)** MPPO-ArOH, **(b)** Me-ArOH, **(c)** Et-ArOH, and **(d)** ^{t}Bu-ArOH, recorded in THF solution at -90°C.

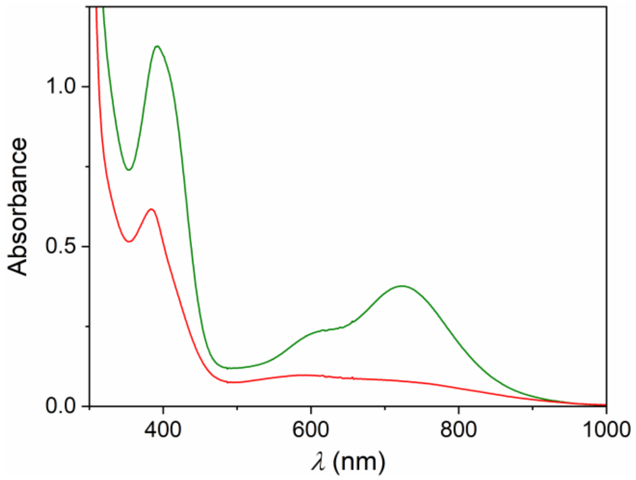

Figure S18. UV-Vis spectrum of $[Cu^{II}(OOH)(\mathbf{1})]^+$ (red line), produced by reaction of $[Cu^{II}(\eta^1\text{-}O_2^{\bullet-})(\mathbf{1})]^+$ (green line) with 10 equiv of TEMPO-H, in THF solution at -90°C.

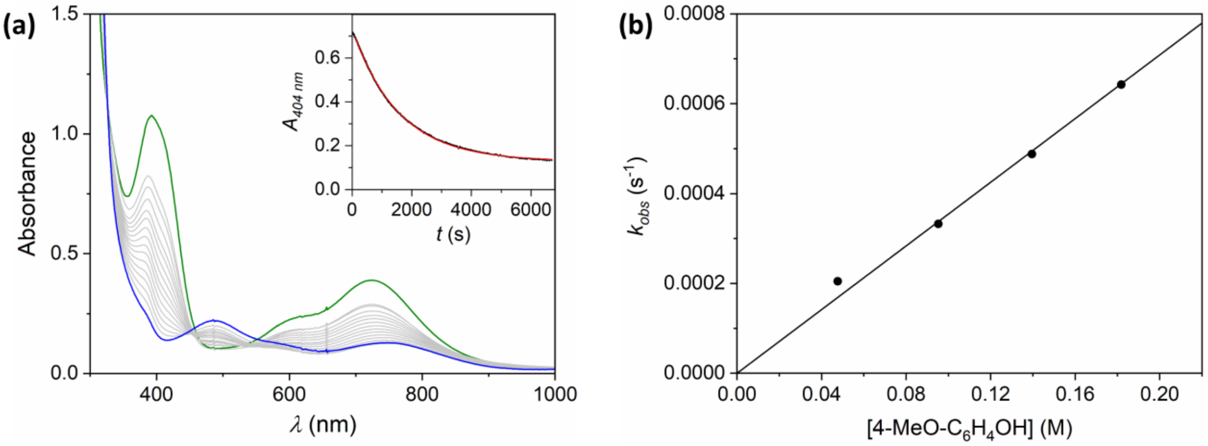

Figure S19. (a) UV-Vis spectral changes accompanying reaction of [Cu^{II}(η^1-$O_2^{\bullet-}$)(**1**)]$^+$ with the substrate 4-methoxyphenol, *p*-MeO-C_6H_4OH, recorded in THF solution at -90°C. Main: green, blue and gray lines correspond to [Cu^{II}(η^1-$O_2^{\bullet-}$)(**1**)]$^+$, the product of reaction, and the spectra recorded in between, respectively. Inset: Absorbance at 404 nm as a function of time and best-fit line (black dots and red line, respectively). **(b)** A plot of observed rate constant (k_{obs}, s^{-1}) versus substrate concentration (M), including best-fit line, for reaction of [Cu^{II}(η^1-$O_2^{\bullet-}$)(**1**)]$^+$ with *p*-MeO-C_6H_4OH, recorded in THF solution at -90°C.

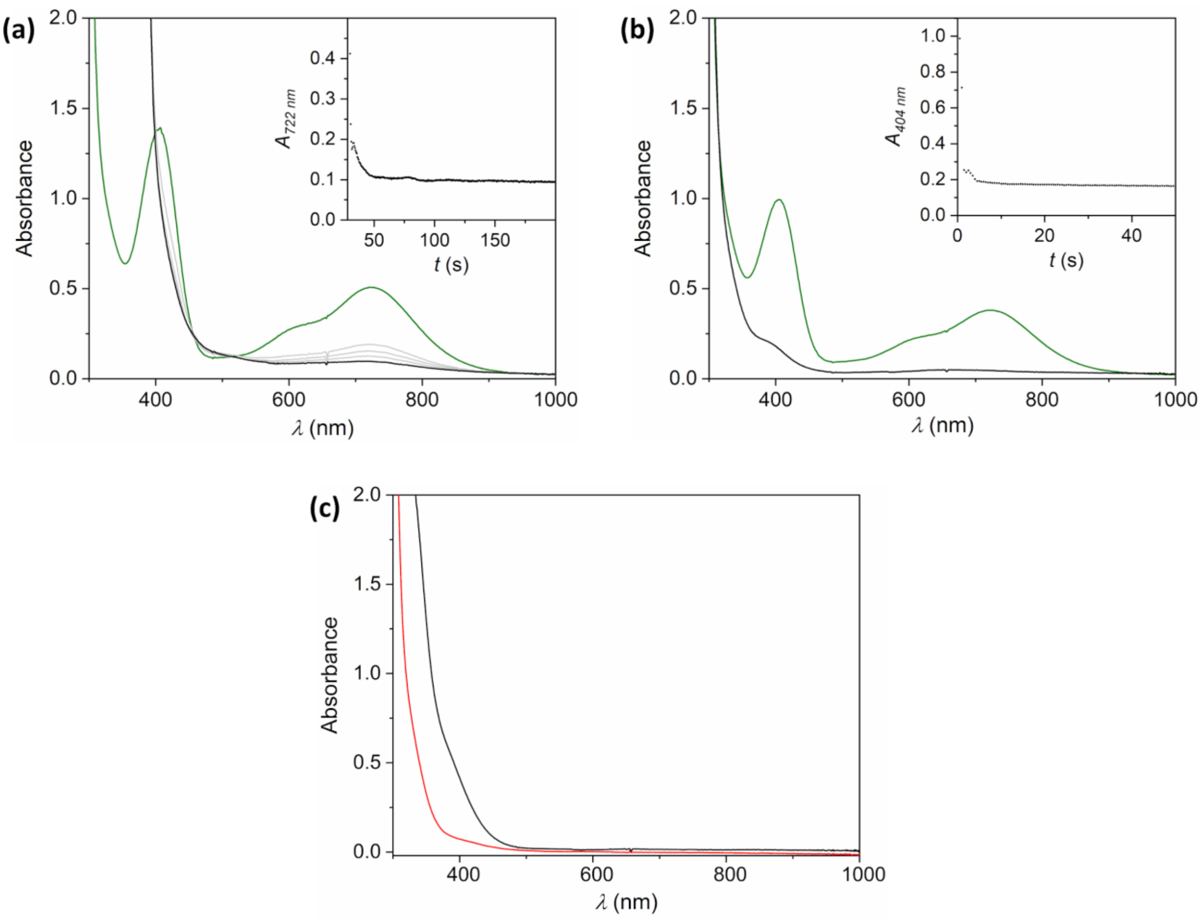

Figure S20. UV-Vis spectral changes accompanying reaction of $[Cu^{II}(\eta^1\text{-}O_2^{\bullet-})(\mathbf{1})]^+$ with the substrates **(a)** BNAH and **(b)** 1,4-CHD, in THF solution at -90°C. Main: green, black and gray lines correspond to $[Cu^{II}(\eta^1\text{-}O_2^{\bullet-})(\mathbf{1})]^+$, the product of reaction, and the spectra recorded during the course of reaction with the substrate, respectively. Inset: Absorbance at 722 and 404 nm, respectively, as a function of time. **(c)** UV-Vis spectral changes resulting from addition of 10 equiv of 1,4-CHD to $[Cu^{I}(\mathbf{1})(NCMe)]^+$ (black and red lines corresponding to the initial and final spectra, respectively).

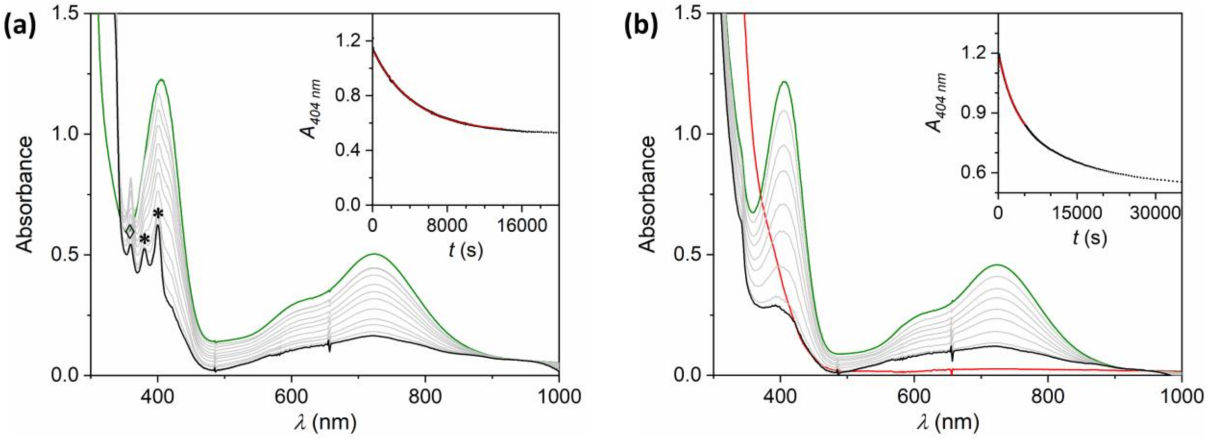

Figure S21. UV-Vis spectral changes accompanying reaction of $[Cu^{II}(\eta^1\text{-}O_2^{\bullet-})(\mathbf{1})]^+$ with the substrates **(a)** $^{Me}AcrH_2$ and **(b)** xanthene, in THF solution at -90°C. Main: red, green, black and gray lines correspond to $[Cu^{I}(\mathbf{1})(NCMe)]^+$ (only present in **(b)**), $[Cu^{II}(\eta^1\text{-}O_2^{\bullet-})(\mathbf{1})]^+$, the product of reaction, and the spectra recorded during the course of reaction with the substrate, respectively. * = *N*-methyl-9-acridone and ◊ = $^{Me}AcrH^+$. Inset: Absorbance at 404 nm as a function of time and best-fit line (black dots and red line, respectively).

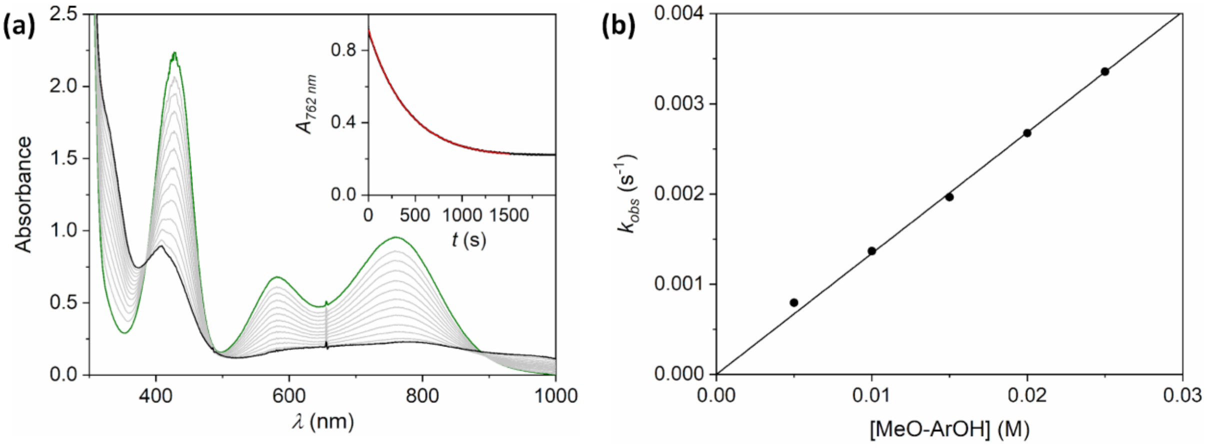

Figure S22. (a) UV-Vis spectral changes accompanying reaction of $[Cu^{II}(\eta^1\text{-}O_2^{\bullet-})(\mathbf{2})]^+$ with the substrates MeO-ArOH, recorded in THF solution at -90°C. Main: green, black and gray lines correspond to $[Cu^{II}(\eta^1\text{-}O_2^{\bullet-})(\mathbf{2})]^+$, the product of reaction, and the spectra recorded in between, respectively. Inset: Absorbance at 762 nm as a function of time and best-fit line (black dots and red line, respectively). **(b)** A plot of observed rate constant (k_{obs}, s^{-1}) versus substrate concentration (M), including best-fit line, for reaction of $[Cu^{II}(\eta^1\text{-}O_2^{\bullet-})(\mathbf{2})]^+$ with the substrate MeO-ArOH, recorded in THF solution at -90°C.

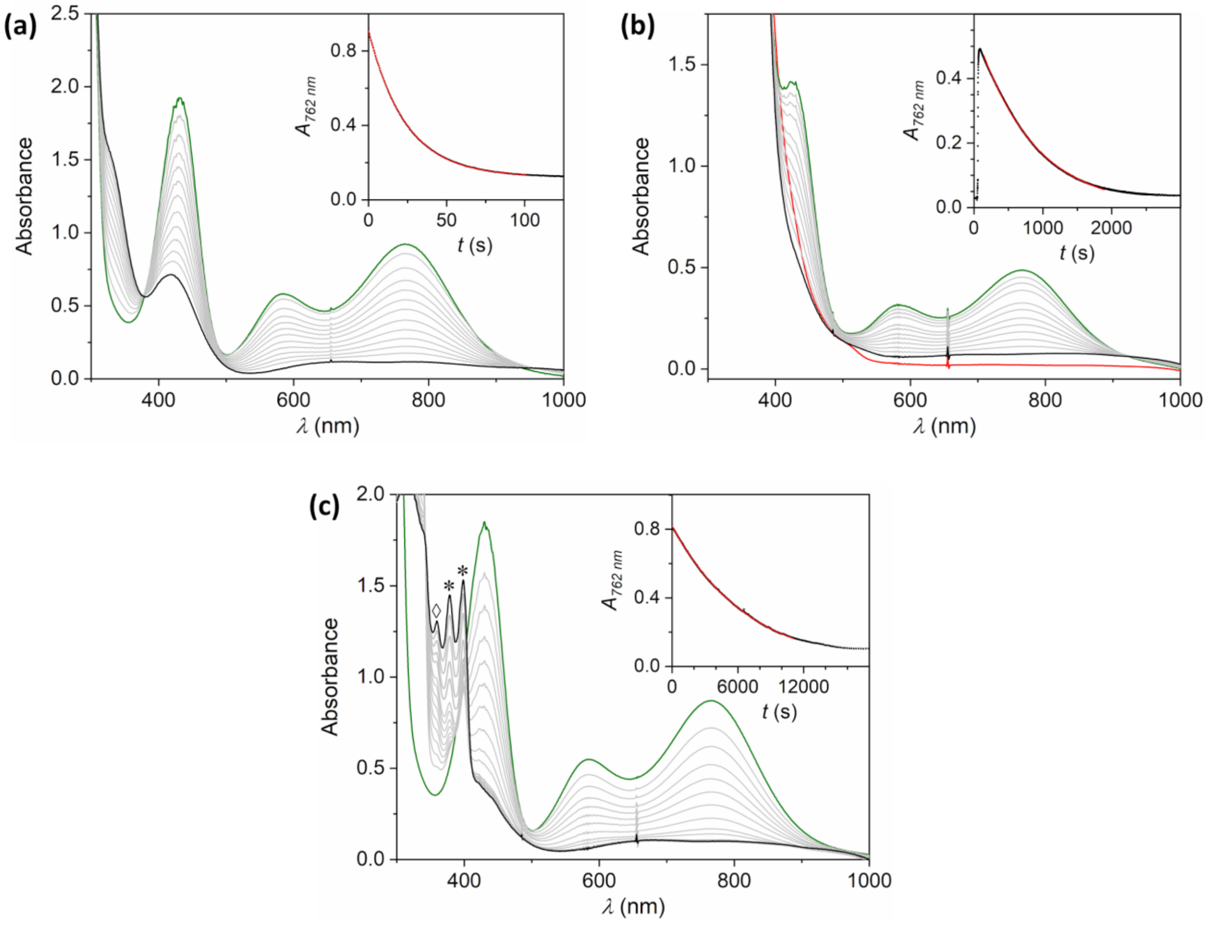

Figure S23. UV-Vis spectral changes accompanying reaction of $[Cu^{II}(\eta^1\text{-}O_2^{\bullet-})(\mathbf{2})]^+$ with the substrates **(a)** MeO-ArOH, **(b)** BNAH, and **(c)** $^{Me}AcrH_2$, recorded in THF solution at -40°C. Main: red, green, black and gray lines correspond to $[Cu^{I}(\mathbf{2})(NCMe)]^+$ (only present in **(b)**), $[Cu^{II}(\eta^1\text{-}O_2^{\bullet-})(\mathbf{2})]^+$, the product of reaction, and the spectra recorded during the course of reaction with the substrate, respectively. ∗ = *N*-methyl-9-acridone and ◊ = $^{Me}AcrH^+$. Inset: Absorbance at 762 nm as a function of time and fit line (black dots and red line, respectively).

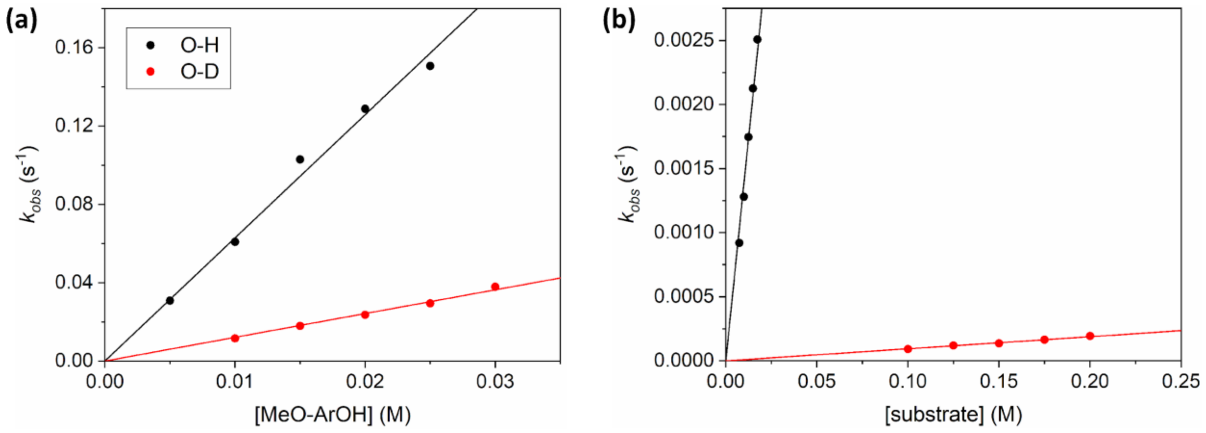

Figure S24. Plots of observed rate constants (k_{obs}, s^{-1}) versus substrate concentration (M), including linear best-fit lines, for reaction of $[Cu^{II}(\eta^1\text{-}O_2^{\bullet-})(\mathbf{2})]^+$ with the substrates **(a)** MeO-ArOH and MeO-ArOD (black and red circles/lines, respectively), and **(b)** BNAH and $^{Me}AcrH_2$ (black and red circles/lines, respectively), recorded in THF solution at -40°C.

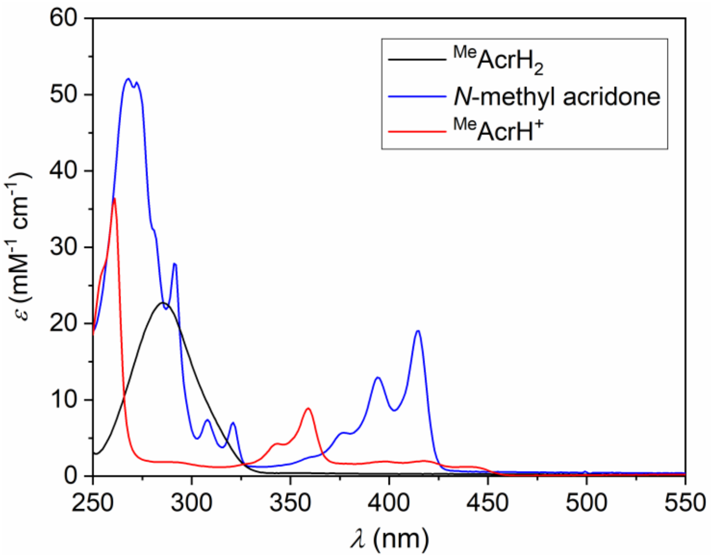

Figure S25. UV-Vis spectra of $^{Me}AcrH_2$, *N*-methylacridone, and $^{Me}AcrH^+$ (black, blue and red lines, respectively), recorded at -90°C in THF solution. The extinction coefficients of $^{Me}AcrH^+$ are likely underestimated due to the poor solubility of the salt used, $[^{Me}AcrH]I$, in THF.

Table S4. Second order rate constants (k_2, M^{-1} s^{-1}) measured for reaction of $[Cu^{II}(\eta^1\text{-}O_2^{\bullet-})(\mathbf{1})]^+$ and $[Cu^{II}(\eta^1\text{-}O_2^{\bullet-})(\mathbf{2})]^+$ with a variety of O-H and C-H bond substrates, in THF solution. Where available, the reported pK_a values, oxidation potentials (E_{ox}, V *vs* Fc^+/Fc^0) and bond dissociation energies (BDEs, kcal mol^{-1}) of the substrates are listed.[a]

$[Cu^{II}(^1\eta\text{-}O_2^{\bullet-})(L)]^+$, L =	Temperature (°C)	Substrate	k_2 (M^{-1} s^{-1})	BDE (X-H) (kcal mol^{-1})	E_{ox} (V *vs* $Fc^{+/0}$)	pK_a
**1**	-90	MeO-ArOH	15(1)	82.0 [d]	0.526	18.2
		MeO-ArOD	2.6(1)		0.585	
		MPPO-ArOH [b]	4.8(2)		0.614	
		Me-ArOH	0.042(2)	82.5 [d]	0.81	17.7
		Et-ArOH	0.044(2)		0.875	17.7
		^{t}Bu-ArOH	0.026(1)	85.2 [d]	0.927	17.8
		p-MeO-C_6H_4OH [d]	0.0035(2)	87.6		19.1
		$^{Me}AcrH_2$ [c]	0.0059(3)	73.0	0.460	
		Xanthene [d]	0.00065(3)	77.9		30.0
**2**	-40	MeO-ArOH	6.3(3)	79.6	0.526	18.2
		MeO-ArOD	1.2(1)		0.585	
		BNAH [c]	0.14(1)	70.7	0.219	
		$^{Me}AcrH_2$ [c]	0.00094(5)	73.0	0.460	

[a] Unless otherwise specified, BDE and pK_a values (in dmso) are taken from ref. 8, and the E_{ox} values (in MeCN) are taken from ref. 9. [b] MPPO = 2-methyl-1-phenylpropan-2-yloxy. [c] E_{ox} values and BDEs (in MeCN) are taken from ref. 10. [d] BDE and/or pKa values (in MeCN) are taken from ref. 11.

UV-Vis Spectra of [Cu^{II}(OAr-X)(1)]$^{+}$

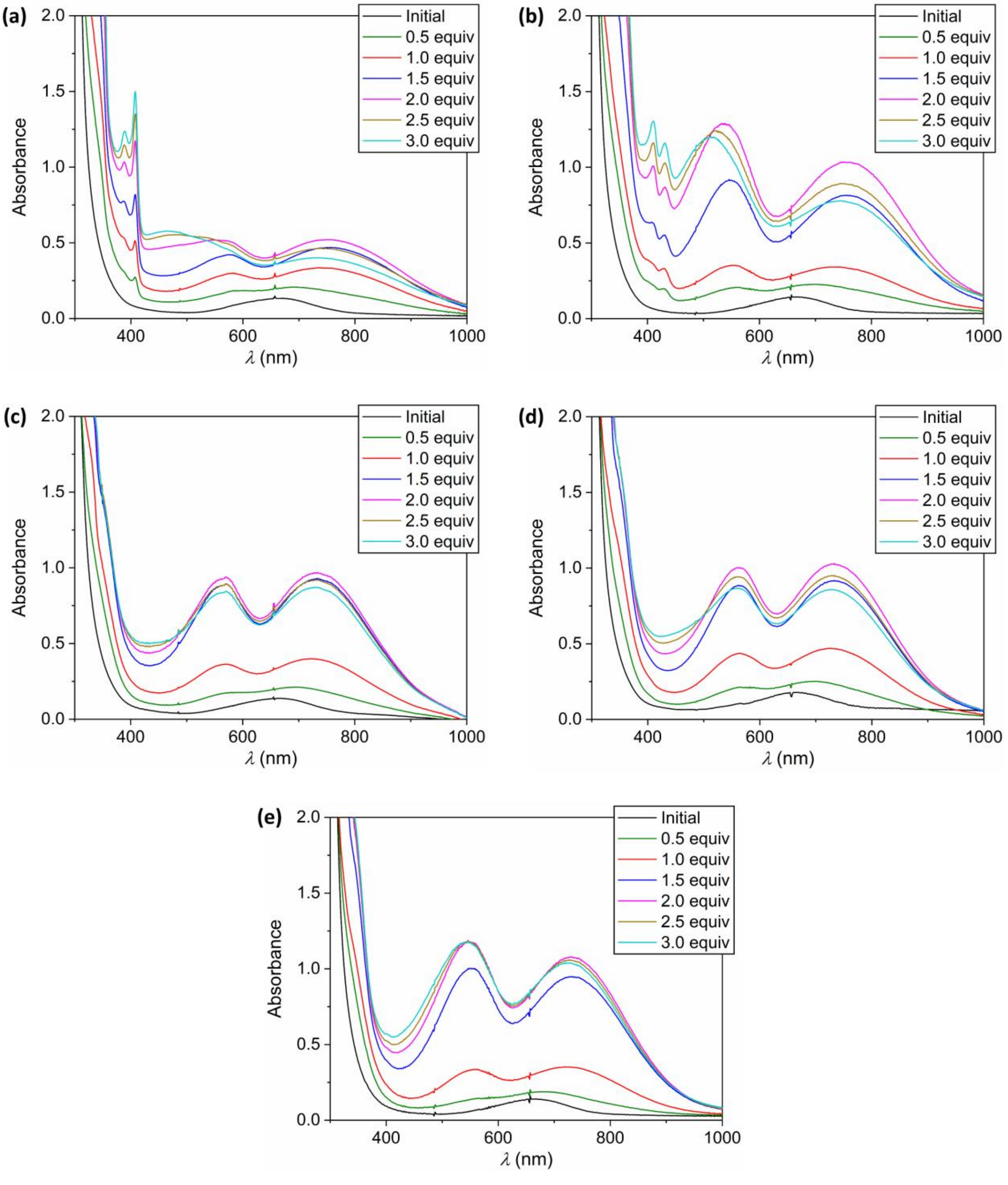

Figure S26. UV-Vis spectra obtained from reaction of 1mM [Cu^{II}(**1**)(NCMe)](ClO_4)$_2$ with *n* equiv of 0.05 mM of **(a)** MeO-ArOK, **(b)** MPPO-ArOK, **(c)** Me-ArOK, **(d)** Et-ArOK, and **(e)** ^{t}Bu-ArOK, both in THF solution, at -90°C. Solutions of the phenolate salts (X-ArOK) were produced by reaction of equimolar amounts of X-ArOH and ^{t}BuOK.

EPR Spectra

Stock solutions of substrates and EPR tubes charged with 0.2 mL of 1.5mM $[Cu^{II}(\eta^1\text{-}O_2^{\bullet -})(\mathbf{1})]^+$ were both prepared inside an argon filled glovebox, and sealed with rubber septa prior to removal. After the EPR tubes were thermally equilibrated at -90°C, in an ethanol/liquid nitrogen bath, dry dioxygen was bubbled through the solutions for 3 mins. 100 μL of substrate solution was layered on top of the superoxocopper(II) solution and the resulting mixture was left undisturbed for 5 mins at -90°C, after which bubbling with argon (for 30 s) was used to mix the layers. The resulting mixture was left to react to completion at -90°C and, subsequently, flash frozen for analysis. A similar procedure, but without dioxygen bubbling, was used for the reaction of $[Cu^{II}(\mathbf{1})(NCMe)](ClO_4)_2$ with 2 equivalents of deprotonated phenols. During reactions of $[Cu^{II}(\eta^1\text{-}O_2^{\bullet -})(\mathbf{1})]^+$ with xanthene and $^{Me}AcrH_2$, precipitation of substrate from solution at low temperatures hindered homogenous mixing. Instead, reaction for these substrates was carried out using the procedures employed for reaction kinetics measurements (i.e., in an UV-Vis cuvette, with cyrostat controlled cooling). Upon completion of reaction, 0.3 mL of the product mixture was withdrawn, injected into an argon filled EPR tube, and flash frozen.

Table S5. Spin Hamiltonian *g*- and *A*-values (10^{-4} cm^{-1}) derived from simulation of the EPR spectra of the products from reaction of $[Cu^{II}(\eta^1\text{-}O_2^{\bullet -})(\mathbf{1})]^+$ with X-ArOH.

X	g_z	$g_{x,y}$	A_z	$A_{x,y}$
MeO	2.240	2.060	182	10
MPPO	2.236	2.056	180	20
Me	2.246	2.060	182	10
Et	2.253	2.062	185	10
^{t}Bu	2.243	2.060	178	14

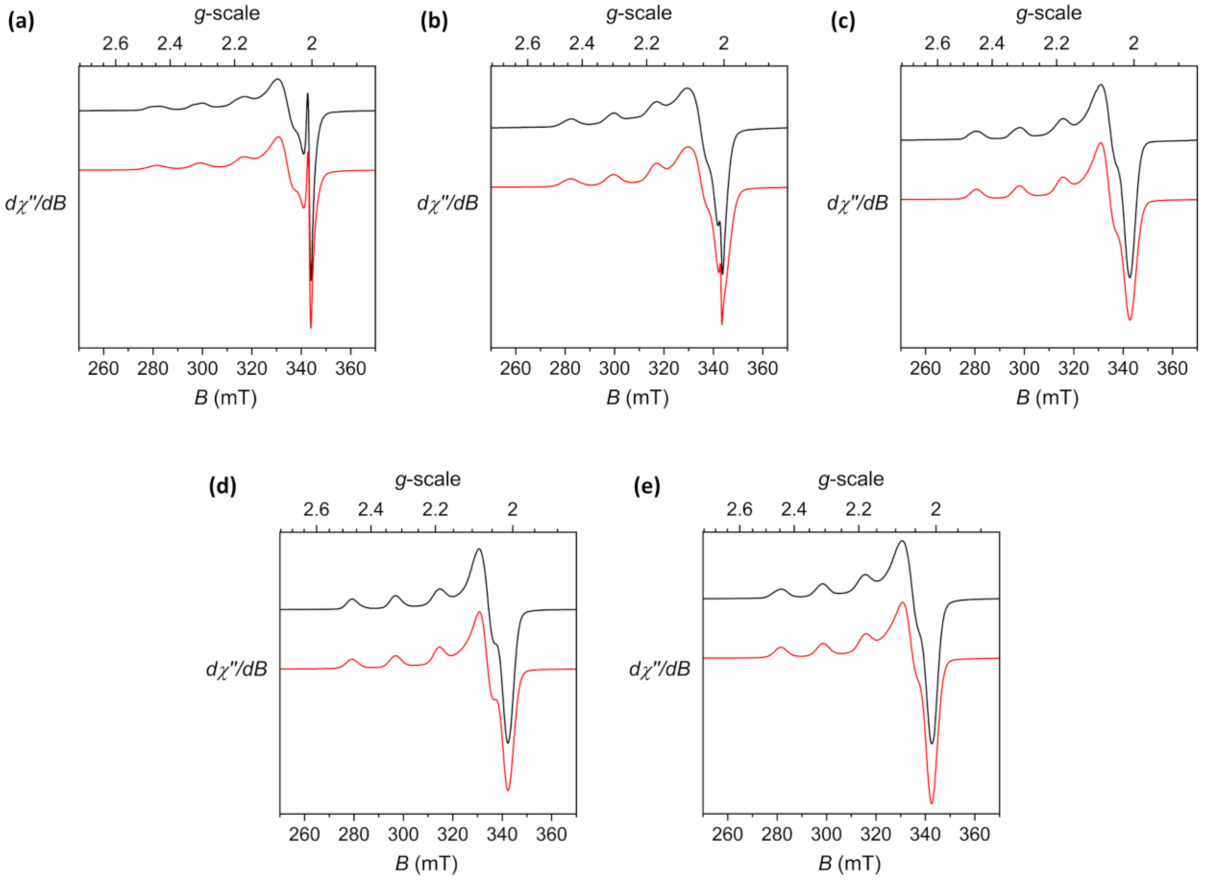

Figure S27. EPR spectra of the product mixtures obtained from reaction of $[Cu^{II}(\eta^1\text{-}O_2^{\bullet-})(\mathbf{1})]^+$ with **(a)** MeO-ArOH, **(b)** MPPO-ArOH, **(c)** Me-ArOH, **(d)** Et-ArOH, and **(e)** ^{t}Bu-ArOH, in THF solution at -90°C. Black and red lines correspond to experimental data and spectral simulations, respectively. In addition to the copper(II)-containing species present, there are also small amounts of organic radical(s) in **(a)** and **(b)**, which are assumed to be aryloxyl radicals (X-ArO$^\bullet$).

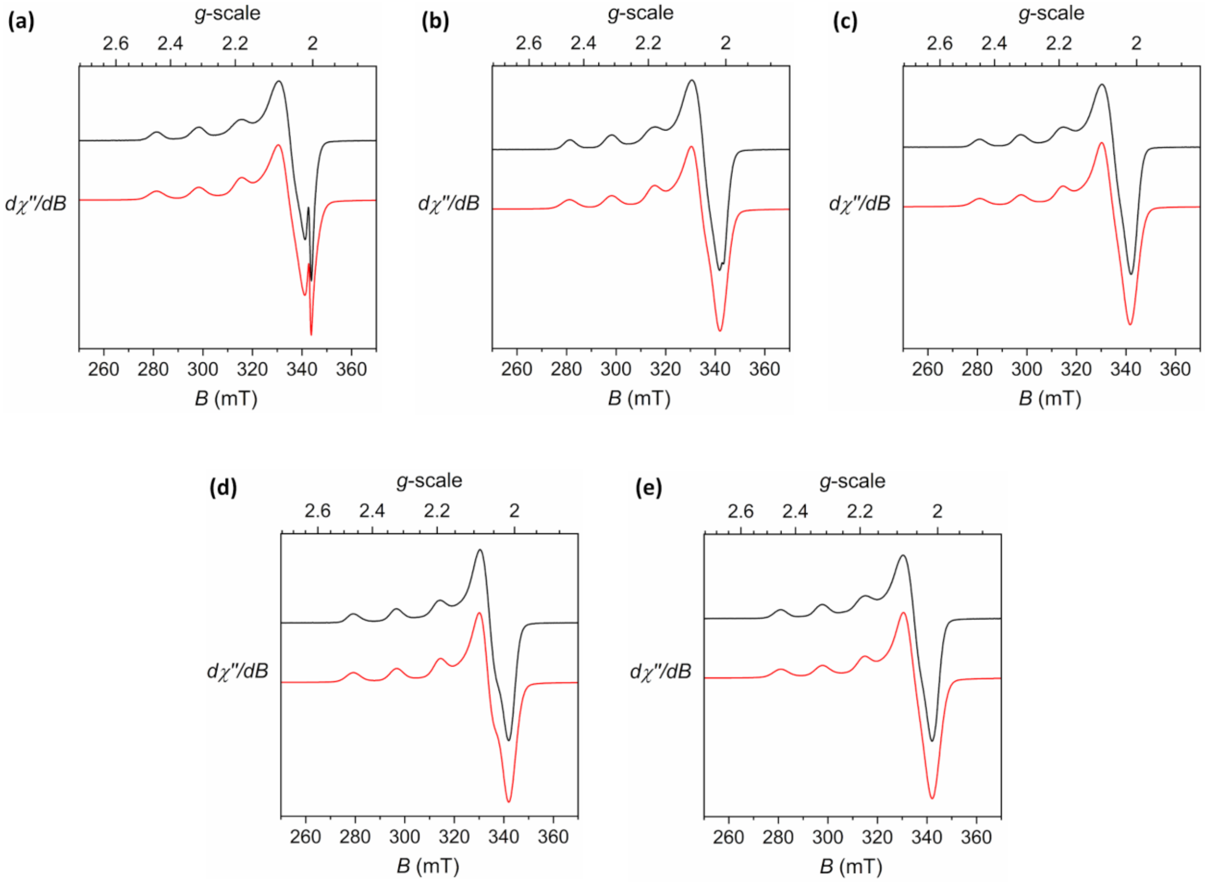

Figure S28. EPR spectra of 1 mM frozen solutions of [Cu^{II}(OAr-X)(**1**)]$^+$, produced by reaction of [Cu^{II}(**1**)(NCMe)](ClO_4)$_2$ with 2 equivalents of **(a)** MeO-ArOK, **(b)** MPPO-ArOK, **(c)** Me-ArOK, **(d)** Et-ArOK, and **(e)** ^{t}Bu-ArOK, both in THF solution at -90°C. Solutions of the potassium phenolate salts (X-ArOK) were obtained by combination of equimolar quantities of X-ArOH and ^{t}BuOK. Black and red lines correspond to experimental data and spectral simulations, respectively. In addition to copper(II) complex, there is also small amounts of the organic radical MeO-ArO$^\bullet$ in **(a)**.

Table S6. Spin Hamiltonian *g*- and *A*-values (10^{-4} cm^{-1}) derived from simulation of the EPR spectra of the products from reaction of [Cu^{II}(**1**)(NCMe)]$^{2+}$ with 2 equivalents of X-ArOK.

X	g_z	$g_{x,y}$	A_z	$A_{x,y}$
MeO	2.245	2.061	178	0
MPPO	2.246	2.062	178	2
Me	2.252	2.060	175	0
Et	2.255	2.064	184	5
^{t}Bu	2.250	2.058	176	0

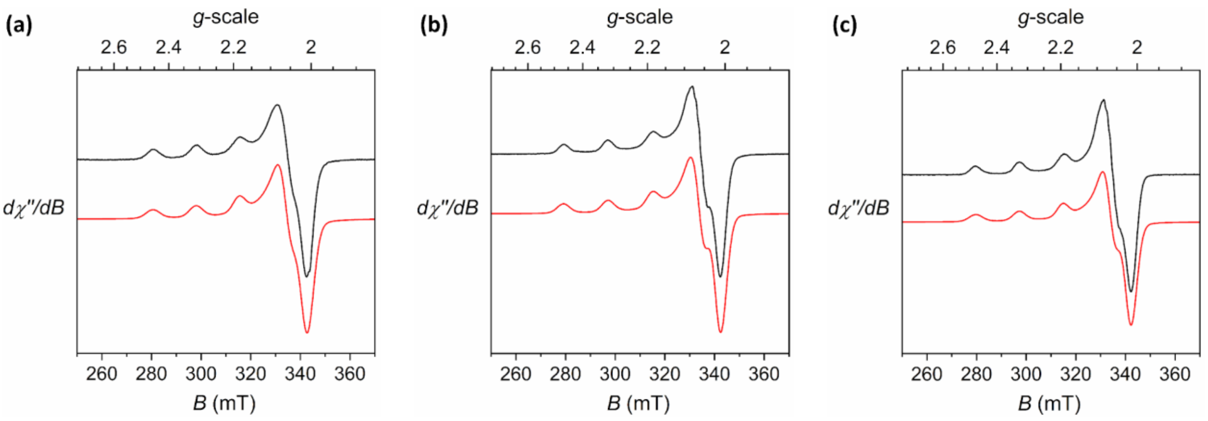

Figure S29. EPR spectra of the product mixtures obtained from reaction of $[Cu^{II}(\eta^1\text{-}O_2^{\bullet-})(\mathbf{1})]^+$ with **(a)** BNAH, **(b)** $^{Me}AcrH_2$, and **(c)** xanthene, in THF solution at -90°C. Black and red lines correspond to experimental data and spectral simulations, respectively.

Table S7. Spin Hamiltonian *g*- and *A*-values (10^{-4} cm^{-1}) derived from simulation of the EPR spectra of the products from reaction of $[Cu^{II}(\eta^1\text{-}O_2^{\bullet-})(\mathbf{1})]^+$ with C-H bond substrates.

Substrate	g_z	$g_{x,y}$	A_z	$A_{x,y}$
BNAH	2.246	2.060	182	10
$^{Me}AcrH_2$	2.250	2.064	188	4
xanthene	2.251	2.063	184	2

Quantification of Products from Reaction with Substrates, and Proposed Mechanisms for Their Formation

Quantification of Organic Products from Reaction with X-ArOH

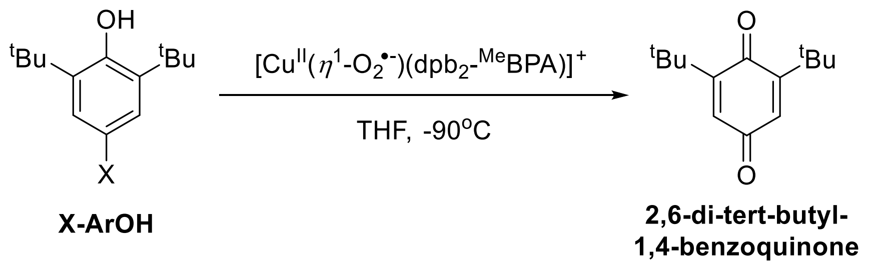

(Eqn. S1)

A cuvette was charged with 2 mL of a 0.5 mM solution of [Cu^{I}(**1**)][B(C_6F_5)$_4$] in THF, inside a nitrogen gas-filled glovebox, and capped with a septum. After being cooled to -90°C, dry dioxygen was bubbled through the solution for 200 s to give [Cu^{II}(η^1-$O_2^{\bullet-}$)(**1**)]$^+$. Next, 4-substituted (X) 2,6-di-*tert*-butylphenols (X-ArOH, where X = MeO, MPPO (2-methyl-1-phenylpropan-2-yloxy), Me, Et, ^{t}Bu) were added and reaction was run till completion (Eqn. S1). This was monitored using UV-vis spectroscopy. Work-up involved filtering the cold reaction mixture through a small pad of silica gel, to remove copper containing compounds, and washing the silica with an additional 2 mL of cold (-40°C) THF. Identification and quantification was performed by adding the internal standard naphthalene to the combined filtrates, and injecting the resulting mixture directly into a GC-MS instrument (starting oven temperature = 50°C (held for 1 min); temperature ramp = 40°C min^{-1} to 310°C (held for 2 min); inlet and detector temperatures = 50°C and 250°C; helium carrier gas column flow rate = 1.67 mL min^{-1}; split ratio = 1:7).

Table S8. Percentage yields of 2,6-di-tert-butyl-1,4-benzoquinone obtained from reaction of [Cu^{II}(η^1-$O_2^{\bullet-}$)(**1**)]$^+$ with X-ArOH.

Yield (%)	X-ArOH, X =				
	**MeO**	**MPPO**	**Me**	**Et**	**^{t}Bu**
**Run 1**	46	55	44	57	40
**Run 2**	50	49	42	42	40
**Run 3**	45	50	46	44	43
**Average**	47	51	44	48	41
**standard deviation**	3.0	3.5	2.2	8.4	1.8

Quantification of Organic Products from Reaction with C-H Bond Substrates

Quantification of the organic products from reaction of superoxocopper(II) complexes with C-H bond substrates was performed using ^{1}H NMR spectroscopy, by comparison of the peak integrations of the protons in said products with the aromatic protons of the internal standard 1,3,5-trimethoxybenzene, using the following formula:

$$\text{yield (\%)} = \frac{m_{internal\ standard} \times \left[\frac{i_{product}}{i_{internal\ standard}} \times \frac{n_{internal\ standard}}{n_{product}}\right]}{m_{Cu\ complex}} \times 100 \qquad \textbf{(Eqn. S2)}$$

where, *m* = number of moles, *i* = integrated area of the peaks, and *n* = number of protons the peaks correspond to.

1-benzyl-1,4-dihydronicotinamide (BNAH)

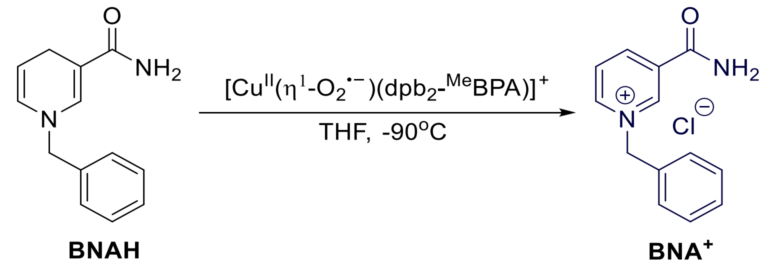

(Eqn. S3)

A cuvette containing 2 mL of a 1 mM solution of $[Cu^{I}(\mathbf{1})(NCMe)][B(C_6F_5)_4]$ in THF was capped with a septum, and cooled to -90°C. After allowing thermal equilibration at this temperature, dry dioxygen was bubbled through the solution for approximately 200 s. To the superoxocopper(II) complex formed, 10 equiv of the substrate BNAH (100 µL of a 0.2 M solution in THF) was added and reaction allowed to run until completion (i.e., 70 seconds). The resulting reaction mixture was reduced to dryness *in vacuo*, using a Schlenk line. This afforded a green residue, which was dissolved in DMSO-d_6 alongside 1.1 mg of 1,3,5-trimethoxybenzene and its ^{1}H NMR spectrum measured. Quantification was performed as described above (see Eqn. S2).

Table S9. Percentage yields of BNA^{+} obtained from reaction of $[Cu^{II}(\eta^{1}\text{-}O_2^{\bullet-})(\mathbf{1})]^{+}$ with BNAH.

Run 1	Run 2	Run 3	**Average**	**standard deviation**
36	46	42	**41**	**5.1**

***N*-methyl-9,10-dihydroacridine ($^{Me}AcrH_2$)**

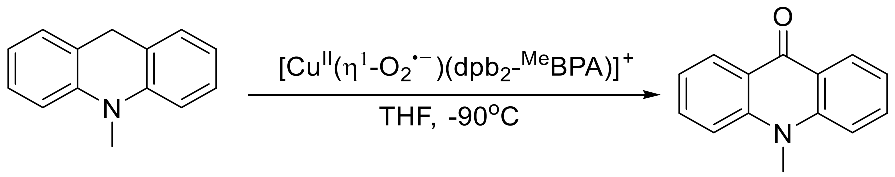

(Eqn. S4)

***N*-methyl-9,10-dihydroacridine** **_N_-methyl-9-acridone**

Quantification of the organic products obtained from reaction of $[Cu^{II}(\eta^1\text{-}O_2^{\bullet-})(\mathbf{1})]^+$ with *N*-methyl-9,10-dihydroacridine ($^{Me}AcrH_2$) was performed using the general procedure described above for BNAH. However, 50 equiv of $^{Me}AcrH_2$ (100 µL of a 1 M solution in tetrahydrofuran) was used in place of BNAH, completion of reaction required 8 h of stirring and, subsequently, the product mixture was quenched at -90°C by addition of 1.1 equiv of HCl (100 µL of a 22 mM solution in diethyl ether). Also, removal of solvents yielded mixtures of green and white solids, which were dissolved in $CDCl_3$ solution, alongside 1 µL of the internal standard 3,5-dimethylanisole. This allowed straightforward quantification of the *N*-methyl-9-acridone product. *N*-methylacridinium was also detected, but reliable/ highly reproducible yields provide elusive. We can say with certainty that, at least, ½ equiv *N*-methyl-acridinium ($MeAcrH^+$) was produced.

Table S10. Percentage yields of *N*-methyl-9-acridone obtained from reaction of $[Cu^{II}(\eta^1\text{-}O_2^{\bullet-})(\mathbf{1})]^+$ with $^{Me}AcrH_2$.

Run 1	Run 2	Run 3	**Average**	**standard deviation**
53	45	49	**49**	**3.6**

Xanthene

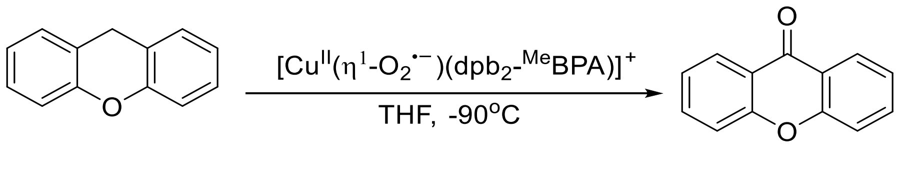

(Eqn. S5)

Xanthene **Xanthone**

A 25 ml Schlenk flask was charged with a magnetic stirring bar and 2 mL of a 1 mM solution of $[Cu^{I}(\mathbf{1})(NCMe)][B(C_6F_5)_4]$ in THF, and capped with a septum. It was cooled to -90°C using an ethanol/liquid nitrogen bath and maintained at that temperature throughout reaction. After

allowing thermal equilibration at this temperature, dry O_2 was bubbled through the solution for approximately 200 s. To the superoxocopper(II) complex thereby formed, 100 equiv. of xanthene (100 µL of a 2 M solution in tetrahydrofuran) was added and reaction was allowed to proceed to completion, which required 8 h. Solvent was removed from the reaction mixture by allowing it to warm to room temperature *in vacuo* (using a Schlenk line). The resulting residue was dissolved in 2 mL of dichloromethane and filtered through a small pad of silica gel, to remove copper containing compounds, after which the silica was washed with an additional 2 mL of dichloromethane. The filtrates were combined, and all volatiles removed *in vacuo*. The mixtures of green and white solids thereby obtained were dissolved in $CDCl_3$, alongside 1 µL of 3,5-dimethylanisole, and a ^1H NMR spectrum measured. Quantification was performed as described above (Eqn. S2).

Table S11. Percentage yields of xanthone obtained from reaction of $[Cu^{II}(\eta^1\text{-}O_2^{\bullet-})(\mathbf{1})]^+$ with xanthene.

Run 1	Run 2	Run 3	**Average**	**standard deviation**
50	52	44	**48**	**4**

Quantification of Hydrogen Peroxide

A cuvette charged with 2 mL of a 0.5 mM solution of $[Cu^I(\mathbf{1})][B(C_6F_5)_4]$ in THF was sealed with a rubber septum and cooled to -90°C. After allowing thermal equilibration at this temperature, dry dioxygen was bubbled through the solution for approximately 200 s. To the superoxocopper(II) complex formed, substrate was added and reaction allowed to proceed until completion. Subsequently, 50 µL of 20 mM HCl·Et_2O was added to the cuvette in the cryostat, also at -90°C. Upon allowing the product mixture to warm to room temperature, a 150 µL aliquot was removed and added to a cuvette pre-charged with 2 mL of a saturated solution of NaI in MeCN. This solution was incubated in the dark, at room temperature, for 5 min, and the UV-Vis spectrum of the I_3^-, thereby, formed was recorded. The amount of H_2O_2 present was determined using a standard calibration curve for the absorbance of I_3^- (at its λ_{max} of 362 nm) as a function of $[H_2O_2]$ (Figure S21). To build the calibration curve, stock solutions (0.1 mM – 0.58 mM) of H_2O_2 in acetonitrile were prepared from 3 % (w/w) H_2O_2 (ICM Pharma, medical grade), in a dark environment. 150 µL aliquots of these H_2O_2 solutions

were injected into 2 mL portions of saturated solutions of NaI in MeCN. After 5 min incubation at room temperature, UV-Vis spectra of the resulting solutions were recorded.

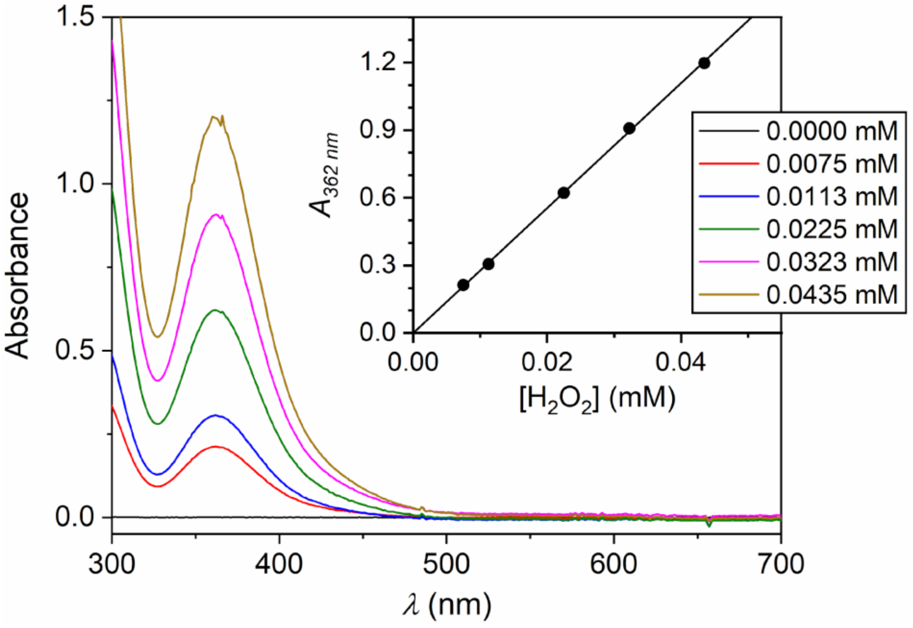

Figure S30. Main: UV-Vis spectra of I_3^- obtained from reaction of fixed concentrations of H_2O_2 with saturated solutions of NaI in acetonitrile, at room temperature. **Inset:** the absorbance of I_3^-, at ε_{max} = 362 nm, as a function of H_2O_2 concentration and its linear best fit line.

Table S12. Percentage yields of H_2O_2 obtained from reaction of $[Cu^{II}(\eta^1\text{-}O_2^{\bullet-})(\mathbf{1})]^+$ with various substrates. Estimated standard error in yields = ± 5 %.

Substrate	% Yield of H_2O_2
MeO-ArOH	91
MPPO-ArOH	110
Me-ArOH	89
Et-ArOH	87
^{t}Bu-ArOH	76
^{Me}AcrH$_2$	90
xanthene	59

Quantum Chemical Calculations

All electronic structure calculations presented in this paper were carried out using the ORCA 5.0.2 software package.[12] The Cu-O_2 complexes were interrogated for identification of O_2 binding modes (end-on vs. side-on) and ground electronic spin states (singlet vs. triplet), and vibrational analyses were performed in support of the experimental resonance Raman spectra. Unconstrained geometry optimizations and analytical frequency calculations of the model complexes were carried out at the DFT level, using the B3LYP hybrid GGA functional,[13-16] in conjunction with the RIJCOSX approximation.[17] The def2-TZVP basis set was used on all atoms below,[18] in conjunction with the corresponding def2/J auxiliary basis sets.[19] Initial atomic coordinates were taken from experimental single-crystal X-ray diffraction structures, where available. Dispersion effects were accounted for by Grimme's atom-pairwise D3 correction, utilising Becke-Johnson damping (D3BJ).[20,21] Stationary points were confirmed to be minima by the absence of imaginary frequencies. Attempts to locate side-on coordinated geometries in a triplet state were unsuccessful; they all converged to end-on structures. SCF energies of open-shell singlets (M_S = 0), which correspond to antiferromagnetically coupled states, were calculated using the broken-symmetry formalism.[22] Energies for the singlet state were calculated via the following equation:

$$E_S = E_T - 2\left(\frac{E_T - E_{BS}}{2 - \langle S^2 \rangle_{BS}}\right)$$

where E_S is the singlet SCF energy, E_{BS} is the calculated broken-symmetry singlet energy, E_T is the calculated triplet energy of the broken-symmetry singlet geometry, and $\langle S^2 \rangle$ is the expectation value of the total spin operator for the broken symmetry singlet. Absorption properties of all complexes were simulated by means of the TDDFT approach, using the CAM-B3LYP functional,[23] in combination with the aug-cc-pVDZ basis set on all atoms,[24-28] and the D3 dispersion correction. Effects from the solvent, THF, were accounted for by inclusion of the linear response CPCM scheme.[29] Calculation of EPR parameters (g-tensors and hyperfine coupling constants) for the phenoxocopper complexes were performed using the PBE0 hybrid functional,[30] in conjunction with the ZORA-recontracted[31] version of the def2-TZVPP basis set[18] on Cu and the ZORA-def2-TZVP for all other atoms. The fully decontracted def2/J auxiliary basis set was included for all atoms[19]. Scalar relativistic effects were treated using the zeroth-order regular approximation (ZORA).[32,33] The RIJCOSX approximation was used to

minimise computational time.[17] Tight convergence criteria and high-quality grids (DefGrid3) were used throughout. Spin-orbit coupling was treated using the spin-orbit mean-field (SOMF) operator,[34] with the 1X-approximation (SOCType 3).[35,36] One-electron terms were included for the construction of the potential, the Coulomb term was computed using the RI approximation, and exchange terms were incorporated via one-center exact integrals, including the spin-other orbit interaction, without local DFT correlation terms (SOCFlags 1,3,3,0). Picture change effects were also considered. XAS calculations were performed using previously established protocols using a time-dependent DFT approach.[37] TDDFT (K edge XAS) calculated spectra were shifted –5.5 eV to align with experimental energies. A 2 eV Gaussian broadening was applied to all calculated spectra. A sample input file for XAS calculations is provided in Figure S28 below.

```
!UKS RIJCOSX B3LYP ZORA ZORA-def2-TZVP AutoAux
! SlowConv TightSCF pal8 LargePrint UNO CPCM

%scf
MaxIter 700
end

%maxcore 11000

%tddft
orbwin[0]= 0,0,-1,-1
orbwin[1]= 0,0,-1,-1
doquad true
nroots 600
maxdim 50
end
```

Figure S31. Example input for XAS calculations in ORCA 5.0.2.

Single point energies for accurate spin-state energetics for the superoxo species were computed at the CASSCF and NEVPT2 levels of theory.[38-42] The active space species used contains 12 electrons in 12 orbitals (i.e., CAS(12,12)), comprised of five orbitals of 3d character located on Cu and one σ-bonding ligand orbital involving all ligand donor atoms. The Cu $3d_{z^2}$ orbital interacts with the in-plane oxygen π^*_σ orbital, whilst one further orbital in the active space represents the superoxo π^*_v orbital perpendicular to the CuO_2 plane. Taken together, up to this point, the $[CuO_2]^+$ moiety is described with a (12,7) active space. A doubleshell of

4d orbitals provide the remaining five orbitals of the full (12,12) active space. A similar choice of active space was employed in two previous studies of protein active sites that resemble the coordination geometry of the present complexes.[43,44] Scalar relativistic effects were included via the Douglas–Kroll–Hess (DKH) approach to the second order.[45-47] For the CASSCF/NEVPT2 calculations, the relativistically recontracted def2-QZVPP basis set was used for Cu and def2-TZVP was used for C, N, O and H.[18] The def2-TZVPP auxiliary basis set was utilised for the RI integral transformation step. In all calculations, MP2 natural orbitals, obtained from a RI-MP2 calculation using the same basis set specifications as detailed above for the CASSCF calculations, were used as reference orbitals. Results were visualised using the ChemCraft software package.[48]

Topographic steric maps of the DFT calculated triplet ground state geometries of the superoxocopper(II) complexes $[Cu^{II}(\eta^1\text{-}O_2^{\bullet-})(\mathbf{1})]^+$ and $[Cu^{II}(\eta^1\text{-}O_2^{\bullet-})(\mathbf{2})]^+$ were generated using the web-based tool SambVca (Version 2.1),[49,50] where the sphere radius *R* around the Cu centre was set to 5 or 10 Å and a mesh of 0.05 Å was used to scan the sphere. Atomic radii were scaled by 1.17.

Optimised Geometries

Of the geometry optimized structures located [Cu^{II}(η^1-O$_2^{\bullet-}$)(**1**)]$^+$, the triplet (S = 1) is the most stable stable. The next most stable state is the open-shell singlet, which is about 10 kcal mol^{-1} higher in energy. Efforts to locate a closed-shell Cu^{II}(η^1-O$_2^{\bullet-}$) structure failed. Similarly, attempts to calculate side-on bound [Cu(η^2-O$_2$)(**1**)]$^+$ were also largely unsuccessful. More specifically, a stable wavefunction could not be located for the closed-shell singlet, and geometry optimizations for the open-shell singlet and triplet states of [Cu(η^2-O$_2$)(**1**)]$^+$ converged to end-on bound Cu^{II}(η^1-O$_2^{\bullet-}$) structures (Figure S31 and Table S14).

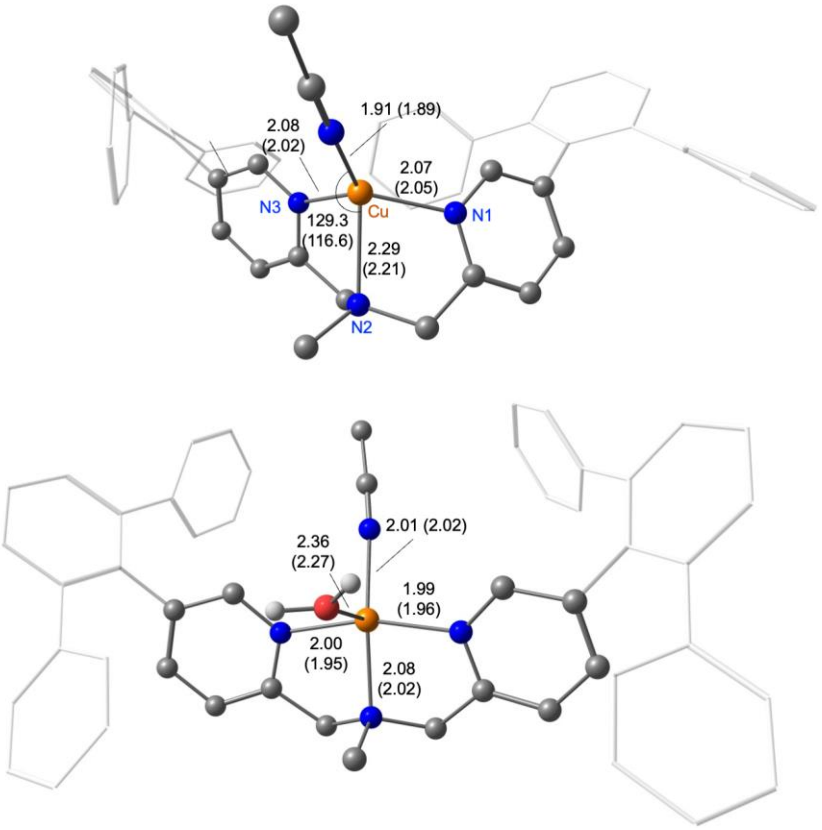

Figure S32. DFT calculated (B3LYP-D3/def2-TZVP) geometry optimized structures of [Cu^{I}(NCMe)(**1**)]$^+$ (S = 0) and [Cu^{II}(**1**)(NCMe)(OH$_2$)]$^{2+}$ (S = 1/2). Selected bond lengths (Å) and angles (°) are shown, with experimental values in parentheses. Hydrogen atoms have been omitted for clarity.

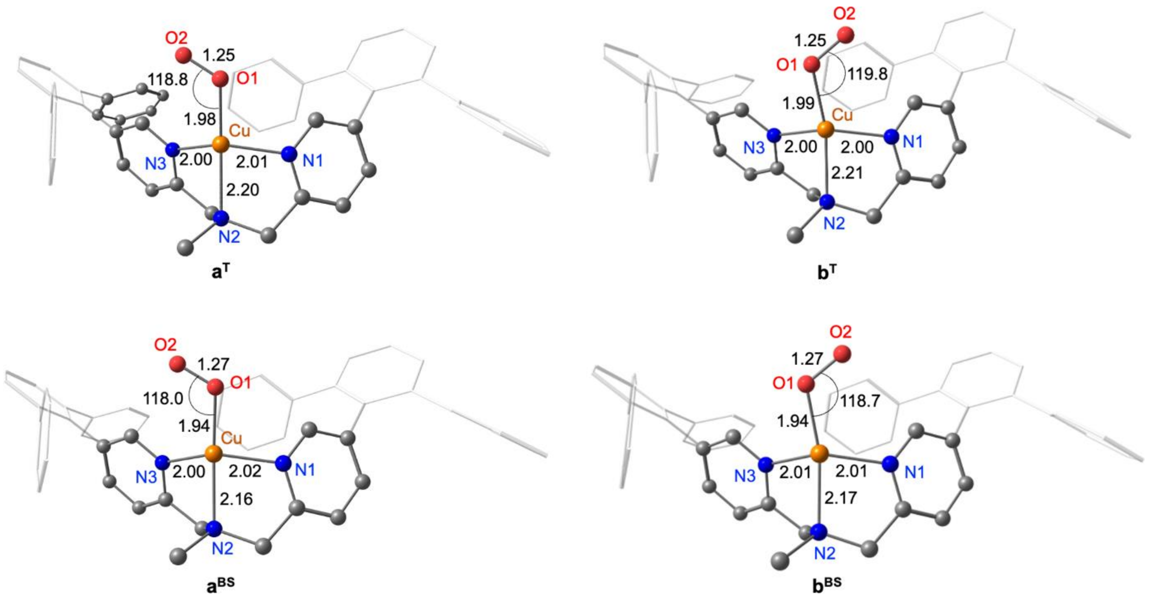

Figure S33. DFT calculated (B3LYP-D3/def2-TZVP) geometry optimized structures of [Cu^{II}(η^1-$O_2^{\bullet-}$)(**1**)]$^+$, in the electronic triplet (S = 1; **a^T** and **b^T**) and broken-symmetry singlet (M_S = 0; **a^{BS}** and **b^{BS}**) states. Selected bond lengths (Å) and angles (°) are provided. Hydrogen atoms have been omitted for clarity.

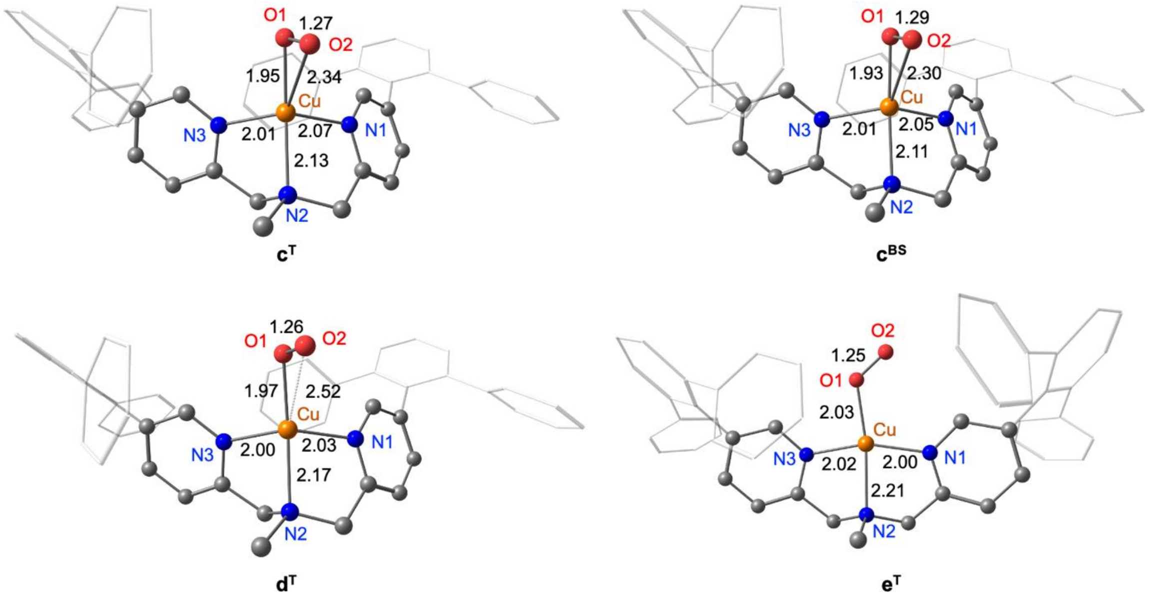

Figure S34. DFT calculated (B3LYP-D3/def2-TZVP) geometry optimized structures obtained from failed attempts to locate [Cu^{II}(η^2-$O_2^{\bullet-}$)(**1**)]$^+$ (i.e., a side-on superoxocopper(II) complex) in the electronic triplet (S = 1; **c^T**, **d^T** and **e^T**) and broken-symmetry singlet (M_S = 0; **c^{BS}**) states. Selected bond lengths (Å) and angles (°) are provided. Hydrogen atoms have been omitted for clarity.

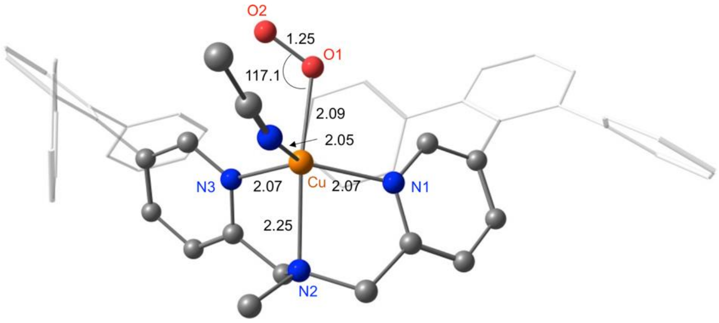

Figure S35. DFT calculated (B3LYP-D3/def2-TZVP) geometry optimized structures of the potential 5-coordinate complex $[Cu^{II}(\eta^1\text{-}O_2^{\bullet-})(\mathbf{1})(NCMe)]^+$ (S = 1). Selected bond lengths (Å) and angles (°) are shown. Hydrogen atoms have been omitted for clarity.

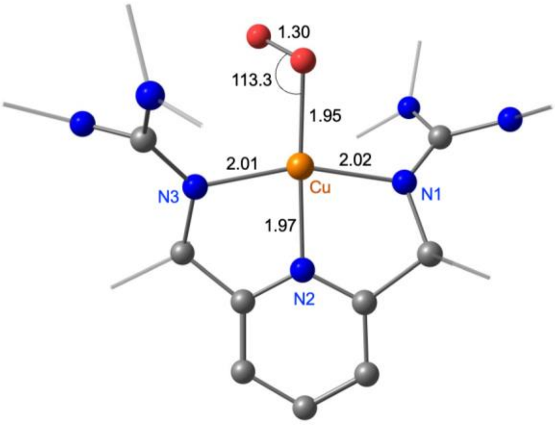

Figure S36. DFT calculated (B3LYP-D3/def2-TZVP) geometry optimized structures of $[Cu^{II}(\eta^1\text{-}O_2^{\bullet-})(TMGMP)]^+$, in the electronic triplet (S = 1) state. Selected bond lengths (Å) and angles (°) are provided. Hydrogen atoms have been omitted for clarity.

Table S13. Summary of key parameters, including bond lengths (Å), bond angles (°) and relative energies (kcal mol^{-1}), from DFT calculated (B3LYP-D3/def2-TZVP) geometry optimised structures of $[Cu^{II}(\eta^1\text{-}O_2^{\bullet-})(\mathbf{1})]^+$.

Parameters	Isomers			
	**a^T**	**b^T**	**a^{BS}**	**b^{BS}**
Cu–N1	2.014	2.004	2.015	2.006
Cu–N2	2.203	2.213	2.163	2.169
Cu–N3	2.000	2.004	2.002	2.006
Cu–O1	1.984	1.995	1.937	1.942
Cu···O2	2.811	2.833	2.772	2.785
O1–O2	1.253	1.251	1.271	1.271
Cu–O1–O2	118.8	119.8	118.0	118.7
N1–Cu–N2	81.7	81.9	81.9	82.2
N1–Cu–N3	137.8	139.4	138.4	139.7
N1–Cu–O1	98.1	109.1	96.7	109.1
N2–Cu–N3	81.3	80.9	81.4	81.1
N2–Cu–O1	163.5	163.5	165.9	164.7
N3–Cu–O1	108.6	96.9	108.0	95.5
τ_4 [51]	0.42	0.40	0.40	0.39
$\langle S^2 \rangle$	2.01	2.01	1.00	1.00
E_{rel}	0.00	0.32	10.27	10.62
$\boldsymbol{\rho}_{Cu}$	0.30	0.29	–0.37	–0.37
$\boldsymbol{\rho}_{O1}$	0.73	0.73	0.12	0.12
$\boldsymbol{\rho}_{O2}$	0.85	0.86	0.41	0.41

Table S14. Summary of key parameters, including bond lengths (Å), bond angles (°) and relative energies (kcal mol^{-1}), from DFT calculated (B3LYP-D3/def2-TZVP) geometry optimised structures obtained from failed attempts to locate $[Cu^{II}(\eta^2\text{-}O_2^{\bullet -})(\mathbf{1})]^+$ (i.e., a side-on superoxocopper(II) complex).

Parameters	Isomers			
	$\mathbf{c}^{T}$	$\mathbf{d}^{T}$	$\mathbf{c}^{BS}$	$\mathbf{e}^{T}$
Cu–N1	2.069	2.034	2.053	2.001
Cu–N2	2.133	2.166	2.108	2.211
Cu–N3	2.010	2.002	2.014	2.015
Cu–O1	1.955	1.965	1.929	2.032
Cu···O2	2.338	2.521	2.303	2.896
O1–O2	1.270	1.263	1.288	1.253
Cu–O1–O2	90.4	100.4	89.1	121.9
N1–Cu–N2	81.6	81.9	82.1	81.5
N1–Cu–N3	126.7	132.7	128.7	161.1
N1–Cu–O1	107.9	105.2	105.2	105.9
N2–Cu–N3	82.9	82.1	82.8	80.6
N2–Cu–O1	158.6	164.1	162.3	171.1
N3–Cu–O1	104.8	102.3	103.8	92.5
τ_4 [51]	0.53	0.45	0.49	0.20
$\langle S^2 \rangle$	2.01	2.01	1.01	2.01
E_{rel}	0.87	0.83	5.79	0.06
$\boldsymbol{\rho}_{Cu}$	0.34	0.32	0.42	0.29
$\boldsymbol{\rho}_{O1}$	0.74	0.73	–0.17	0.73
$\boldsymbol{\rho}_{O2}$	0.82	0.84	–0.39	0.84

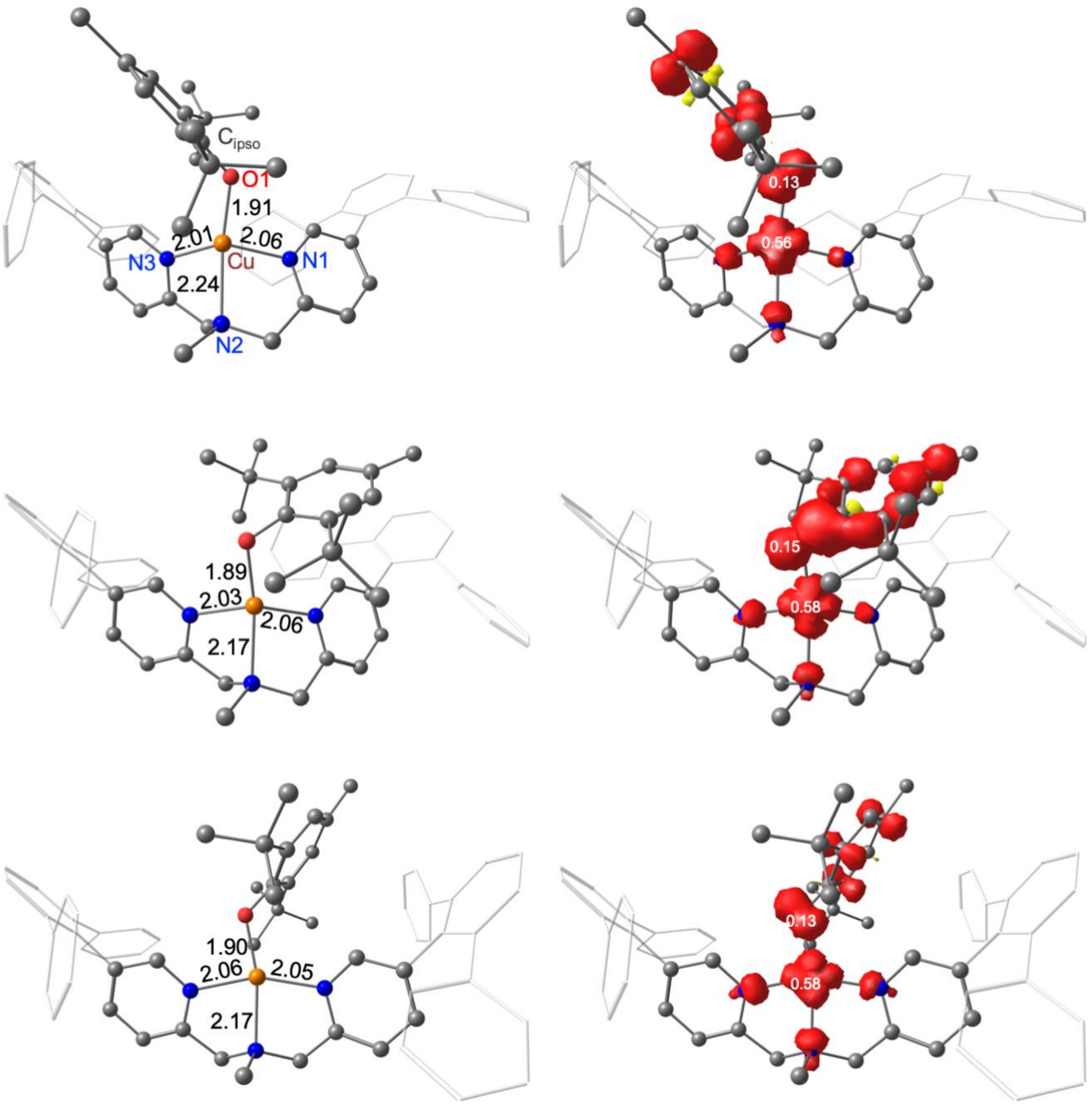

Figure S37. DFT calculated (B3LYP-D3/def2-TZVP) geometry optimized structures and spin density plots for conformers 1 (top), 2 (middle), and 3 (bottom) of [Cu^{II}(OAr-Me)(**1**)]$^+$ (S = ½). Selected bond lengths (Å) and angles (°), and spin populations are included. Hydrogen atoms have been omitted for clarity.

Table S15. Summary of key parameters, including bond lengths (Å), bond angles (°) and relative energies (kcal mol^{-1}), from DFT calculated (B3LYP-D3/def2-TZVP) geometry optimised structures of [Cu^{II}(OAr-Me)(**1**)]$^+$ (S = 1/2).

Parameters	Conformer		
	**1**	**2**	**3**
Cu–N1	2.058	2.055	2.046
Cu–N2	2.240	2.171	2.171
Cu–N3	2.010	2.028	2.058
Cu–O1	1.912	1.891	1.898
Cu–O1–C_{ipso}	128.6	116.7	127.7
N1–Cu–N2	78.5	81.5	81.3
N1–Cu–N3	131.8	131.5	159.2
N1–Cu–O1	97.0	112.4	110.6
N2–Cu–N3	79.5	80.1	77.9
N2–Cu–O1	166.5	161.3	168.1
N3–Cu–O1	112.2	97.8	90.1
τ_4 [51]	0.44	0.48	0.23
$\langle S^2 \rangle$	0.76	0.76	0.76
E_{rel}	4.4	0.0	3.7
ρ_{Cu}	0.37	0.40	0.42
ρ_{O1}	0.22	0.23	0.21
$\rho_{N1,2,3}$	0.11	0.13	0.17

Topographic Steric Maps

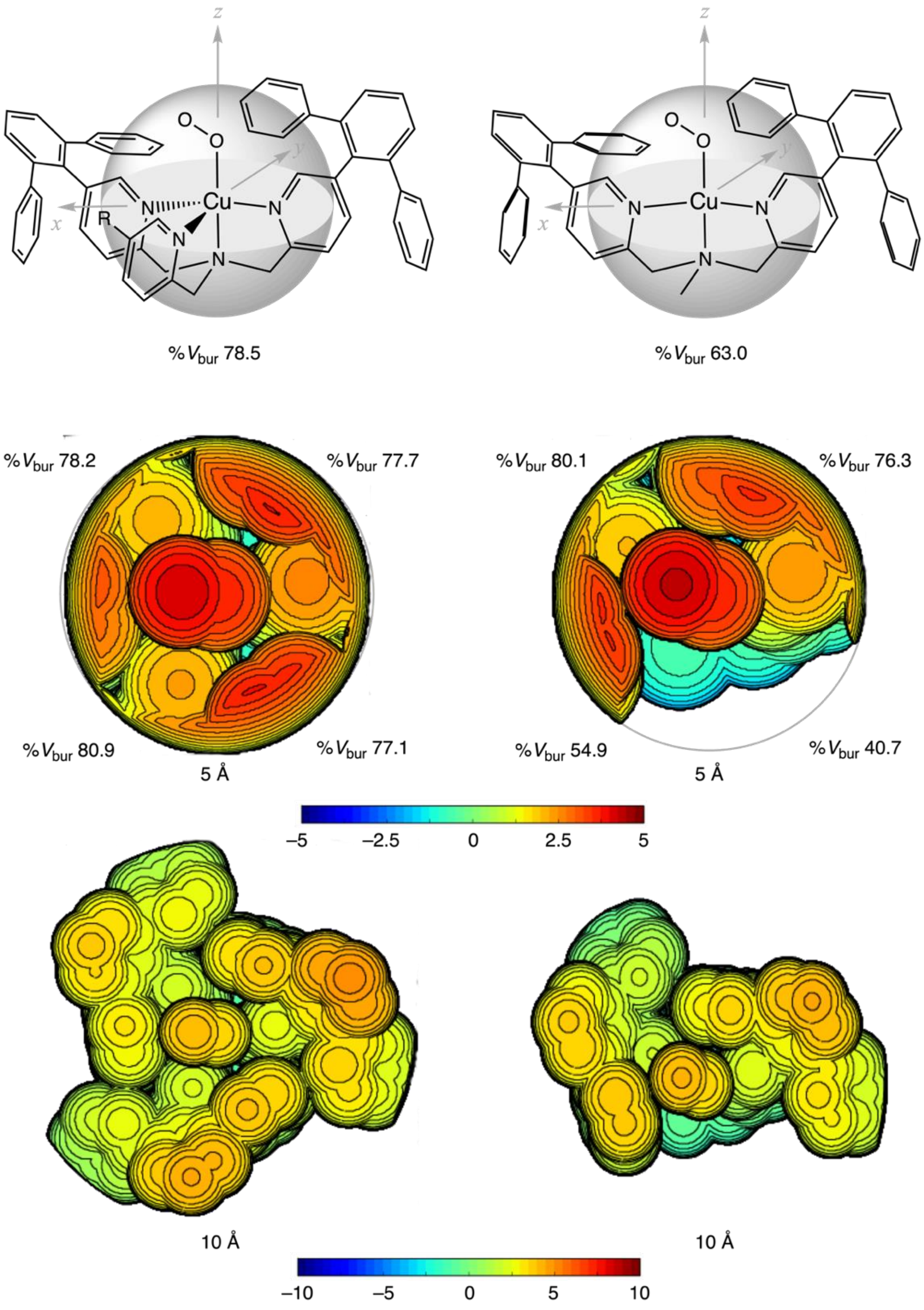

Figure S38. Topographic steric maps of the DFT calculated geometry optimized structures of $[Cu^{II}(\eta^1\text{-}O_2^{\bullet-})(\mathbf{2})]^+$ (left) and isomer $\mathbf{a}^T$ of $[Cu^{II}(\eta^1\text{-}O_2^{\bullet-})(\mathbf{1})]^+$ (right), with their Cu-O bonds (i.e., z-axis) oriented perpendicular to the plane of the page and pointing towards the viewer. The distance above or below the Cu ions (Å), which are set as the origin, is color-coded according to the figure key.

Molecular Orbital Diagram for $[Cu^{II}(\eta^1\text{-}O_2^{\bullet-})(1)]^+$

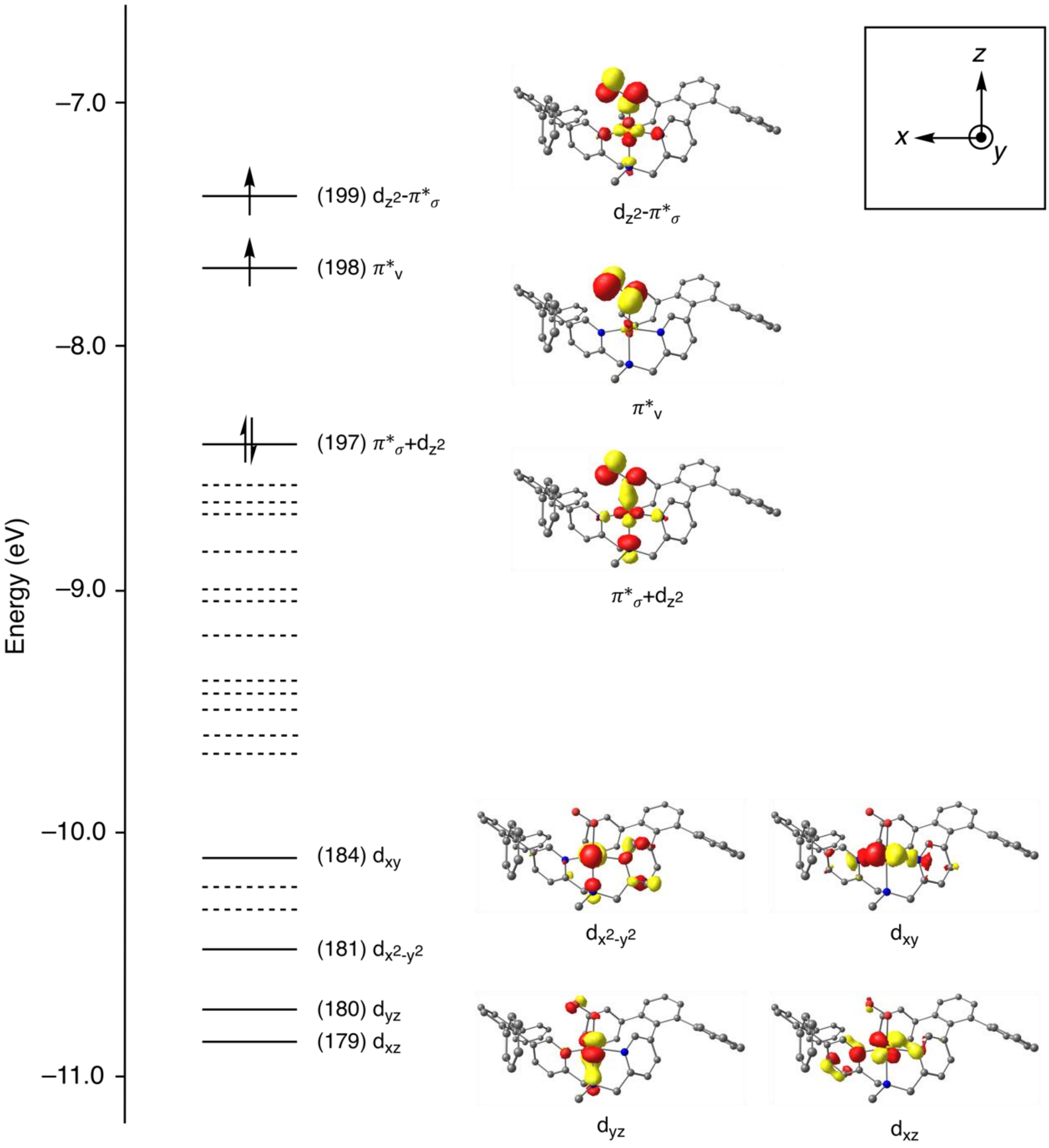

Figure S39. DFT calculated (B3LYP-D3/def2-TZVP) MO diagram depicting quasi-restricted orbitals of isomer **a**$^{\mathbf{T}}$ of $[Cu^{II}(\eta^1\text{-}O_2^{\bullet-})(\mathbf{1})]^+$ (isosurface = 0.05 au), in the triplet (S = 1) state.

CASSCF/NEVPT2 Calculations

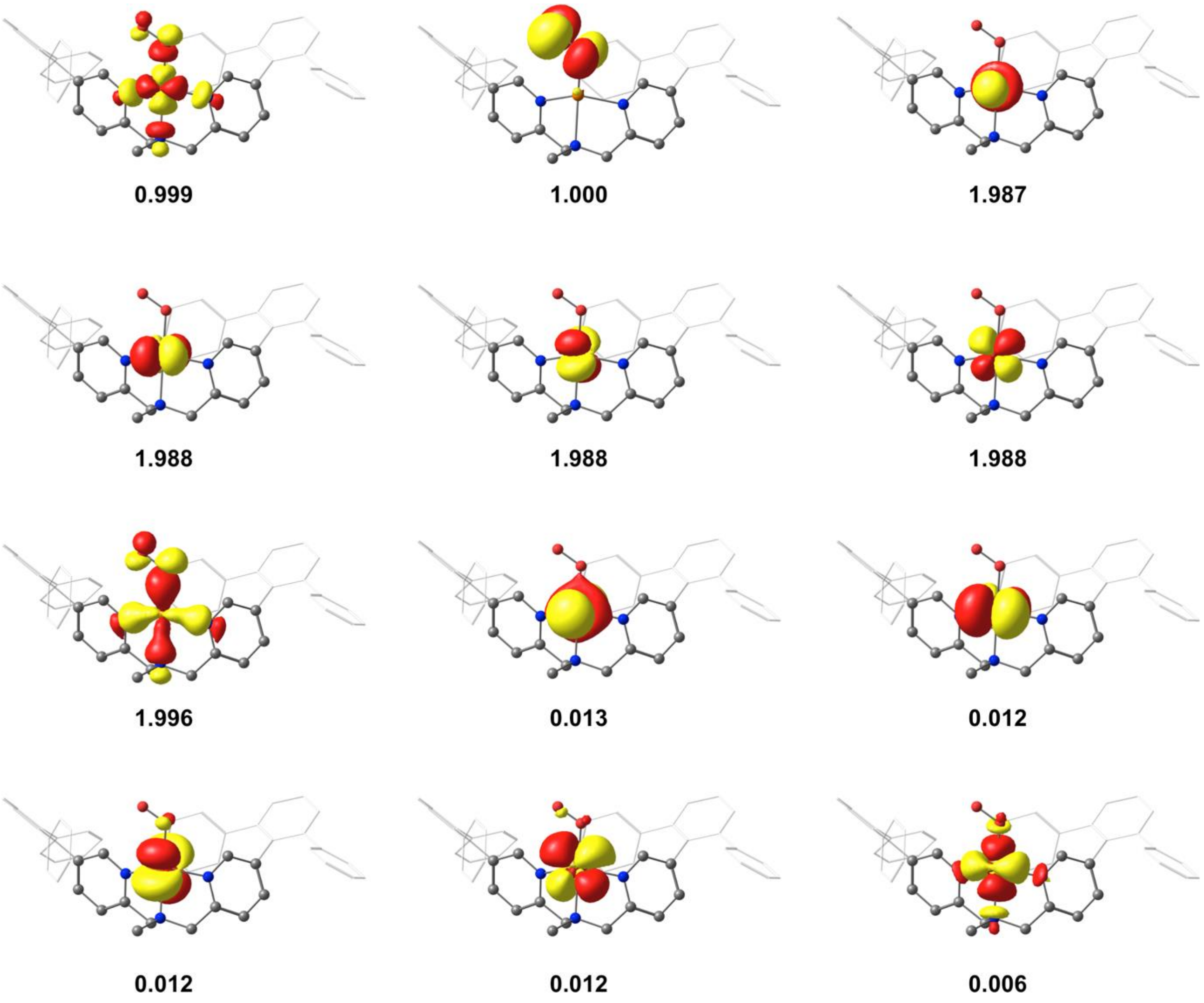

Figure S40. Natural active orbitals (isosurface = 0.05 au) and their occupation numbers from CAS(12,12)/NEVPT2 calculation of the triplet state of $[Cu^{II}(\eta^1\text{-}O_2^{\bullet-})(\mathbf{1})]^+$.

Table S16. Dominant configurations and associated weights contributing to the triplet state in the CAS(12,12) space used.

CAS occupation pattern	weights
[222221100000]	0.97113
[222111111000]	0.00285
[221211110100]	0.00268

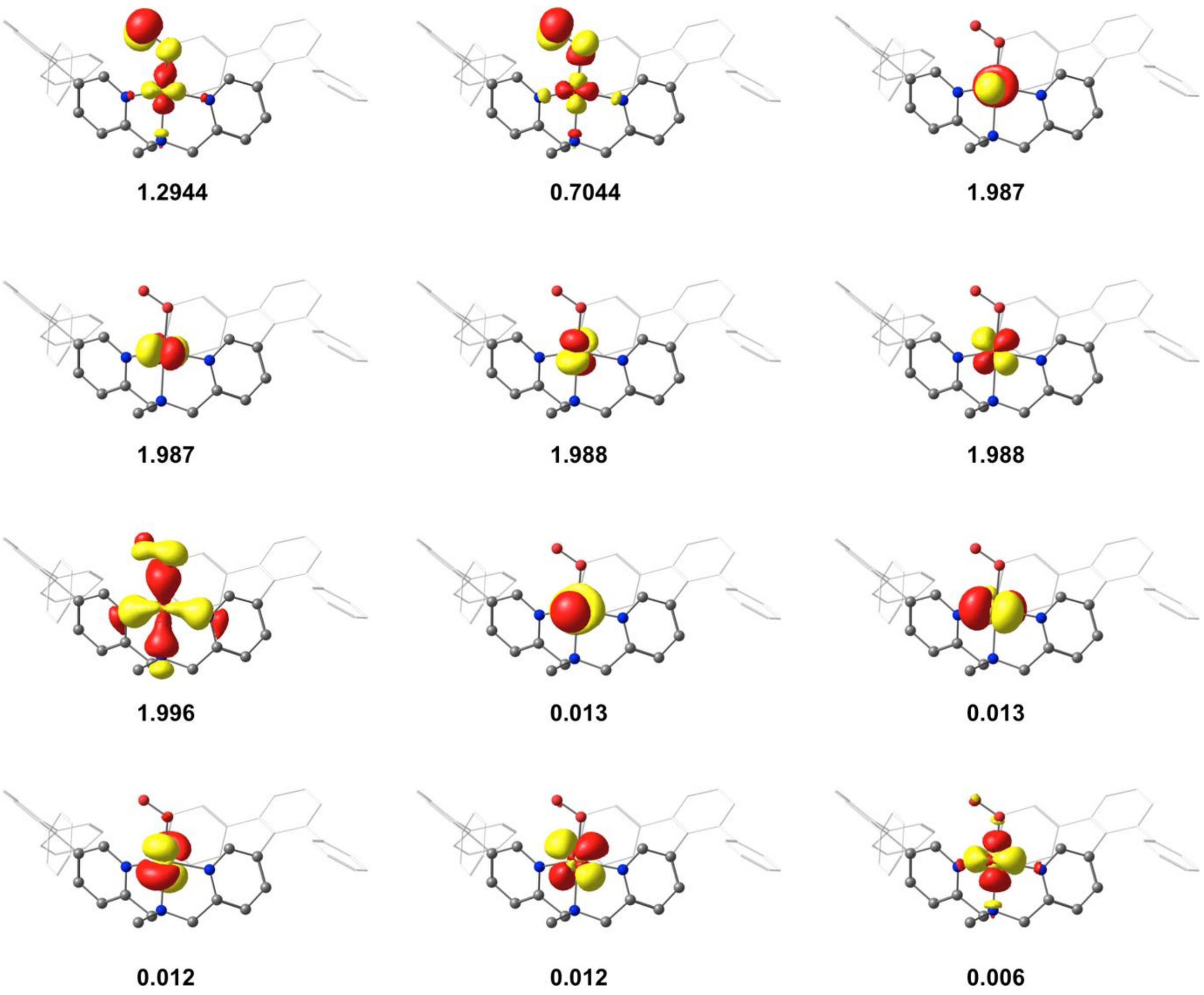

Figure S41. Natural active orbitals (isosurface = 0.05 au) and their occupation numbers from CAS(12,12)/NEVPT2 calculation of the singlet state of $[Cu^{II}(\eta^1\text{-}O_2^{\bullet-})(\mathbf{1})]^+$.

Table S17. Dominant configurations and associated weights contributing to the singlet state in the CAS(12,12) space used.

CAS occupation pattern	weights
[222222000000]	0.62982
[222220200000]	0.34110

Table S18. Singlet-triplet state energy splittings ($\Delta E_{ST} = E_S - E_T$) predicted for $[Cu^{II}(\eta^1\text{-}O_2^{\bullet-})(\mathbf{1})]^+$ using DFT, CASSCF, and NEVTPT2 levels of theory.

	ΔE_{ST} / kcal mol^{-1}
B3LYP	+9.61
CAS(12,12)	–2.38
CAS(12,12)/NEVPT2	–1.16

Table S19. CASSCF and NEVPT2 calculated total energies (a.u.) for $[Cu^{II}(\eta^1\text{-}O_2^{\bullet-})(\mathbf{1})]^+$.

	CASSCF(12,12)	NEVPT2
Singlet	–3846.112456	–3855.426616
Triplet	–3846.108670	–3855.428458

Vibrational Spectroscopy

Table S20. DFT calculated vibrational frequencies (cm^{-1}) for the ν(Cu-^{16}O) and ν(^{16}O-^{16}O) normal modes of $[Cu^{II}(\eta^1\text{-}O_2^{\bullet-})(\mathbf{1})]^+$. The corresponding predicted $^{18}O_2$ isotope shifts (cm^{-1}) are given in parentheses.

	$^{16}O_2$ (Δ^{18}O)	Cu^{16}O (Δ^{18}O)
$\mathbf{a}^T$ $S = 1$ Cu(η^1-O_2)	1299 (–70)	356 (–16)
$\mathbf{b}^T$ $S = 1$ Cu(η^1-O_2)	1305 (–73)	354 (–23)
$\mathbf{c}^T$ $S = 1$ Cu(η^1-O_2)	1267 (–72)	374 (–13)
$\mathbf{d}^T$ $S = 1$ Cu(η^1-O_2)	1272 (–72)	369 (–13)
$\mathbf{e}^T$ $S = 1$ Cu(η^1-O_2)	1299 (–76)	346 (–10)
$\mathbf{a}^{BS}$ $M_S = 0$ Cu(η^1-O_2)	1236 (–72)	426 (–37)
$\mathbf{b}^{BS}$ $M_S = 0$ Cu(η^1-O_2)	1237 (–72)	425 (–43)
$\mathbf{c}^{BS}$ $M_S = 0$ Cu(η^1-O_2)	1209 (–68)	427 (–18)<br>151 (–12)
**Expt.**	1137 (–63)	464 (–17)

TDDFT Calculated UV-Vis Spectra

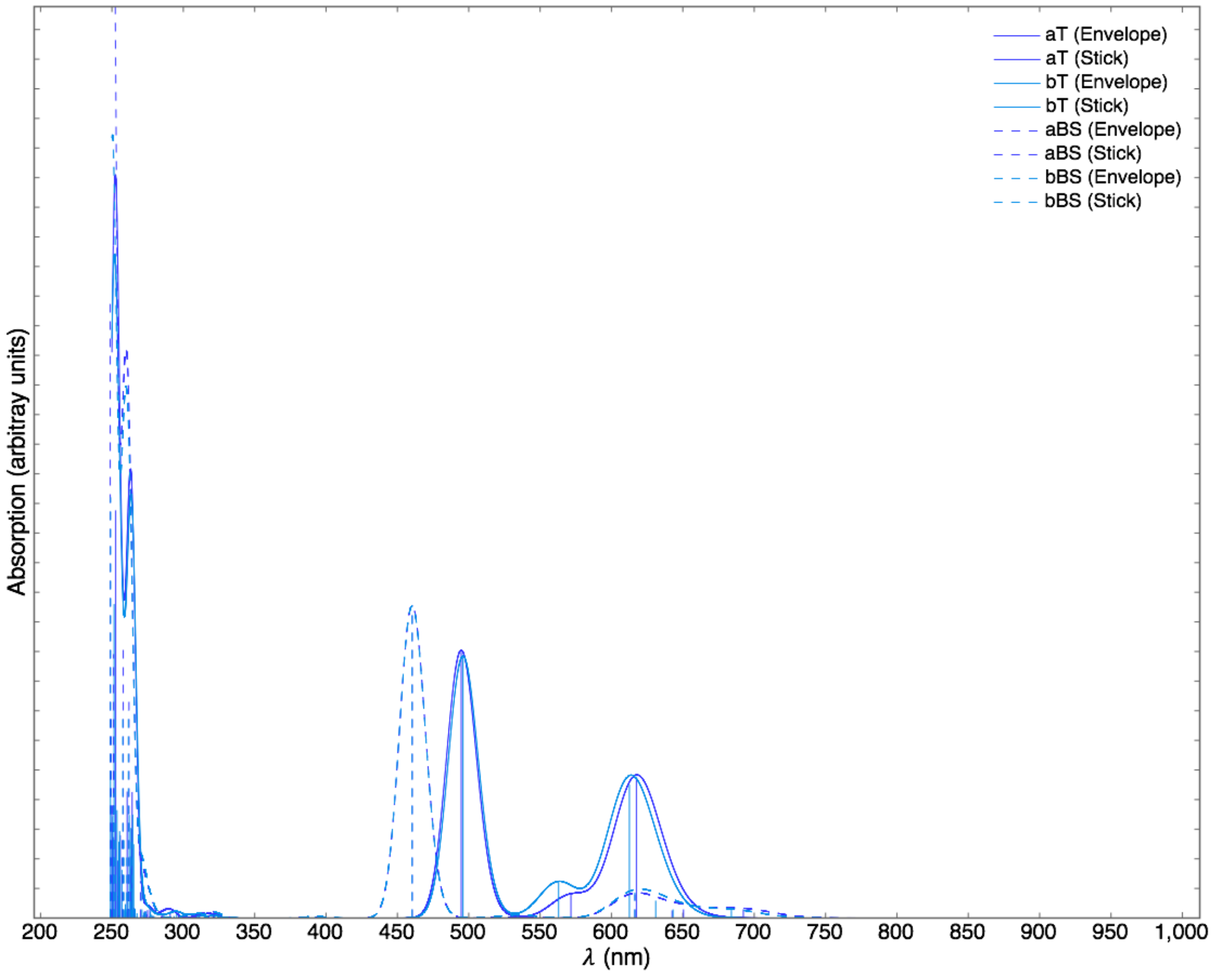

Figure S42. TDDFT calculated (CAM-B3LYP/aug-cc-pVDZ, THF) spectra of isomers $\mathbf{a}^{\mathbf{T}}$ and $\mathbf{b}^{\mathbf{T}}$ (solid lines, S = 1) and $\mathbf{a}^{\mathbf{BS}}$ and $\mathbf{b}^{\mathbf{BS}}$ (dashed lines, M_S = 0) for complex $[Cu^{II}(\eta^1\text{-}O_2^{\bullet-})(\mathbf{1})]^+$.

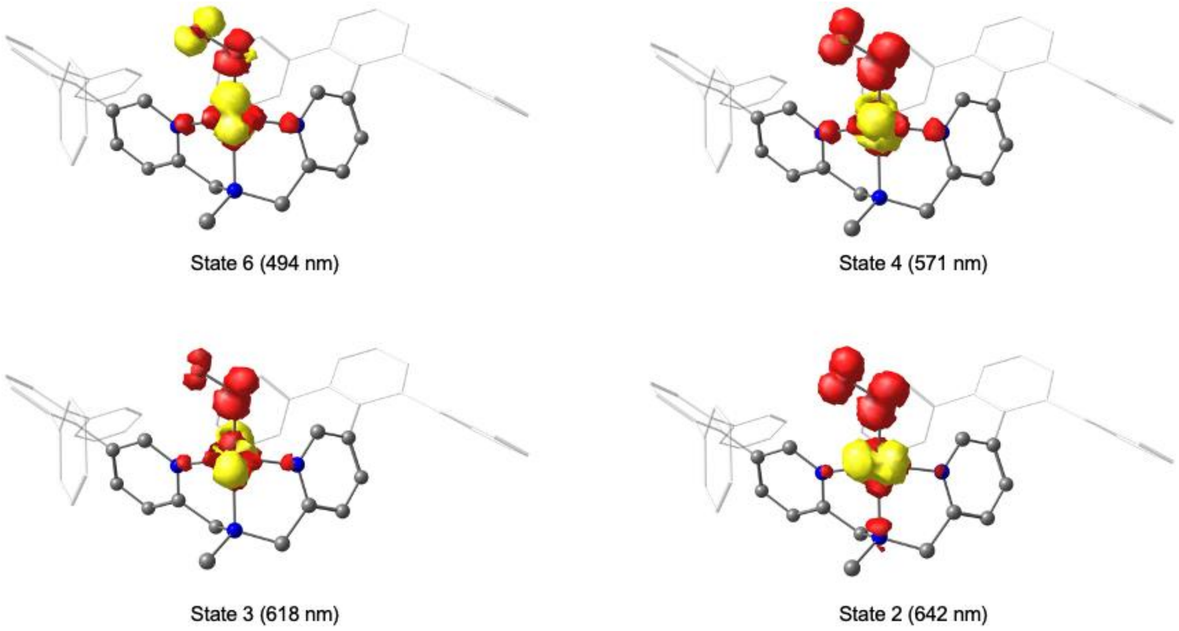

Figure S43. Difference densities (isosurface 0.005 au, CAM-B3LYP/aug-cc-pVDZ, THF) of selected transitions for isomer $\mathbf{a}^{\mathbf{T}}$ (S = 1) of $[Cu^{II}(\eta^1\text{-}O_2^{\bullet-})(\mathbf{1})]^+$. Color code: red = charge accumulation, yellow = charge depletion.

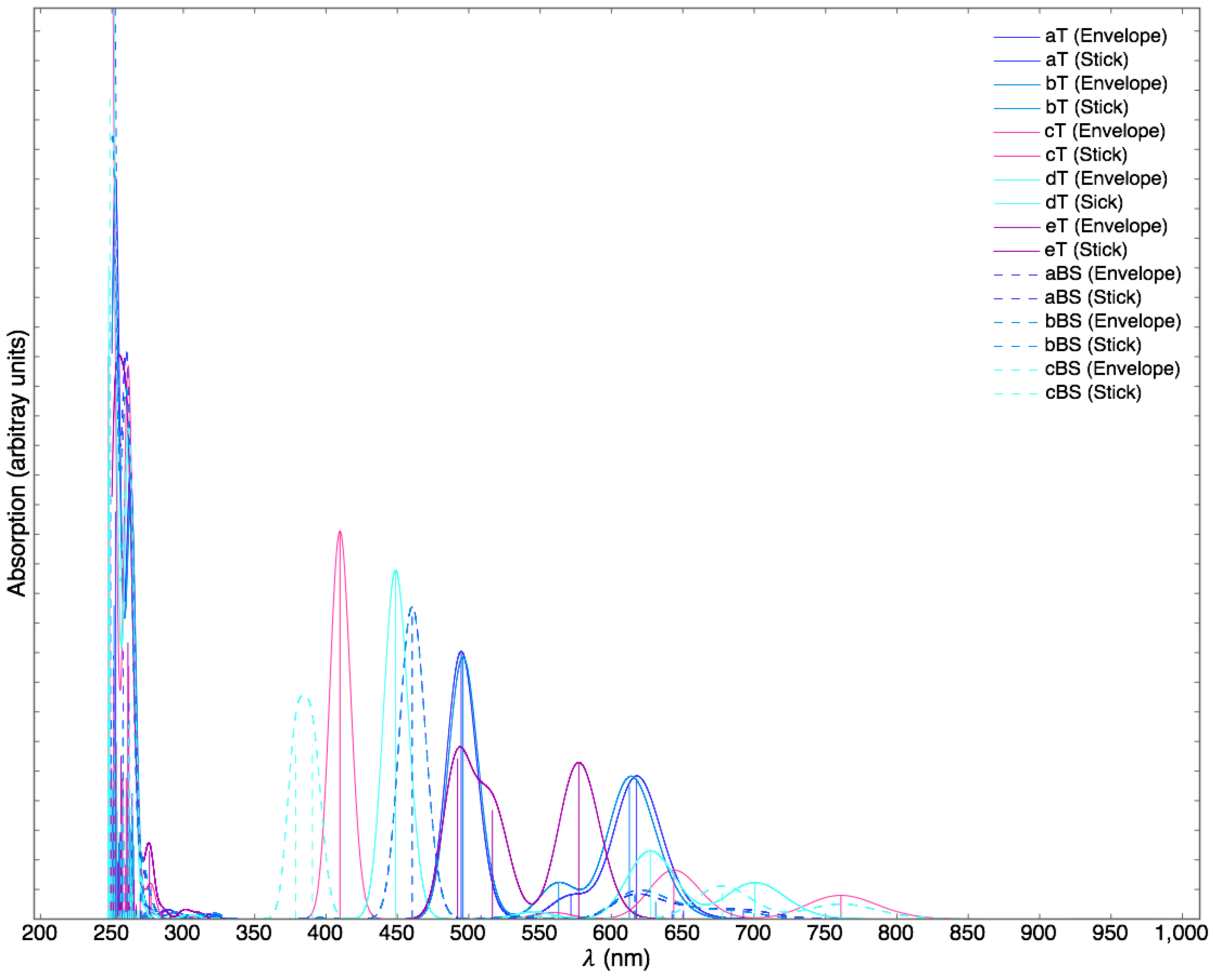

Figure S44. TDDFT calculated (CAM-B3LYP/aug-cc-pVDZ, THF) spectra of isomers $\mathbf{a}^{\mathbf{T}}$, $\mathbf{b}^{\mathbf{T}}$, $\mathbf{c}^{\mathbf{T}}$, $\mathbf{d}^{\mathbf{T}}$ and $\mathbf{e}^{\mathbf{T}}$ (solid lines, S = 1) and $\mathbf{a}^{\mathbf{BS}}$ and $\mathbf{b}^{\mathbf{BS}}$ (dashed lines, M_S = 0) for $[Cu^{II}(\eta^1\text{-}O_2^{\bullet-})(\mathbf{1})]^+$.

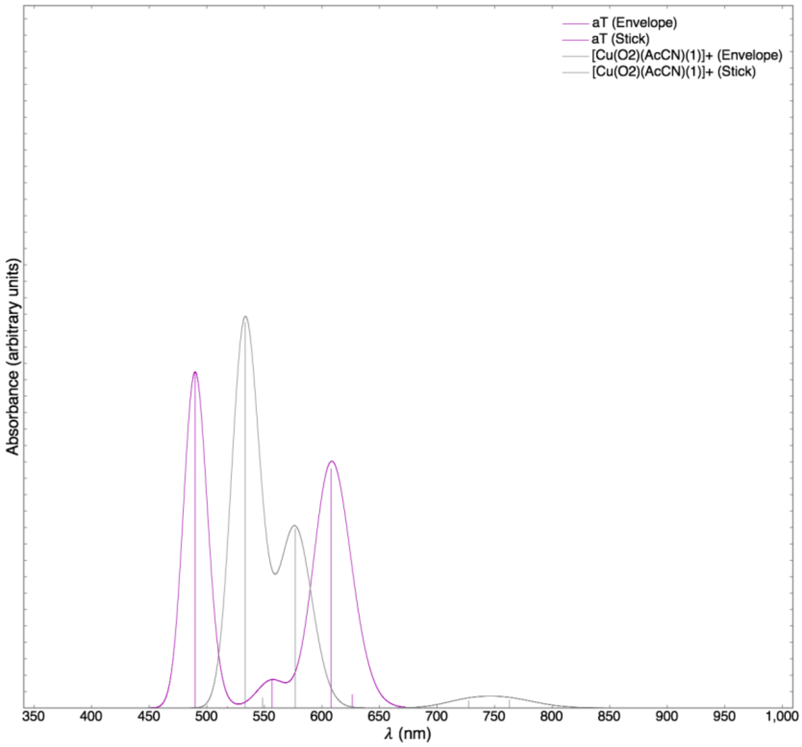

Figure S45. TDDFT calculated (CAM-B3LYP/aug-cc-pVDZ, THF) spectra of isomer $\mathbf{a}^{\mathbf{T}}$ (S = 1) and 5-coordinate solvento complex $[Cu^{II}(\eta^1\text{-}O_2^{\bullet-})(\mathbf{1})(NCMe)]^+$ (S = 1).

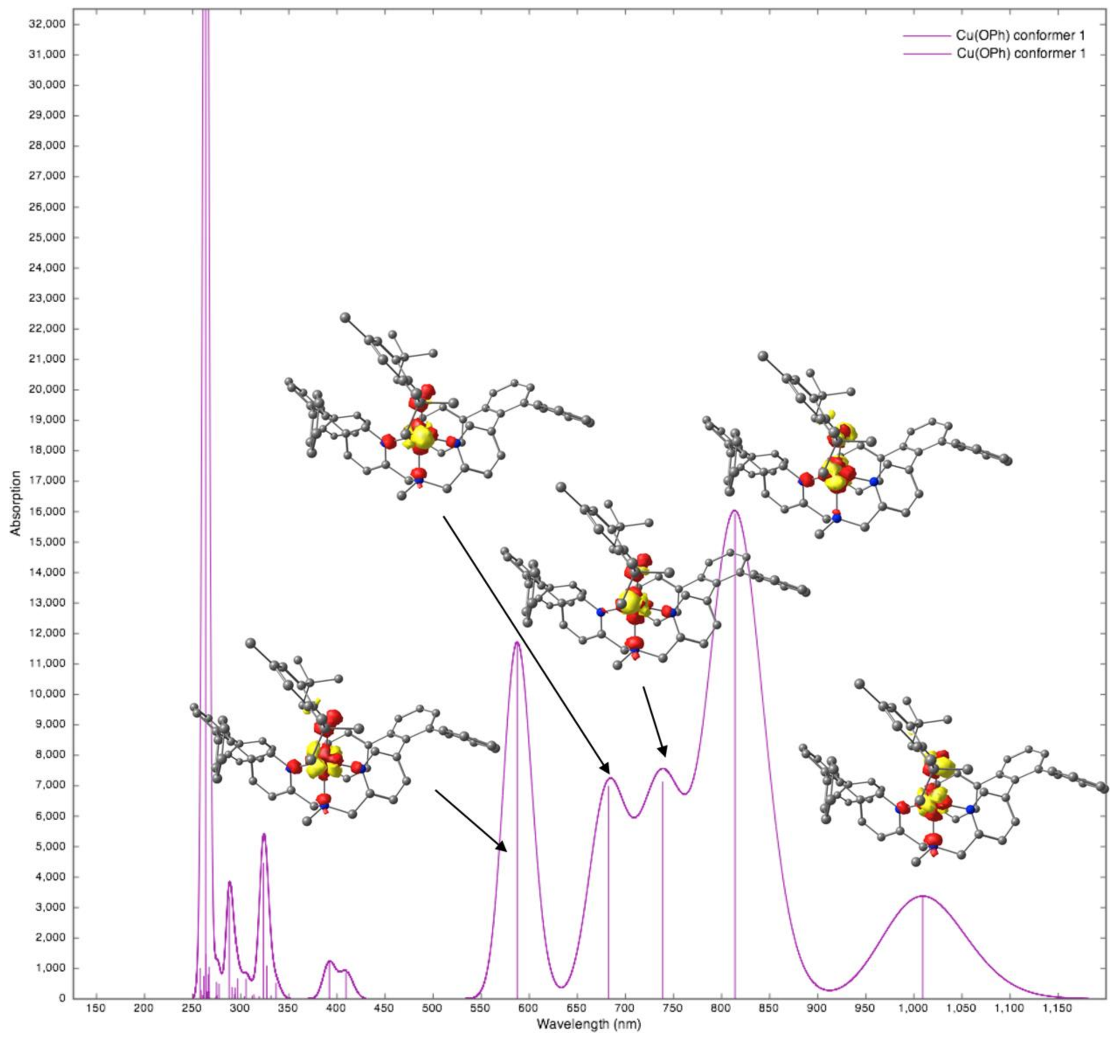

Figure S46. TDDFT calculated (CAM-B3LYP/aug-cc-pVDZ, THF) spectrum of conformer 1 of $[Cu^{II}(OAr\text{-}Me)(\mathbf{1})]^{+}$ (*S* = 1/2). Transition density difference maps are included (yellow: charge depletion; red: charge accumulation).

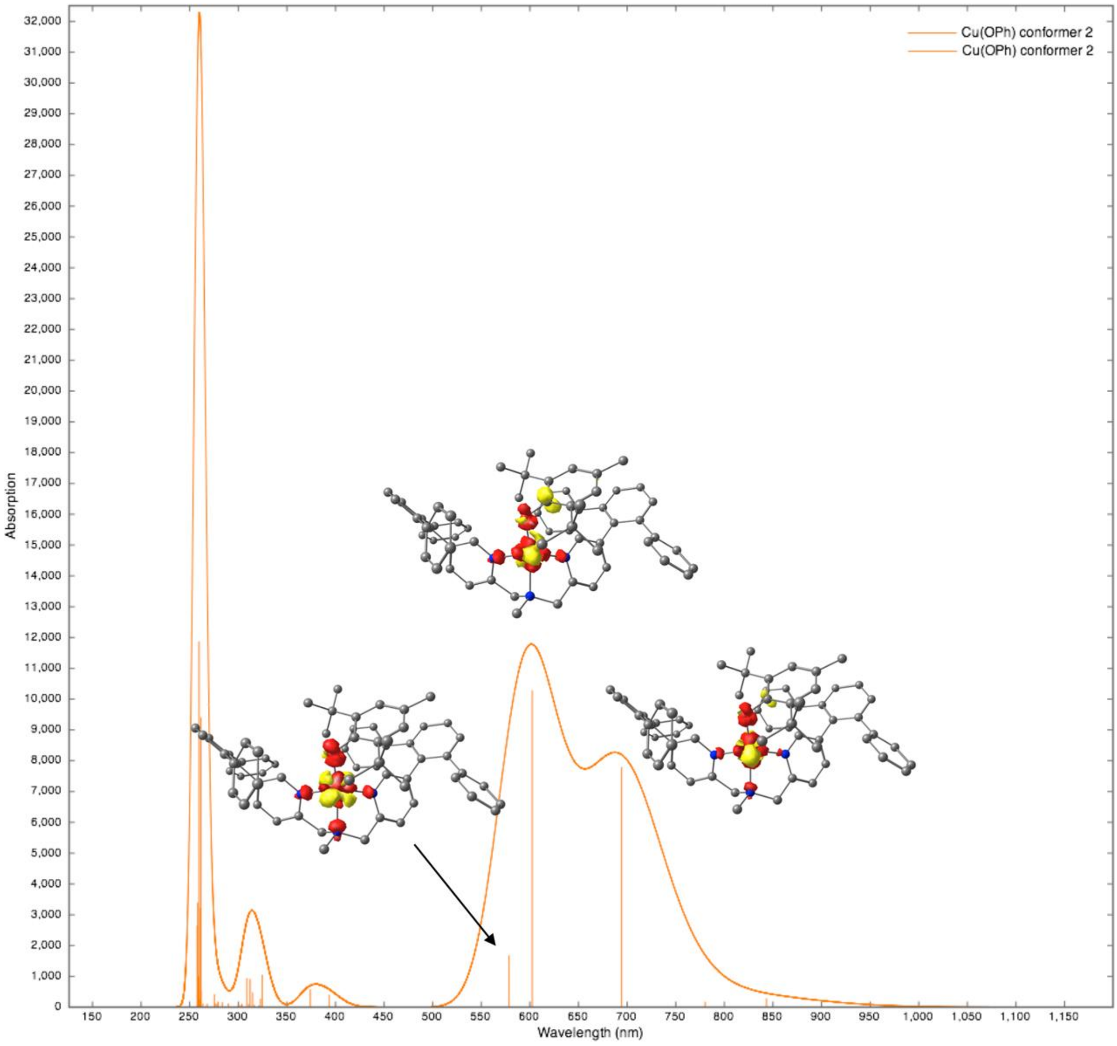

Figure S47. TDDFT calculated (CAM-B3LYP/aug-cc-pVDZ, THF) spectrum of conformer 2 of $[Cu^{II}(OAr\text{-}Me)(\mathbf{1})]^+$ (S = 1/2). Transition density difference maps are included (yellow: charge depletion; red: charge accumulation).

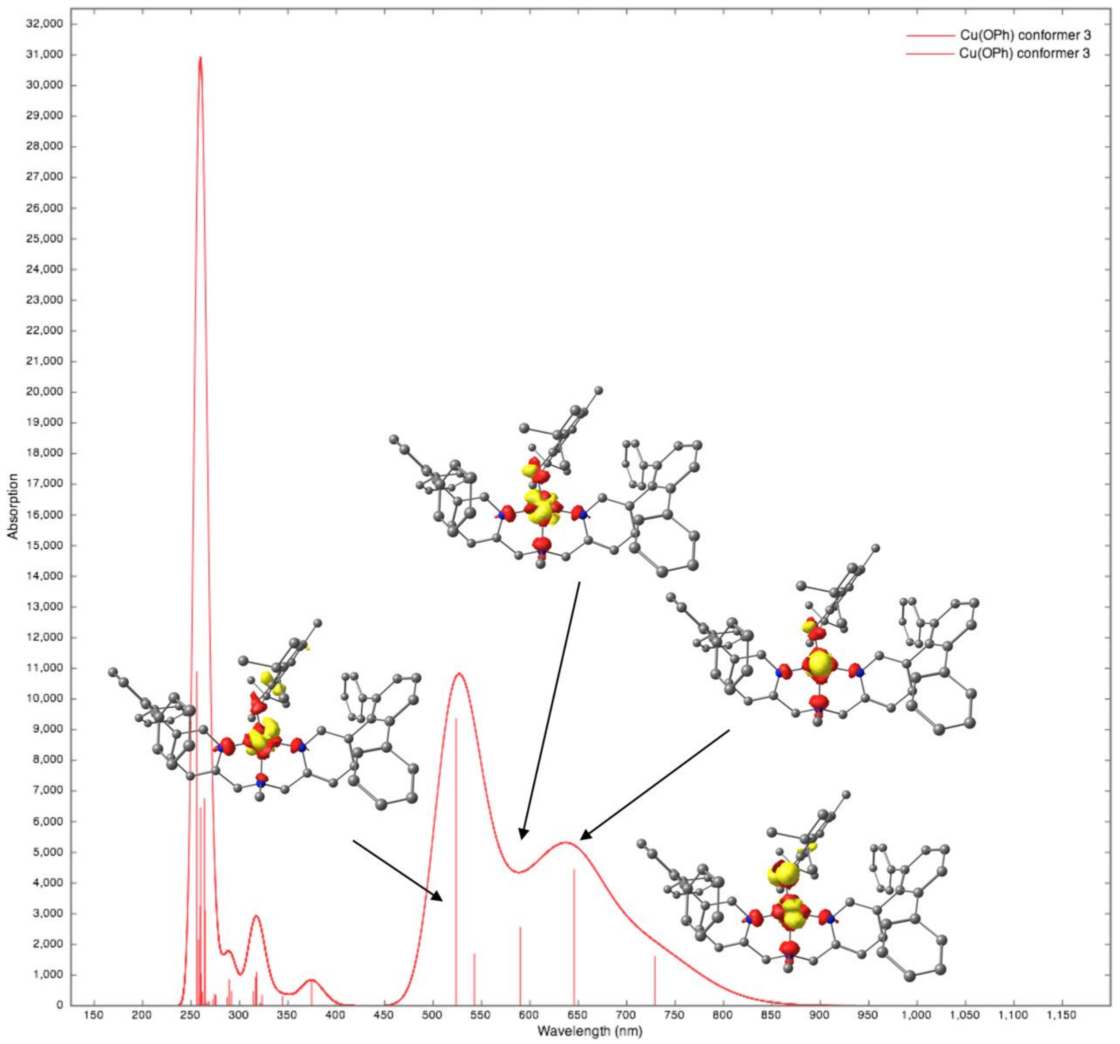

Figure S48. TDDFT calculated (CAM-B3LYP/aug-cc-pVDZ, THF) spectrum of conformer 3 of [Cu^{II}(OAr-Me)(**1**)]$^+$ (S = 1/2). Transition density difference maps are included (yellow: charge depletion; red: charge accumulation).

DFT Calculated EPR Parameters

Table S21. DFT calculated g-values and hyperfine coupling constants for various conformers of $[Cu^{II}(OAr\text{-}Me)(dpb_2\text{-}^{Me}BPA)]^+$ (S = 1/2).

Parameters	Conformer		
	**1**	**2**	**3**
g_x	2.034	2.026	2.041
g_y	2.082	2.087	2.060
g_z	2.201	2.186	2.175
a_x (py)	15, 20	14, 22	24, 27
a_y (py)	15, 21	15, 23	25, 28
a_z (py)	21, 28	21, 30	32, 37
a_{iso} (py)	17, 23	17, 25	27, 31
a_x (amine)	17	16	18
a_y (amine)	17	16	18
a_z (amine)	29	27	32
a_{iso} (amine)	20	19	23

DFT Calculated XAS

To better understand the origin of the differences between the experimental XAS spectra of $[Cu^{I}(\mathbf{1})(NCMe)]^+$, $[Cu^{II}(\mathbf{1})(NCMe)(OH_2)]^{2+}$, and the triplet and singlet states of $[Cu^{II}(\eta^1\text{-}O_2^{\bullet-})(\mathbf{1})]^+$ (isomers $\mathbf{a^T}$ and $\mathbf{a^{BS}}$, respectively), TDDFT calculations were performed using geometry optimized structures (vide infra). For $[Cu^{I}(\mathbf{1})(NCMe)]^+$, the high intensity of the calculated feature at 8981 eV matches well with experiment, and is found to arise from a transition to an orbital with high 4p character. This is consistent with expectations. The pre-edge feature in the calculated spectrum of $[Cu^{II}(\mathbf{1})(NCMe)(OH_2)]^{2+}$, while significantly less intense than expected, appears at a position (8977.1 eV) matching experiment. The feature in the rising edge of $[Cu^{II}(\mathbf{1})(NCMe)(OH_2)]^{2+}$, at 8985 eV, is seen in both the calculated and experimental spectrum. Spectra were calculated for both the triplet and singlet states of $[Cu^{II}(\eta^1\text{-}O_2^{\bullet-})(\mathbf{1})]^+$ (Figure S44). The closest agreement to experimental data was observed for triplet state

(isomer $\mathbf{a^T}$). The calculated spectrum of the triplet state captures the slight energetic shift of the pre-edge compared to [Cu^{II}(**1**)(NCMe)(OH$_2$)]$^{2+}$ (0.4 eV in both experiment and calculation), as well as the relative shift in the energy of the rising edge versus [Cu^{I}(**1**)(NCMe)]$^{+}$ and [Cu^{II}(**1**)(NCMe)(OH$_2$)]$^{2+}$. Conversely, the calculated pre-edge of the singlet state of [Cu^{II}(η^1-O$_2^{\bullet-}$)(**1**)]$^{+}$ (isomer $\mathbf{a^{BS}}$), at 8979.0 eV, appears at much higher energy than experiment (8977.7 eV). This poor energetic match disfavors assignment as the singlet state.

To probe the possibility that oxygenation of [Cu^{I}(**1**)(NCMe)]$^{+}$ yields a 5-coordinate acetonitrile-bound superoxocopper(II) complex, [Cu^{II}(η^1-O$_2^{\bullet-}$)(**1**)(NCMe)]$^{+}$, TDDFT calculation of the XAS spectrum of this species was performed using the geometry optimised structure of the triplet (S = 1) ground state, detailed above. The resulting XAS spectrum (Figure S49a) shows a rising edge energetically close to that calculated for [Cu^{I}(**1**)(NCMe)]$^{+}$ and much lower than the experimental value. Thus, the match to experimental data is very poor and, accordingly, the formulation [Cu^{II}(η^1-O$_2^{\bullet-}$)(**1**)(NCMe)]$^{+}$ can be discounted.

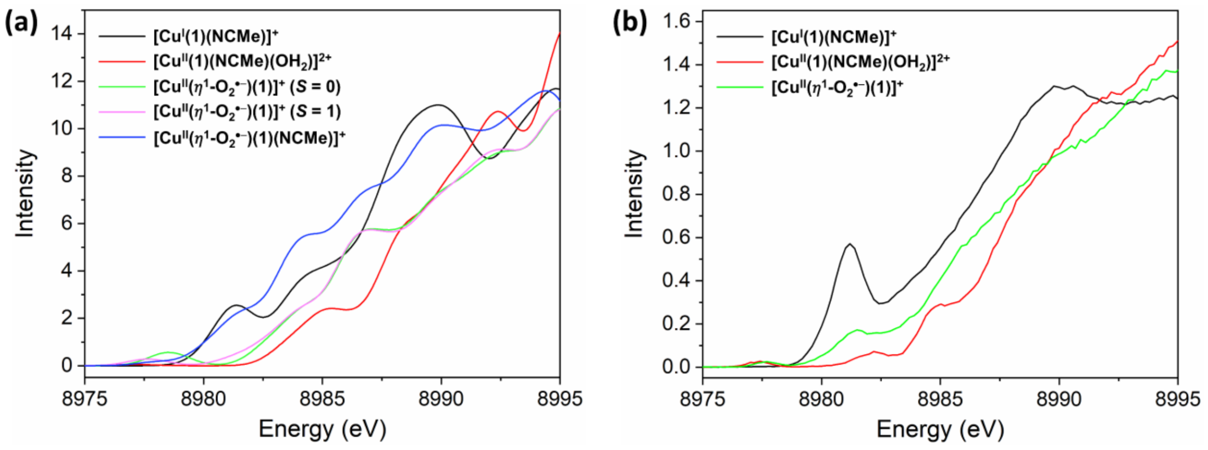

Figure S49. Left: calculated XAS spectra of [Cu^{I}(**1**)(NCMe)]$^{+}$, [Cu^{II}(**1**)(NCMe)(OH$_2$)]$^{2+}$, the singlet and triplet states of [Cu^{II}(η^1-O$_2^{\bullet-}$)(**1**)]$^{+}$, and [Cu^{II}(η^1-O$_2^{\bullet-}$)(**1**)(NCMe)]$^{+}$ depicted using black, red, green, pink, and blue lines, respectively. **Right:** experimental XAS spectra of [Cu^{I}(**1**)(NCMe)]$^{+}$, [Cu^{II}(**1**)(NCMe)(OH$_2$)]$^{2+}$, and [Cu^{II}(η^1-O$_2^{\bullet-}$)(**1**)]$^{+}$ (black, red, and green lines, respectively).

Atomic Coordinates

98

E(B3LYP-D3BJ) = -3827.771668452494 Eh

$[Cu^{I}(1)(NCMe)]^{+}$ (S = 0)

C	2.192373000	-1.277867000	1.593399000
H	2.619056000	-1.832250000	2.440430000
H	2.973419000	-0.605947000	1.227236000
C	1.814806000	-2.243411000	0.495348000
C	2.367636000	-3.512351000	0.406204000
H	3.106081000	-3.829697000	1.130749000
C	1.937236000	-4.377872000	-0.588683000
H	2.335166000	-5.381634000	-0.652041000
C	0.949308000	-3.963200000	-1.476413000
C	0.502459000	-2.650088000	-1.355882000
H	-0.240689000	-2.266798000	-2.041193000
C	0.289166000	-4.877193000	-2.440872000
C	-1.102219000	-5.072861000	-2.322851000
C	-1.754969000	-5.911047000	-3.223693000
H	-2.822151000	-6.062027000	-3.125982000
C	-1.041180000	-6.574869000	-4.211542000
H	-1.554835000	-7.229833000	-4.903097000
C	0.331246000	-6.404318000	-4.308475000
H	0.887609000	-6.913451000	-5.084363000
C	1.013723000	-5.552845000	-3.437159000
C	2.479116000	-5.378381000	-3.594021000
C	3.048338000	-4.113255000	-3.758496000
H	2.409273000	-3.240408000	-3.781385000
C	4.420242000	-3.967716000	-3.910238000
H	4.844292000	-2.981382000	-4.049252000
C	5.248600000	-5.084814000	-3.894630000
H	6.318423000	-4.970841000	-4.010887000
C	4.693335000	-6.349272000	-3.738322000
H	5.329807000	-7.224654000	-3.728409000
C	3.319124000	-6.494447000	-3.593492000
H	2.890796000	-7.480524000	-3.464164000
C	-1.864783000	-4.423407000	-1.222350000
C	-2.872088000	-3.498386000	-1.497626000
H	-3.128347000	-3.284655000	-2.527937000
C	-3.515528000	-2.824130000	-0.465852000
H	-4.281138000	-2.095879000	-0.701260000
C	-3.173426000	-3.076049000	0.856528000
H	-3.663487000	-2.543814000	1.658538000
C	-2.200844000	-4.027364000	1.142753000
H	-1.950876000	-4.253894000	2.171866000
C	-1.552914000	-4.693986000	0.112854000
H	-0.790736000	-5.427466000	0.340286000
C	-0.004443000	-1.222620000	2.651904000
H	0.223884000	-1.395620000	3.711212000
H	-0.067497000	-2.198965000	2.166612000
C	-1.343693000	-0.546074000	2.509615000
C	-2.251165000	-0.446426000	3.552507000
H	-2.007271000	-0.858469000	4.522747000
C	-3.453492000	0.214777000	3.348049000
H	-4.156288000	0.329548000	4.161440000
C	-3.735920000	0.767722000	2.100509000
C	-2.788598000	0.568097000	1.097260000
H	-2.962335000	0.951818000	0.101134000
C	-4.935935000	1.607305000	1.858494000
C	-6.224404000	1.130997000	2.168303000
C	-7.330615000	1.955433000	1.962196000
H	-8.315943000	1.574166000	2.195401000
C	-7.180993000	3.229584000	1.439173000
H	-8.047744000	3.856890000	1.277493000
C	-5.915027000	3.700741000	1.132663000
H	-5.790155000	4.708033000	0.758378000
C	-4.781467000	2.913653000	1.346072000
C	-3.455941000	3.528472000	1.068315000
C	-2.439388000	3.553414000	2.028720000
H	-2.605028000	3.090398000	2.991718000
C	-1.231587000	4.185829000	1.771637000
H	-0.464736000	4.207809000	2.535444000
C	-1.016785000	4.818296000	0.551792000
H	-0.082365000	5.331948000	0.364176000
C	-2.020982000	4.808511000	-0.408245000
H	-1.878114000	5.318930000	-1.352441000
C	-3.225663000	4.164509000	-0.154281000
H	-4.002154000	4.158042000	-0.908637000
C	-6.464815000	-0.240473000	2.686056000
C	-7.088488000	-0.425444000	3.921662000
H	-7.364785000	0.439584000	4.511712000
C	-7.344972000	-1.704423000	4.400749000
H	-7.823786000	-1.831625000	5.363077000
C	-6.992577000	-2.816612000	3.644705000
H	-7.199116000	-3.812707000	4.013643000
C	-6.383518000	-2.642622000	2.406929000
H	-6.118714000	-3.502723000	1.805868000
C	-6.119267000	-1.363890000	1.934904000
H	-5.657878000	-1.227884000	0.966098000
C	1.451284000	0.712833000	2.780515000
H	0.574151000	1.326773000	2.982763000
H	2.165513000	1.311972000	2.217271000
H	1.906237000	0.427194000	3.739144000
C	0.220925000	2.431073000	-1.846520000
C	0.318030000	3.551094000	-2.760338000
H	-0.664380000	3.775078000	-3.176207000
H	1.003849000	3.310016000	-3.573584000
H	0.686086000	4.430146000	-2.231259000
Cu	0.000000000	0.000000000	0.000000000
N	1.054151000	-0.449257000	1.988146000
N	0.908752000	-1.816107000	-0.398764000
N	-1.627264000	-0.054771000	1.293881000
N	0.156632000	1.531300000	-1.136586000

101

E(B3LYP-D3BJ) = -3903.906219546237 Eh

$[Cu^{II}(1)(NCMe)(OH_2)]^{+}$ (S = 1/2)

C	2.482367000	0.445343000	-1.506645000
H	2.866384000	0.950879000	-0.624175000
H	1.900471000	1.152640000	-2.091449000

H	3.320155000	0.078119000	-2.105181000
C	1.028062000	-1.359323000	-2.275482000
H	1.768697000	-1.497420000	-3.067036000
H	0.688276000	-2.350527000	-1.966917000
C	-0.148128000	-0.557665000	-2.761493000
C	-0.545645000	-0.492047000	-4.083396000
H	0.018119000	-1.010443000	-4.847039000
C	-1.670536000	0.248636000	-4.413788000
H	-1.991308000	0.311937000	-5.443678000
C	-2.390338000	0.921016000	-3.424154000
C	-1.925109000	0.800203000	-2.118208000
H	-2.436329000	1.289357000	-1.301261000
N	-0.839962000	0.087194000	-1.802803000
C	-3.580017000	1.744451000	-3.740525000
C	-4.801406000	1.527954000	-3.071695000
C	-5.901908000	2.320031000	-3.403558000
H	-6.847593000	2.137634000	-2.911253000
C	-5.810227000	3.294871000	-4.384365000
H	-6.677294000	3.890097000	-4.637897000
C	-4.608364000	3.503345000	-5.042737000
H	-4.526622000	4.276809000	-5.794612000
C	-3.480843000	2.746701000	-4.726821000
C	-2.200737000	3.048085000	-5.418087000
C	-2.117736000	2.968519000	-6.809675000
H	-2.991335000	2.676243000	-7.378782000
C	-0.924852000	3.248544000	-7.465645000
H	-0.876420000	3.179490000	-8.544390000
C	0.200179000	3.622678000	-6.740100000
H	1.125530000	3.850832000	-7.252197000
C	0.123826000	3.722156000	-5.354628000
H	0.988609000	4.044794000	-4.788470000
C	-1.065931000	3.434131000	-4.700178000
H	-1.127975000	3.531017000	-3.623068000
C	-4.992332000	0.454449000	-2.062610000
C	-5.589971000	0.752093000	-0.834096000
H	-5.868433000	1.774850000	-0.613622000
C	-5.855173000	-0.251591000	0.089494000
H	-6.358539000	-0.004917000	1.016141000
C	-5.514019000	-1.570217000	-0.191730000
H	-5.751727000	-2.359777000	0.510285000
C	-4.910876000	-1.878139000	-1.406905000
H	-4.674617000	-2.906179000	-1.649543000
C	-4.657981000	-0.876038000	-2.334081000
H	-4.234229000	-1.134317000	-3.295275000
C	2.348224000	-1.600189000	-0.181516000
H	3.363157000	-1.791932000	-0.537599000
H	1.819012000	-2.555283000	-0.161419000
C	2.356842000	-1.001158000	1.197960000
C	3.409752000	-1.114220000	2.086221000
H	4.304319000	-1.652286000	1.803598000
C	3.308244000	-0.508160000	3.329252000
H	4.131159000	-0.564477000	4.027909000
C	2.154369000	0.194209000	3.680297000
C	1.131659000	0.236420000	2.741303000
H	0.212531000	0.766335000	2.948043000
N	1.239048000	-0.330592000	1.535758000
C	2.062036000	0.934370000	4.962155000
C	2.992502000	1.966477000	5.192125000
C	2.971113000	2.646892000	6.407117000
H	3.685905000	3.440403000	6.580177000
C	2.026460000	2.331648000	7.372952000
H	2.019149000	2.861332000	8.316220000
C	1.089797000	1.339763000	7.129833000
H	0.366916000	1.084604000	7.892807000
C	1.089231000	0.623620000	5.929519000
C	0.081340000	-0.451532000	5.747178000
C	0.437681000	-1.742015000	5.342054000
H	1.475255000	-1.974967000	5.146302000
C	-0.514828000	-2.748133000	5.243658000
H	-0.212074000	-3.746930000	4.956857000
C	-1.844238000	-2.487956000	5.559283000
H	-2.579671000	-3.280653000	5.514197000
C	-2.211803000	-1.211440000	5.972948000
H	-3.236589000	-1.006483000	6.257816000
C	-1.260170000	-0.201727000	6.056872000
H	-1.553629000	0.787025000	6.385981000
C	3.960395000	2.372752000	4.138672000
C	5.334707000	2.248491000	4.341141000
H	5.700130000	1.855578000	5.281673000
C	6.234199000	2.614131000	3.345149000
H	7.297276000	2.510218000	3.517976000
C	5.772199000	3.120085000	2.136261000
H	6.473630000	3.415484000	1.367335000
C	4.403786000	3.271099000	1.931938000
H	4.042971000	3.706268000	1.007672000
C	3.508384000	2.898084000	2.924810000
H	2.442964000	3.047100000	2.786679000
C	-2.556228000	-0.153043000	1.812344000
C	-3.623276000	-0.389906000	2.753783000
H	-3.185908000	-0.628456000	3.726318000
H	-4.231255000	-1.224264000	2.405679000
H	-4.248954000	0.499103000	2.835022000
Cu	0.000000000	0.000000000	0.000000000
N	1.623747000	-0.689128000	-1.097268000
N	-1.680798000	0.026596000	1.094916000
O	0.436695000	2.312862000	0.158706000
H	1.035791000	2.697310000	0.810257000
H	-0.094208000	3.040383000	-0.186218000

94
E(B3LYP-D3BJ) = -3845.342608074857 Eh
[Cu^{II}(η^1-$O_2^{\bullet -}$)(1)]$^+$ (isomer a^T) (*S* = 1)

C	2.431362000	-0.844510000	1.480023000
H	2.886276000	-1.541025000	2.193281000
H	3.032170000	0.067685000	1.500096000
C	2.477495000	-1.422854000	0.085610000
C	3.376441000	-2.407343000	-0.292019000
H	4.091592000	-2.789440000	0.424346000
C	3.315012000	-2.930368000	-1.576804000
H	3.977496000	-3.733242000	-1.871082000
C	2.355754000	-2.461013000	-2.469912000
C	1.560180000	-1.403297000	-2.040976000
H	0.828921000	-0.959742000	-2.701812000
C	2.046637000	-3.123038000	-3.759588000

C	0.740123000	-3.624088000	-3.939357000
C	0.410433000	-4.254037000	-5.136481000
H	-0.591095000	-4.640978000	-5.271506000
C	1.364585000	-4.416907000	-6.131679000
H	1.101611000	-4.914302000	-7.055998000
C	2.654601000	-3.945152000	-5.942636000
H	3.393679000	-4.059064000	-6.724611000
C	3.012850000	-3.284703000	-4.765743000
C	4.389915000	-2.754347000	-4.613670000
C	4.616108000	-1.407931000	-4.316670000
H	3.773933000	-0.737021000	-4.208452000
C	5.908373000	-0.918565000	-4.185722000
H	6.066855000	0.130637000	-3.971669000
C	6.997266000	-1.769384000	-4.343001000
H	8.004803000	-1.388132000	-4.240575000
C	6.784797000	-3.109712000	-4.643857000
H	7.626829000	-3.777431000	-4.772456000
C	5.490934000	-3.597211000	-4.782700000
H	5.329493000	-4.643162000	-5.011960000
C	-0.267499000	-3.528529000	-2.845606000
C	-1.419544000	-2.754933000	-2.993292000
H	-1.598545000	-2.242819000	-3.930373000
C	-2.313249000	-2.604712000	-1.938113000
H	-3.190728000	-1.983848000	-2.066906000
C	-2.078221000	-3.240869000	-0.725436000
H	-2.772086000	-3.125931000	0.095266000
C	-0.950432000	-4.041677000	-0.579059000
H	-0.777005000	-4.568849000	0.351124000
C	-0.053650000	-4.182795000	-1.628997000
H	0.824049000	-4.804533000	-1.509541000
C	0.238829000	-1.634721000	2.251297000
H	0.479112000	-1.983936000	3.261942000
H	0.462479000	-2.448976000	1.556320000
C	-1.229963000	-1.297267000	2.146687000
C	-2.190815000	-1.792149000	3.010419000
H	-1.903742000	-2.450912000	3.819054000
C	-3.516167000	-1.408543000	2.844889000
H	-4.274467000	-1.765316000	3.528163000
C	-3.863421000	-0.524996000	1.826026000
C	-2.843929000	-0.123558000	0.965362000
H	-3.043016000	0.528884000	0.127032000
C	-5.219387000	0.060021000	1.690431000
C	-6.359259000	-0.754766000	1.577020000
C	-7.615953000	-0.155889000	1.486991000
H	-8.490168000	-0.786143000	1.390389000
C	-7.747160000	1.224552000	1.481545000
H	-8.727761000	1.674607000	1.399262000
C	-6.620786000	2.028430000	1.580248000
H	-6.719091000	3.105950000	1.593946000
C	-5.352716000	1.463404000	1.699923000
C	-4.169130000	2.349070000	1.866203000
C	-3.387263000	2.290033000	3.023263000
H	-3.671732000	1.617450000	3.822171000
C	-2.259068000	3.087142000	3.157826000
H	-1.675580000	3.042748000	4.069049000
C	-1.890741000	3.955317000	2.134416000
H	-1.012295000	4.578996000	2.238627000
C	-2.670835000	4.034225000	0.986698000
H	-2.396608000	4.712308000	0.189102000
C	-3.805913000	3.242286000	0.857647000
H	-4.402871000	3.296928000	-0.043959000
C	-6.256929000	-2.234426000	1.512778000
C	-6.905910000	-3.031333000	2.457583000
H	-7.474960000	-2.560136000	3.249374000
C	-6.816313000	-4.417040000	2.396192000
H	-7.320326000	-5.021092000	3.139520000
C	-6.086826000	-5.025681000	1.381348000
H	-6.023741000	-6.104691000	1.328917000
C	-5.449917000	-4.241250000	0.425655000
H	-4.895699000	-4.707460000	-0.378722000
C	-5.532447000	-2.857235000	0.494372000
H	-5.049674000	-2.248663000	-0.259354000
C	1.039077000	0.568129000	2.904961000
H	0.008576000	0.856128000	3.105859000
H	1.576168000	1.445520000	2.548953000
H	1.499346000	0.222469000	3.838464000
Cu	0.000000000	0.000000000	0.000000000
N	1.064127000	-0.474110000	1.870181000
N	1.604256000	-0.916367000	-0.801137000
N	-1.576380000	-0.492227000	1.127180000
O	-0.651617000	0.906834000	-1.640482000
O	-1.821516000	1.354015000	-1.658077000

94
E(B3LYP-D3BJ) = -3845.342096995557 Eh
[Cu^{II}(η^1-$O_2^{\bullet -}$)(1)]$^+$ (isomer b^T) (*S* = 1)

C	2.420455000	-0.899894000	1.472580000
H	2.827541000	-1.427350000	2.342478000
H	3.199447000	-0.219393000	1.121009000
C	2.104806000	-1.880682000	0.369348000
C	2.684170000	-3.136578000	0.293785000
H	3.397450000	-3.446544000	1.045947000
C	2.305104000	-4.005126000	-0.721770000
H	2.713069000	-5.005935000	-0.765163000
C	1.353948000	-3.602091000	-1.653359000
C	0.895575000	-2.291203000	-1.562091000
H	0.197963000	-1.896703000	-2.287611000
C	0.708083000	-4.518847000	-2.622418000
C	-0.690940000	-4.676939000	-2.535111000
C	-1.340260000	-5.511460000	-3.440609000
H	-2.413164000	-5.634313000	-3.370989000
C	-0.615958000	-6.207304000	-4.399324000
H	-1.127967000	-6.857778000	-5.096210000
C	0.762898000	-6.075597000	-4.461260000
H	1.325955000	-6.611552000	-5.213853000
C	1.443594000	-5.228079000	-3.585064000
C	2.916642000	-5.089118000	-3.694198000
C	3.517198000	-3.839866000	-3.868445000
H	2.897307000	-2.956119000	-3.945718000
C	4.897053000	-3.725337000	-3.967125000
H	5.346303000	-2.751989000	-4.117297000
C	5.700284000	-4.857938000	-3.887216000
H	6.775990000	-4.768247000	-3.963373000
C	5.113436000	-6.106913000	-3.721194000

H	5.730998000	-6.993865000	-3.663277000
C	3.731734000	-6.221715000	-3.630174000
H	3.277905000	-7.195464000	-3.494346000
C	-1.460275000	-4.003914000	-1.450519000
C	-2.404368000	-3.017671000	-1.737516000
H	-2.606258000	-2.759012000	-2.769247000
C	-3.052569000	-2.337245000	-0.712096000
H	-3.768433000	-1.562578000	-0.955643000
C	-2.778685000	-2.643541000	0.615231000
H	-3.280472000	-2.115169000	1.413691000
C	-1.863358000	-3.648114000	0.911924000
H	-1.668105000	-3.916919000	1.942821000
C	-1.210767000	-4.320728000	-0.111782000
H	-0.495500000	-5.098121000	0.123141000
C	0.277816000	-0.793734000	2.668456000
H	0.613473000	-0.846945000	3.710455000
H	0.191817000	-1.818070000	2.294636000
C	-1.076083000	-0.129087000	2.584228000
C	-1.945989000	-0.018451000	3.654437000
H	-1.668757000	-0.415244000	4.621940000
C	-3.159217000	0.634818000	3.477627000
H	-3.838224000	0.753828000	4.310769000
C	-3.486207000	1.176678000	2.236759000
C	-2.580204000	0.965864000	1.199776000
H	-2.785176000	1.322458000	0.200266000
C	-4.686044000	2.019280000	2.017658000
C	-5.974895000	1.546162000	2.321028000
C	-7.068614000	2.392124000	2.135006000
H	-8.060356000	2.022052000	2.359110000
C	-6.900036000	3.673451000	1.632407000
H	-7.759698000	4.313198000	1.482079000
C	-5.629388000	4.134059000	1.319242000
H	-5.490908000	5.138740000	0.941950000
C	-4.511863000	3.326196000	1.518829000
C	-3.155686000	3.864746000	1.227413000
C	-2.200935000	3.993469000	2.239911000
H	-2.467691000	3.737343000	3.257146000
C	-0.921650000	4.450096000	1.954214000
H	-0.200178000	4.562065000	2.754020000
C	-0.573046000	4.781613000	0.648568000
H	0.423647000	5.139037000	0.424286000
C	-1.520108000	4.674373000	-0.363181000
H	-1.261077000	4.939594000	-1.379936000
C	-2.803946000	4.226712000	-0.073642000
H	-3.535169000	4.133224000	-0.866824000
C	-6.207336000	0.157585000	2.790944000
C	-6.873836000	-0.083466000	3.993747000
H	-7.204363000	0.754036000	4.595451000
C	-7.103142000	-1.383856000	4.427938000
H	-7.616747000	-1.553908000	5.365343000
C	-6.678073000	-2.462151000	3.660577000
H	-6.862935000	-3.474347000	3.995609000
C	-6.025379000	-2.233414000	2.453789000
H	-5.708881000	-3.067236000	1.840364000
C	-5.791403000	-0.934184000	2.025445000
H	-5.301379000	-0.757867000	1.076821000
C	1.627953000	1.212600000	2.408183000
H	0.732546000	1.808517000	2.576378000
H	2.272387000	1.751404000	1.715875000
H	2.155007000	1.087146000	3.361763000
Cu	0.000000000	0.000000000	0.000000000
N	1.252673000	-0.080963000	1.822308000
N	1.238813000	-1.466557000	-0.573323000
N	-1.417987000	0.343679000	1.374247000
O	-1.038870000	0.633490000	-1.580710000
O	-0.798797000	0.159572000	-2.713757000

94

E(B3LYP-D3BJ) = -3845.341228385783 Eh

$[Cu^{II}(\eta^1\text{-}O_2^{\bullet-})(1)]^+$ (isomer c^T) (S = 1)

Cu	0.000000000	0.000000000	0.000000000
C	-1.166520000	0.134175000	2.670378000
H	-1.865402000	0.587235000	3.381821000
H	-0.297744000	-0.202175000	3.240739000
C	-1.801166000	-1.053968000	1.988950000
C	-2.886128000	-1.736383000	2.515144000
H	-3.309092000	-1.433480000	3.463780000
C	-3.447460000	-2.780688000	1.792107000
H	-4.318043000	-3.300304000	2.168587000
C	-2.920120000	-3.122145000	0.549908000
C	-1.776923000	-2.439973000	0.140494000
H	-1.294811000	-2.690714000	-0.793890000
C	-3.579133000	-4.061802000	-0.388209000
C	-3.956887000	-3.570973000	-1.655112000
C	-4.558313000	-4.432390000	-2.569305000
H	-4.852156000	-4.051770000	-3.538756000
C	-4.813963000	-5.753329000	-2.228344000
H	-5.286697000	-6.414406000	-2.942739000
C	-4.471851000	-6.224781000	-0.970189000
H	-4.664685000	-7.256196000	-0.706180000
C	-3.845589000	-5.395653000	-0.037964000
C	-3.473479000	-5.942790000	1.290195000
C	-2.156804000	-5.894109000	1.754170000
H	-1.386063000	-5.463569000	1.128146000
C	-1.825060000	-6.413300000	2.998224000
H	-0.797435000	-6.382075000	3.336912000
C	-2.806427000	-6.984091000	3.801719000
H	-2.547863000	-7.388471000	4.771584000
C	-4.118795000	-7.043254000	3.347794000
H	-4.887325000	-7.490091000	3.965140000
C	-4.448599000	-6.530871000	2.099044000
H	-5.472851000	-6.573764000	1.750240000
C	-3.770879000	-2.134426000	-2.001032000
C	-4.422765000	-1.140906000	-1.264637000
H	-5.098804000	-1.428302000	-0.469999000
C	-4.212879000	0.202728000	-1.542063000
H	-4.738409000	0.958553000	-0.971327000
C	-3.345063000	0.578759000	-2.561430000
H	-3.177314000	1.625312000	-2.772807000
C	-2.708859000	-0.400353000	-3.314488000
H	-2.038990000	-0.119687000	-4.117470000
C	-2.925669000	-1.746789000	-3.041482000
H	-2.416863000	-2.505909000	-3.622459000
C	-1.794028000	1.871439000	1.054761000

H	-2.155450000	2.664502000	1.717501000
H	-2.619225000	1.174766000	0.886971000
C	-1.364286000	2.438149000	-0.275554000
C	-1.762720000	3.672964000	-0.755901000
H	-2.418500000	4.299726000	-0.166503000
C	-1.275566000	4.113366000	-1.980919000
H	-1.545055000	5.092927000	-2.350677000
C	-0.398677000	3.315070000	-2.712158000
C	-0.113965000	2.054884000	-2.188881000
H	0.525453000	1.365596000	-2.720473000
C	0.314894000	3.792561000	-3.921626000
C	1.724783000	3.732301000	-3.936780000
C	2.415408000	4.190004000	-5.056697000
H	3.496611000	4.147136000	-5.060422000
C	1.733109000	4.728721000	-6.137778000
H	2.280739000	5.090497000	-6.998057000
C	0.348862000	4.804026000	-6.114880000
H	-0.188131000	5.207905000	-6.962995000
C	-0.376609000	4.329708000	-5.021690000
C	-1.859700000	4.375045000	-5.068026000
C	-2.613358000	3.202371000	-4.992134000
H	-2.103241000	2.251496000	-4.909584000
C	-3.999764000	3.243686000	-5.049784000
H	-4.567264000	2.323267000	-5.001493000
C	-4.655471000	4.463168000	-5.179762000
H	-5.736059000	4.497391000	-5.225703000
C	-3.915204000	5.636759000	-5.265266000
H	-4.418432000	6.588730000	-5.374613000
C	-2.526927000	5.592462000	-5.215401000
H	-1.952983000	6.508424000	-5.280922000
C	2.488482000	3.218730000	-2.767160000
C	2.405948000	3.849963000	-1.523173000
H	1.817647000	4.753212000	-1.424834000
C	3.068138000	3.329311000	-0.419687000
H	3.008809000	3.840676000	0.533131000
C	3.820296000	2.164962000	-0.540482000
H	4.331580000	1.753217000	0.319790000
C	3.928474000	1.542591000	-1.778394000
H	4.515763000	0.639780000	-1.881621000
C	3.274827000	2.071440000	-2.884939000
H	3.349050000	1.575303000	-3.844669000
C	0.321552000	2.027718000	2.252539000
H	-0.096666000	2.623257000	3.071938000
H	0.681246000	2.697907000	1.473998000
N	-1.244018000	-1.431677000	0.827493000
N	-0.566562000	1.644457000	-1.007883000
N	-0.684928000	1.117594000	1.683111000
H	1.163619000	1.448104000	2.625756000
O	1.254370000	-0.909629000	-1.192097000
O	2.055151000	-1.092661000	-0.224065000

94

E(B3LYP-D3BJ) = -3845.341289932772 Eh

$[Cu^{II}(\eta^1\text{-}O_2{}^{\bullet -})(\mathbf{1})]^+$ (isomer d^T) (S = 1)

C	2.386287000	-0.791603000	1.527747000
H	2.861840000	-1.480659000	2.234218000
H	2.893255000	0.170760000	1.627437000
C	2.557144000	-1.288748000	0.111883000
C	3.550207000	-2.179402000	-0.262697000
H	4.253031000	-2.547175000	0.473092000
C	3.603983000	-2.624587000	-1.577098000
H	4.345819000	-3.352117000	-1.877620000
C	2.662159000	-2.175019000	-2.498234000
C	1.755742000	-1.215787000	-2.056975000
H	1.027324000	-0.794697000	-2.735349000
C	2.494430000	-2.760555000	-3.849322000
C	1.253494000	-3.357227000	-4.154498000
C	1.054776000	-3.910050000	-5.416983000
H	0.103818000	-4.372058000	-5.647798000
C	2.075463000	-3.900384000	-6.357657000
H	1.913988000	-4.336471000	-7.334699000
C	3.303107000	-3.336485000	-6.045894000
H	4.094713000	-3.318506000	-6.783224000
C	3.529752000	-2.752067000	-4.798206000
C	4.845634000	-2.130097000	-4.510156000
C	4.943484000	-0.804250000	-4.080062000
H	4.045254000	-0.210713000	-3.970696000
C	6.181195000	-0.234391000	-3.815333000
H	6.240019000	0.798401000	-3.496539000
C	7.343049000	-0.982465000	-3.972562000
H	8.308027000	-0.538578000	-3.765684000
C	7.258914000	-2.299576000	-4.408627000
H	8.158656000	-2.886776000	-4.539303000
C	6.019951000	-2.867512000	-4.678956000
H	5.958519000	-3.896362000	-5.010948000
C	0.179443000	-3.443298000	-3.125460000
C	-1.032926000	-2.772859000	-3.293045000
H	-1.206127000	-2.207934000	-4.200430000
C	-1.999678000	-2.794580000	-2.293215000
H	-2.927054000	-2.254412000	-2.436231000
C	-1.775799000	-3.500457000	-1.117491000
H	-2.525694000	-3.518282000	-0.338935000
C	-0.583991000	-4.198482000	-0.954016000
H	-0.414634000	-4.778786000	-0.055244000
C	0.384288000	-4.168479000	-1.948056000
H	1.311395000	-4.710414000	-1.814647000
C	0.236294000	-1.831913000	2.098893000
H	0.450414000	-2.225446000	3.098238000
H	0.583557000	-2.566794000	1.367905000
C	-1.247373000	-1.626381000	1.912224000
C	-2.217943000	-2.233630000	2.689087000
H	-1.932477000	-2.900935000	3.491261000
C	-3.556467000	-1.944776000	2.450388000
H	-4.325007000	-2.382347000	3.072468000
C	-3.909327000	-1.052876000	1.440340000
C	-2.872350000	-0.545431000	0.660382000
H	-3.073129000	0.118506000	-0.167873000
C	-5.292510000	-0.553955000	1.247149000
C	-6.371214000	-1.434382000	1.055127000
C	-7.657952000	-0.911878000	0.921838000
H	-8.485161000	-1.591670000	0.765496000
C	-7.876586000	0.457235000	0.949175000
H	-8.879138000	0.847320000	0.832587000
C	-6.809657000	1.326818000	1.124881000

H	-6.976743000	2.395229000	1.163712000
C	-5.515801000	0.838024000	1.290432000
C	-4.395929000	1.785759000	1.541264000
C	-3.687635000	1.750818000	2.745208000
H	-3.994030000	1.061168000	3.521204000
C	-2.599965000	2.588155000	2.951981000
H	-2.073413000	2.561130000	3.897958000
C	-2.197357000	3.470579000	1.954219000
H	-1.346878000	4.121142000	2.111647000
C	-2.908189000	3.528843000	0.761030000
H	-2.605943000	4.216812000	-0.017514000
C	-4.005942000	2.699706000	0.561256000
H	-4.549154000	2.738097000	-0.374794000
C	-6.168630000	-2.901373000	0.955065000
C	-6.814817000	-3.769325000	1.837054000
H	-7.459918000	-3.362634000	2.605938000
C	-6.623387000	-5.143049000	1.745465000
H	-7.126183000	-5.802589000	2.440898000
C	-5.792164000	-5.668893000	0.763071000
H	-5.648355000	-6.738760000	0.687859000
C	-5.157534000	-4.813838000	-0.131787000
H	-4.525021000	-5.216308000	-0.912557000
C	-5.342925000	-3.441819000	-0.033052000
H	-4.861627000	-2.778988000	-0.740496000
C	0.793077000	0.370811000	2.969671000
H	-0.268840000	0.545569000	3.133422000
H	1.263258000	1.320436000	2.721240000
H	1.236409000	-0.017239000	3.894208000
Cu	0.000000000	0.000000000	0.000000000
N	0.967218000	-0.570067000	1.853027000
N	1.693360000	-0.800671000	-0.793101000
N	-1.592508000	-0.812738000	0.901281000
O	-0.177186000	2.097353000	-1.386913000
O	-0.824204000	1.015493000	-1.467142000

94
E(B3LYP-D3BJ) = -3845.342512651142 Eh
[Cu^{II}(η^1-$O_2^{\bullet -}$)(1)]$^+$ (isomer e^T) (*S* = 1)

C	1.272386000	1.664992000	-2.030100000
H	1.193605000	2.609752000	-1.486320000
H	1.568180000	1.909983000	-3.056250000
C	2.346232000	0.829787000	-1.369530000
C	3.671884000	0.899353000	-1.758950000
H	3.952816000	1.511984000	-2.605170000
C	4.629024000	0.190408000	-1.049350000
H	5.669932000	0.246662000	-1.335650000
C	4.249927000	-0.595418000	0.036560000
C	2.893742000	-0.633915000	0.346110000
H	2.527718000	-1.230035000	1.170770000
C	5.234201000	-1.342071000	0.856480000
C	6.306768000	-0.648969000	1.451040000
C	7.232960000	-1.354238000	2.218260000
H	8.049318000	-0.814322000	2.679330000
C	7.094092000	-2.718616000	2.421320000
H	7.815230000	-3.253284000	3.025430000
C	6.029984000	-3.396784000	1.848460000
H	5.930586000	-4.465287000	1.985590000
C	5.096252000	-2.728754000	1.054400000
C	4.019041000	-3.526713000	0.414920000
C	3.202532000	-4.349954000	1.194020000
H	3.326835000	-4.355392000	2.269320000
C	2.234886000	-5.153582000	0.604890000
H	1.617613000	-5.791136000	1.224810000
C	2.068714000	-5.149635000	-0.774920000
H	1.326719000	-5.789498000	-1.234330000
C	2.871214000	-4.328923000	-1.560700000
H	2.762884000	-4.335155000	-2.638090000
C	3.836201000	-3.523659000	-0.970700000
H	4.475315000	-2.909277000	-1.591120000
C	6.461434000	0.823050000	1.318570000
C	5.464724000	1.694997000	1.763600000
H	4.568543000	1.291404000	2.217460000
C	5.622138000	3.069133000	1.647890000
H	4.847602000	3.731742000	2.012810000
C	6.777907000	3.595219000	1.080000000
H	6.901636000	4.666554000	0.990620000
C	7.779532000	2.737365000	0.641300000
H	8.685192000	3.138439000	0.204970000
C	7.624156000	1.361691000	0.764810000
H	8.405428000	0.695963000	0.419530000
C	-1.193885000	1.879080000	-1.899610000
H	-1.524527000	2.211646000	-2.889540000
H	-0.910586000	2.770443000	-1.334860000
C	-2.324181000	1.181954000	-1.179160000
C	-3.652928000	1.316355000	-1.539010000
H	-3.924701000	1.954487000	-2.369270000
C	-4.621885000	0.588247000	-0.858820000
H	-5.658582000	0.645233000	-1.161420000
C	-4.247962000	-0.259780000	0.178300000
C	-2.900800000	-0.264304000	0.535060000
H	-2.550435000	-0.861709000	1.363230000
C	-5.171170000	-1.235743000	0.806460000
C	-4.821855000	-2.601546000	0.747620000
C	-5.667368000	-3.549166000	1.316710000
H	-5.397403000	-4.595859000	1.266210000
C	-6.859756000	-3.159865000	1.911980000
H	-7.515068000	-3.903071000	2.346880000
C	-7.213083000	-1.819803000	1.948470000
H	-8.135197000	-1.514818000	2.425260000
C	-6.377208000	-0.840245000	1.408810000
C	-6.775342000	0.585952000	1.499550000
C	-5.935173000	1.537120000	2.083770000
H	-4.980944000	1.227499000	2.489980000
C	-6.323428000	2.866961000	2.170860000
H	-5.667272000	3.588932000	2.639910000
C	-7.557277000	3.269439000	1.670710000
H	-7.860314000	4.305981000	1.739640000
C	-8.403996000	2.330979000	1.092370000
H	-9.367478000	2.635144000	0.704280000
C	-8.017329000	0.998889000	1.011100000
H	-8.676914000	0.271629000	0.554380000
C	-3.579653000	-3.025468000	0.042850000
C	-2.492701000	-3.539045000	0.750020000
H	-2.577190000	-3.689230000	1.819060000

C	-1.287999000	-3.798594000	0.106610000
H	-0.440094000	-4.157834000	0.673420000
C	-1.162398000	-3.572984000	-1.258790000
H	-0.220767000	-3.769818000	-1.753190000
C	-2.258050000	-3.112512000	-1.983430000
H	-2.181857000	-2.971864000	-3.054580000
C	-3.454198000	-2.837197000	-1.336560000
H	-4.298810000	-2.458689000	-1.897720000
C	-0.170339000	-0.030465000	-3.015540000
H	-1.088005000	-0.591982000	-2.851000000
H	0.667092000	-0.724747000	-2.963740000
H	-0.197627000	0.420419000	-4.014790000
O	0.692016000	-1.694785000	2.244140000
Cu	0.000000000	0.000000000	0.000000000
N	-0.025971000	0.994748000	-1.974180000
N	1.972981000	0.060087000	-0.328840000
N	-1.968314000	0.411156000	-0.134510000
O	-0.246639000	-1.048478000	1.722580000

94
E(B3LYP-D3BJ) = -3845.334447201181 Eh
$[Cu^{II}(\eta^1\text{-}O_2^{\bullet-})(1)]^+$ (isomer a^{BS}) (M_S = 0)

C	2.423048000	-0.833711000	1.465481000
H	2.864274000	-1.525925000	2.190165000
H	3.023110000	0.078602000	1.489666000
C	2.480372000	-1.415675000	0.074531000
C	3.383477000	-2.394425000	-0.305589000
H	4.101728000	-2.775561000	0.408119000
C	3.320685000	-2.914083000	-1.592237000
H	3.985456000	-3.713938000	-1.889545000
C	2.357572000	-2.447433000	-2.482636000
C	1.557909000	-1.393081000	-2.051524000
H	0.822425000	-0.947966000	-2.706493000
C	2.045968000	-3.111641000	-3.770469000
C	0.740093000	-3.616679000	-3.943296000
C	0.407098000	-4.250042000	-5.137526000
H	-0.594040000	-4.639629000	-5.267628000
C	1.357533000	-4.412218000	-6.136425000
H	1.091890000	-4.911938000	-7.058718000
C	2.647169000	-3.936783000	-5.953926000
H	3.383197000	-4.050418000	-6.738802000
C	3.008656000	-3.273011000	-4.779966000
C	4.384931000	-2.739053000	-4.633955000
C	4.608653000	-1.391395000	-4.340738000
H	3.765187000	-0.722198000	-4.231768000
C	5.900202000	-0.898556000	-4.215502000
H	6.056805000	0.151596000	-4.004801000
C	6.990622000	-1.747050000	-4.374748000
H	7.997578000	-1.363040000	-4.277047000
C	6.780483000	-3.088590000	-4.671813000
H	7.623742000	-3.754433000	-4.802094000
C	5.487369000	-3.579649000	-4.804982000
H	5.327712000	-4.626446000	-5.031611000
C	-0.261832000	-3.522012000	-2.844003000
C	-1.409999000	-2.740971000	-2.981119000
H	-1.589989000	-2.219909000	-3.912923000
C	-2.297191000	-2.591849000	-1.920523000
H	-3.169719000	-1.962699000	-2.040904000
C	-2.059762000	-3.236804000	-0.713081000
H	-2.749885000	-3.124235000	0.111217000
C	-0.935102000	-4.044164000	-0.576741000
H	-0.760105000	-4.578398000	0.349182000
C	-0.044402000	-4.183944000	-1.632124000
H	0.830676000	-4.810976000	-1.520861000
C	0.231208000	-1.621314000	2.250090000
H	0.484886000	-1.945711000	3.265106000
H	0.454433000	-2.447557000	1.569350000
C	-1.237359000	-1.286866000	2.149684000
C	-2.200591000	-1.783610000	3.008625000
H	-1.917935000	-2.445976000	3.815830000
C	-3.524989000	-1.396808000	2.838513000
H	-4.286473000	-1.755358000	3.517302000
C	-3.867365000	-0.509590000	1.821313000
C	-2.845109000	-0.105235000	0.964840000
H	-3.033611000	0.547913000	0.124632000
C	-5.221938000	0.076723000	1.680051000
C	-6.361965000	-0.736280000	1.556766000
C	-7.616799000	-0.134928000	1.458938000
H	-8.491471000	-0.763278000	1.354396000
C	-7.745283000	1.245818000	1.455049000
H	-8.724428000	1.697744000	1.365940000
C	-6.618379000	2.047827000	1.563371000
H	-6.714854000	3.125501000	1.577425000
C	-5.352461000	1.480205000	1.691082000
C	-4.166754000	2.361285000	1.867827000
C	-3.402957000	2.305767000	3.037051000
H	-3.704243000	1.641469000	3.836790000
C	-2.271356000	3.096283000	3.182400000
H	-1.702359000	3.055850000	4.102982000
C	-1.881481000	3.953246000	2.157457000
H	-1.000546000	4.572029000	2.269946000
C	-2.643191000	4.027748000	0.997209000
H	-2.351311000	4.695744000	0.197446000
C	-3.781952000	3.243100000	0.857478000
H	-4.363053000	3.292765000	-0.054614000
C	-6.260034000	-2.215801000	1.489418000
C	-6.915699000	-3.015169000	2.427440000
H	-7.490576000	-2.546049000	3.216250000
C	-6.825395000	-4.400702000	2.363172000
H	-7.334683000	-5.006722000	3.101282000
C	-6.088612000	-5.006652000	1.352005000
H	-6.025161000	-6.085524000	1.297218000
C	-5.445006000	-4.219711000	0.402870000
H	-4.885219000	-4.683701000	-0.398923000
C	-5.528141000	-2.835883000	0.474514000
H	-5.040121000	-2.225498000	-0.274347000
C	1.013542000	0.606868000	2.847600000
H	-0.018613000	0.890375000	3.044290000
H	1.543232000	1.479346000	2.469547000
H	1.479580000	0.284205000	3.785634000
Cu	0.000000000	0.000000000	0.000000000
N	1.046893000	-0.461524000	1.836014000
N	1.604760000	-0.911143000	-0.810398000
N	-1.579785000	-0.477374000	1.132656000

O	-0.657705000	0.798957000	-1.637774000
O	-1.840558000	1.265141000	-1.641604000

94

E(B3LYP-D3BJ) = -3845.333903451944 Eh

[$Cu^{II}(\eta^1$-$O_2^{\bullet-})(1)]^+$ (isomer b^{BS}) (M_S = 0)

C	2.399669000	-0.916328000	1.458880000
H	2.787462000	-1.438274000	2.339756000
H	3.187075000	-0.241393000	1.116194000
C	2.090838000	-1.897043000	0.355392000
C	2.671383000	-3.151132000	0.271346000
H	3.384812000	-3.467167000	1.020796000
C	2.291476000	-4.011065000	-0.751919000
H	2.698970000	-5.011714000	-0.803204000
C	1.340552000	-3.600937000	-1.680554000
C	0.881591000	-2.290411000	-1.581260000
H	0.187351000	-1.882917000	-2.302764000
C	0.692563000	-4.511904000	-2.653621000
C	-0.705665000	-4.671977000	-2.559674000
C	-1.358918000	-5.503051000	-3.465175000
H	-2.431369000	-5.626909000	-3.390833000
C	-0.638548000	-6.193079000	-4.431210000
H	-1.153309000	-6.840648000	-5.128774000
C	0.739680000	-6.058564000	-4.500515000
H	1.299103000	-6.589254000	-5.259521000
C	1.424140000	-5.214281000	-3.624053000
C	2.895848000	-5.070099000	-3.742050000
C	3.490817000	-3.817294000	-3.910454000
H	2.867537000	-2.935021000	-3.976611000
C	4.869472000	-3.697225000	-4.019037000
H	5.314121000	-2.721140000	-4.165102000
C	5.677176000	-4.827659000	-3.954991000
H	6.751922000	-4.733685000	-4.039145000
C	5.095834000	-6.080010000	-3.794886000
H	5.716792000	-6.965325000	-3.749495000
C	3.715302000	-6.200352000	-3.693933000
H	3.265982000	-7.176876000	-3.563104000
C	-1.467669000	-4.003197000	-1.466878000
C	-2.392351000	-2.995293000	-1.741174000
H	-2.586227000	-2.716002000	-2.768930000
C	-3.027785000	-2.316376000	-0.707037000
H	-3.724877000	-1.522139000	-0.940957000
C	-2.761266000	-2.646236000	0.616127000
H	-3.254780000	-2.119585000	1.421030000
C	-1.865238000	-3.672044000	0.899932000
H	-1.676189000	-3.959166000	1.927111000
C	-1.224421000	-4.342771000	-0.132627000
H	-0.522899000	-5.135712000	0.091901000
C	0.263660000	-0.780029000	2.669036000
H	0.618466000	-0.803530000	3.705093000
H	0.173633000	-1.813687000	2.323187000
C	-1.088595000	-0.114231000	2.583192000
C	-1.967316000	-0.002133000	3.645486000
H	-1.700243000	-0.397283000	4.616482000
C	-3.179252000	0.650427000	3.454316000
H	-3.866582000	0.769950000	4.280488000
C	-3.495926000	1.188862000	2.209036000
C	-2.580094000	0.978478000	1.180467000
H	-2.772181000	1.329166000	0.176424000
C	-4.696580000	2.025956000	1.974432000
C	-5.986506000	1.549622000	2.267641000
C	-7.081421000	2.389518000	2.062192000
H	-8.074167000	2.017192000	2.277961000
C	-6.912316000	3.667265000	1.550750000
H	-7.772970000	4.301829000	1.384914000
C	-5.640093000	4.131088000	1.248457000
H	-5.501397000	5.133049000	0.864132000
C	-4.521778000	3.329551000	1.467289000
C	-3.163851000	3.870053000	1.187641000
C	-2.224435000	4.014308000	2.212304000
H	-2.505658000	3.771289000	3.228906000
C	-0.941990000	4.470106000	1.939185000
H	-0.232727000	4.595313000	2.747965000
C	-0.575190000	4.784343000	0.634180000
H	0.423857000	5.141151000	0.419449000
C	-1.507048000	4.661125000	-0.389804000
H	-1.233302000	4.911159000	-1.406483000
C	-2.794168000	4.215079000	-0.112966000
H	-3.512882000	4.107632000	-0.915681000
C	-6.217649000	0.163953000	2.746756000
C	-6.893727000	-0.069548000	3.945722000
H	-7.233637000	0.771652000	4.536943000
C	-7.120741000	-1.367101000	4.389433000
H	-7.642013000	-1.531260000	5.323652000
C	-6.683722000	-2.450136000	3.635507000
H	-6.866893000	-3.460195000	3.977846000
C	-6.021519000	-2.229006000	2.432490000
H	-5.695889000	-3.066593000	1.829069000
C	-5.789932000	-0.932507000	1.994547000
H	-5.292786000	-0.762603000	1.048493000
C	1.606358000	1.223565000	2.334226000
H	0.712370000	1.822042000	2.498833000
H	2.241264000	1.744107000	1.619727000
H	2.145676000	1.120183000	3.282742000
Cu	0.000000000	0.000000000	0.000000000
N	1.226821000	-0.088945000	1.787038000
N	1.225988000	-1.476326000	-0.584879000
N	-1.420150000	0.357984000	1.370988000
O	-1.056620000	0.587955000	-1.519219000
O	-0.771899000	0.136052000	-2.672234000

94

E(B3LYP-D3BJ) = -3845.333388469245 Eh

[$Cu^{II}(\eta^1$-$O_2^{\bullet-})(1)]^+$ (isomer c^{BS}) (M_S = 0)

C	2.293205000	-1.076022000	1.419056000
H	2.703043000	-1.588287000	2.295427000
H	3.059272000	-0.385290000	1.059858000
C	1.966056000	-2.064597000	0.327673000
C	2.536034000	-3.323993000	0.241520000
H	3.267941000	-3.640185000	0.972883000
C	2.122853000	-4.186680000	-0.765679000
H	2.526264000	-5.188441000	-0.825377000
C	1.143297000	-3.779927000	-1.667052000
C	0.690171000	-2.467814000	-1.558879000

H	-0.038216000	-2.076365000	-2.255266000
C	0.479913000	-4.698685000	-2.622487000
C	-0.913899000	-4.876251000	-2.501426000
C	-1.573749000	-5.717265000	-3.394024000
H	-2.642541000	-5.856402000	-3.297207000
C	-0.864635000	-6.399528000	-4.373099000
H	-1.384749000	-7.055546000	-5.058690000
C	0.509962000	-6.246686000	-4.470038000
H	1.061414000	-6.770911000	-5.239270000
C	1.200426000	-5.392724000	-3.607944000
C	2.667405000	-5.230224000	-3.759107000
C	3.242871000	-3.971014000	-3.946147000
H	2.607834000	-3.096359000	-4.000055000
C	4.616905000	-3.834520000	-4.088216000
H	5.045880000	-2.853613000	-4.248013000
C	5.439621000	-4.954817000	-4.039626000
H	6.510794000	-4.848067000	-4.149817000
C	4.877645000	-6.213532000	-3.861258000
H	5.510291000	-7.091057000	-3.827778000
C	3.501525000	-6.350244000	-3.726673000
H	3.067368000	-7.331654000	-3.581904000
C	-1.671278000	-4.216435000	-1.401785000
C	-2.672567000	-3.284005000	-1.675882000
H	-2.920290000	-3.058530000	-2.705565000
C	-3.326413000	-2.620963000	-0.642740000
H	-4.091526000	-1.891875000	-0.877514000
C	-2.999045000	-2.889366000	0.680451000
H	-3.505532000	-2.374011000	1.484169000
C	-2.024810000	-3.840108000	0.965393000
H	-1.788224000	-4.082372000	1.994284000
C	-1.368376000	-4.497015000	-0.065972000
H	-0.612353000	-5.237138000	0.161095000
C	0.130152000	-1.031447000	2.583875000
H	0.460371000	-1.109499000	3.624361000
H	0.071669000	-2.042211000	2.171537000
C	-1.233129000	-0.393756000	2.493072000
C	-2.134262000	-0.322172000	3.539882000
H	-1.879637000	-0.734362000	4.507099000
C	-3.351019000	0.320714000	3.341837000
H	-4.050562000	0.419164000	4.160407000
C	-3.653469000	0.880870000	2.102612000
C	-2.717977000	0.696591000	1.085396000
H	-2.899026000	1.067753000	0.087373000
C	-4.843208000	1.734854000	1.868456000
C	-6.142225000	1.286815000	2.167276000
C	-7.220625000	2.148179000	1.962090000
H	-8.219861000	1.795911000	2.181500000
C	-7.028330000	3.421312000	1.447955000
H	-7.876920000	4.071954000	1.282225000
C	-5.747542000	3.860075000	1.144869000
H	-5.589143000	4.859033000	0.760384000
C	-4.645003000	3.037133000	1.362673000
C	-3.276986000	3.552792000	1.083680000
C	-2.338902000	3.687735000	2.110430000
H	-2.631155000	3.468357000	3.129368000
C	-1.041300000	4.097055000	1.834304000
H	-0.331284000	4.213814000	2.643801000
C	-0.658437000	4.372335000	0.525420000
H	0.355083000	4.681419000	0.306008000
C	-1.590558000	4.265534000	-0.499894000
H	-1.300930000	4.479551000	-1.519914000
C	-2.892623000	3.868558000	-0.220466000
H	-3.611465000	3.769485000	-1.024348000
C	-6.405602000	-0.089246000	2.656635000
C	-7.086755000	-0.296263000	3.857854000
H	-7.403716000	0.557921000	4.443114000
C	-7.347508000	-1.584022000	4.310930000
H	-7.872061000	-1.727764000	5.246674000
C	-6.939968000	-2.683609000	3.564261000
H	-7.149317000	-3.686088000	3.913734000
C	-6.272909000	-2.488555000	2.359635000
H	-5.968592000	-3.338768000	1.762864000
C	-6.007311000	-1.201597000	1.912529000
H	-5.505515000	-1.050218000	0.966025000
C	1.479357000	0.994845000	2.435540000
H	0.580195000	1.574776000	2.634533000
H	2.124375000	1.573217000	1.777162000
H	2.002039000	0.808113000	3.379900000
Cu	0.000000000	0.000000000	0.000000000
N	1.108671000	-0.267956000	1.772369000
N	1.078111000	-1.645408000	-0.587049000
N	-1.548199000	0.097808000	1.284462000
O	0.256575000	1.606599000	-1.629525000
O	-0.706263000	0.751283000	-1.629867000

100

E(B3LYP-D3BJ) = -3978.107455000660 Eh

$[Cu^{II}(\eta^1\text{-}O_2^{\bullet-})(1)(NCMe)]^+$ (*S* = 1)

C	2.332353000	-1.171942000	1.443646000
H	2.809283000	-1.872558000	2.140938000
H	2.927459000	-0.255069000	1.455797000
C	2.341142000	-1.747120000	0.049084000
C	3.214525000	-2.751236000	-0.338398000
H	3.935149000	-3.142043000	0.367801000
C	3.123704000	-3.277516000	-1.619369000
H	3.772079000	-4.087729000	-1.924817000
C	2.153753000	-2.796398000	-2.492922000
C	1.372876000	-1.732723000	-2.047002000
H	0.628210000	-1.292050000	-2.694285000
C	1.837848000	-3.444017000	-3.789688000
C	0.533705000	-3.947435000	-3.974190000
C	0.201210000	-4.561131000	-5.179209000
H	-0.800138000	-4.948283000	-5.315333000
C	1.151299000	-4.706632000	-6.180470000
H	0.886828000	-5.191559000	-7.111037000
C	2.439988000	-4.232480000	-5.988643000
H	3.176846000	-4.331844000	-6.774759000
C	2.799937000	-3.588122000	-4.803241000
C	4.176879000	-3.055782000	-4.654254000
C	4.402486000	-1.713567000	-4.338851000
H	3.558999000	-1.048693000	-4.207288000
C	5.694544000	-1.222012000	-4.215264000
H	5.852075000	-0.175967000	-3.985193000
C	6.784714000	-2.065976000	-4.398751000

H	7.792175000	-1.683009000	-4.301646000
C	6.572943000	-3.402149000	-4.717554000
H	7.415425000	-4.065283000	-4.865825000
C	5.278993000	-3.891654000	-4.848520000
H	5.118164000	-4.934623000	-5.091337000
C	-0.470776000	-3.862912000	-2.878506000
C	-1.618519000	-3.082496000	-3.017239000
H	-1.796001000	-2.559927000	-3.948718000
C	-2.504436000	-2.931854000	-1.956701000
H	-3.374864000	-2.300066000	-2.076564000
C	-2.265651000	-3.572719000	-0.748162000
H	-2.951299000	-3.451311000	0.077669000
C	-1.143796000	-4.383165000	-0.612094000
H	-0.966622000	-4.911834000	0.316542000
C	-0.254310000	-4.526149000	-1.667564000
H	0.621761000	-5.151563000	-1.555531000
C	0.118796000	-1.984352000	2.095131000
H	0.320459000	-2.444193000	3.070409000
H	0.342963000	-2.729232000	1.327618000
C	-1.338067000	-1.611816000	1.983181000
C	-2.322917000	-2.157863000	2.790744000
H	-2.060496000	-2.884928000	3.547735000
C	-3.637502000	-1.739156000	2.637030000
H	-4.413682000	-2.134809000	3.277522000
C	-3.948599000	-0.770652000	1.685316000
C	-2.904936000	-0.322601000	0.877640000
H	-3.084623000	0.401058000	0.094101000
C	-5.292837000	-0.151422000	1.577816000
C	-6.442588000	-0.936357000	1.378341000
C	-7.690248000	-0.316871000	1.303066000
H	-8.567834000	-0.928530000	1.140482000
C	-7.807251000	1.060643000	1.400761000
H	-8.780113000	1.529278000	1.331635000
C	-6.674976000	1.835932000	1.595534000
H	-6.764788000	2.908614000	1.704810000
C	-5.413788000	1.248547000	1.701507000
C	-4.252925000	2.127849000	2.002748000
C	-3.438367000	1.896128000	3.115779000
H	-3.645298000	1.051873000	3.759222000
C	-2.385364000	2.747195000	3.417344000
H	-1.779662000	2.562111000	4.295397000
C	-2.129024000	3.853996000	2.614806000
H	-1.323114000	4.531303000	2.868233000
C	-2.934399000	4.099249000	1.509583000
H	-2.759846000	4.968820000	0.888359000
C	-3.985119000	3.242019000	1.204941000
H	-4.604949000	3.434604000	0.338645000
C	-6.372803000	-2.411162000	1.215967000
C	-7.029266000	-3.250794000	2.117958000
H	-7.569271000	-2.817671000	2.950875000
C	-6.985845000	-4.630827000	1.960469000
H	-7.494699000	-5.268810000	2.671518000
C	-6.296716000	-5.190063000	0.890333000
H	-6.269191000	-6.264283000	0.762597000
C	-5.653089000	-4.361604000	-0.022114000
H	-5.125397000	-4.787680000	-0.865302000
C	-5.687587000	-2.983461000	0.143834000
H	-5.197064000	-2.339390000	-0.574131000
C	0.963250000	0.104237000	3.000783000
H	-0.063289000	0.392644000	3.222408000
H	1.521700000	1.003752000	2.747760000
H	1.403849000	-0.350781000	3.897371000
Cu	0.000000000	0.000000000	0.000000000
N	0.975244000	-0.813150000	1.861188000
N	1.452441000	-1.231933000	-0.815816000
N	-1.645552000	-0.725214000	1.025250000
O	-0.933156000	0.277765000	-1.847072000
O	-1.951784000	0.996787000	-1.867321000
N	0.315747000	2.015983000	0.179200000
C	0.294129000	3.153557000	0.041393000
C	0.271564000	4.588294000	-0.156761000
H	0.208412000	5.096347000	0.805091000
H	-0.596510000	4.856496000	-0.759239000
H	1.177696000	4.906592000	-0.673251000

133
E(B3LYP-D3BJ) = -4785.488965065003 Eh
$[Cu^{II}(\eta^1\text{-}O_2^{\bullet-})(2)]^+$ (S = 1)

Cu	0.000000000	0.000000000	0.000000000
C	-1.424928000	0.317257000	2.547744000
H	-2.128260000	0.780956000	3.249647000
H	-0.573458000	-0.044356000	3.129426000
C	-2.068675000	-0.861341000	1.858286000
C	-3.170736000	-1.525823000	2.371721000
H	-3.602825000	-1.212554000	3.312970000
C	-3.738533000	-2.563452000	1.644089000
H	-4.624625000	-3.064500000	2.009080000
C	-3.199039000	-2.919489000	0.410802000
C	-2.036685000	-2.255826000	0.017600000
H	-1.535270000	-2.516913000	-0.903908000
C	-3.880813000	-3.843063000	-0.530050000
C	-4.221435000	-3.353716000	-1.808030000
C	-4.864629000	-4.193243000	-2.714394000
H	-5.125853000	-3.809791000	-3.692060000
C	-5.203120000	-5.490430000	-2.357347000
H	-5.710733000	-6.132349000	-3.065280000
C	-4.897307000	-5.961077000	-1.089551000
H	-5.152964000	-6.974387000	-0.808958000
C	-4.227102000	-5.155700000	-0.168225000
C	-3.876808000	-5.719864000	1.159562000
C	-2.543684000	-5.855417000	1.547779000
H	-1.762176000	-5.548336000	0.865647000
C	-2.214906000	-6.401113000	2.781288000
H	-1.174917000	-6.505913000	3.061779000
C	-3.218948000	-6.814479000	3.649864000
H	-2.964695000	-7.239671000	4.611944000
C	-4.551036000	-6.689069000	3.271629000
H	-5.337845000	-7.013790000	3.940268000
C	-4.877476000	-6.151024000	2.032413000
H	-5.915660000	-6.053777000	1.739864000
C	-3.950113000	-1.938989000	-2.181260000
C	-4.564903000	-0.893366000	-1.487102000
H	-5.271933000	-1.123556000	-0.700751000
C	-4.274444000	0.428294000	-1.794436000

H	-4.769293000	1.227755000	-1.256753000
C	-3.361231000	0.727664000	-2.799184000
H	-3.129190000	1.756307000	-3.033566000
C	-2.759606000	-0.304390000	-3.507667000
H	-2.049059000	-0.083757000	-4.293405000
C	-3.058445000	-1.628174000	-3.207667000
H	-2.573951000	-2.429683000	-3.751004000
C	-1.950756000	2.020466000	0.851659000
H	-2.325362000	2.868282000	1.437731000
H	-2.791316000	1.341698000	0.688121000
C	-1.441837000	2.495330000	-0.488614000
C	-1.839736000	3.694496000	-1.057358000
H	-2.554710000	4.325986000	-0.546467000
C	-1.276513000	4.095198000	-2.261749000
H	-1.544257000	5.047102000	-2.699838000
C	-0.314837000	3.296166000	-2.873462000
C	-0.040859000	2.066894000	-2.272755000
H	0.666388000	1.378513000	-2.714885000
C	0.506172000	3.763540000	-4.018296000
C	1.904570000	3.822905000	-3.847255000
C	2.705678000	4.264367000	-4.897733000
H	3.777798000	4.309759000	-4.757973000
C	2.137135000	4.678561000	-6.093498000
H	2.766912000	5.029231000	-6.900581000
C	0.760043000	4.648782000	-6.250797000
H	0.312377000	4.962269000	-7.184725000
C	-0.069372000	4.184048000	-5.229149000
C	-1.533634000	4.124655000	-5.465176000
C	-2.216432000	2.908766000	-5.417610000
H	-1.664365000	2.002952000	-5.205094000
C	-3.580952000	2.850090000	-5.666504000
H	-4.092676000	1.896999000	-5.634866000
C	-4.286331000	4.011998000	-5.959509000
H	-5.350158000	3.968038000	-6.152874000
C	-3.616062000	5.228856000	-6.014047000
H	-4.157394000	6.136782000	-6.246918000
C	-2.248308000	5.283838000	-5.774113000
H	-1.728127000	6.232675000	-5.817382000
C	2.532714000	3.470267000	-2.544897000
C	2.247380000	4.212779000	-1.395562000
H	1.590226000	5.069571000	-1.468143000
C	2.797788000	3.861740000	-0.170942000
H	2.579995000	4.456716000	0.707646000
C	3.638108000	2.757792000	-0.073368000
H	4.061346000	2.478676000	0.880761000
C	3.937944000	2.022772000	-1.212530000
H	4.586700000	1.159433000	-1.149379000
C	3.396857000	2.381048000	-2.441144000
H	3.616991000	1.791715000	-3.322028000
C	0.193130000	2.103897000	2.052392000
H	-0.047466000	2.595872000	3.002288000
H	0.364469000	2.889026000	1.312297000
C	1.456346000	1.289087000	2.190070000
C	2.377768000	1.504891000	3.202428000
H	2.212103000	2.290626000	3.927763000
C	3.484806000	0.673108000	3.301377000
H	4.189934000	0.797943000	4.111875000

C	3.653192000	-0.363119000	2.387540000
C	2.723652000	-0.446274000	1.350258000
H	2.826126000	-1.198320000	0.580647000
C	4.663538000	-1.435422000	2.567560000
C	4.203060000	-2.764314000	2.671735000
C	5.121233000	-3.797385000	2.844495000
H	4.760047000	-4.814131000	2.927519000
C	6.477356000	-3.525062000	2.952445000
H	7.182725000	-4.332994000	3.096175000
C	6.926795000	-2.215397000	2.885516000
H	7.984033000	-1.998672000	2.963629000
C	6.036402000	-1.159573000	2.684295000
C	6.572185000	0.221038000	2.583701000
C	6.390885000	0.979791000	1.427117000
H	5.853581000	0.549102000	0.593122000
C	6.912952000	2.262468000	1.329757000
H	6.771104000	2.832826000	0.421008000
C	7.619655000	2.809775000	2.394975000
H	8.026784000	3.809677000	2.320966000
C	7.810543000	2.061669000	3.551116000
H	8.363708000	2.479054000	4.382623000
C	7.294924000	0.774449000	3.642695000
H	7.443231000	0.193888000	4.544733000
C	2.746839000	-3.071907000	2.664455000
C	2.178458000	-3.835756000	1.645362000
H	2.809676000	-4.220536000	0.854162000
C	0.809298000	-4.074574000	1.620190000
H	0.385308000	-4.653360000	0.810038000
C	-0.009106000	-3.567254000	2.620872000
H	-1.072575000	-3.755613000	2.603153000
C	0.551635000	-2.826248000	3.654899000
H	-0.075473000	-2.450274000	4.454035000
C	1.916864000	-2.579059000	3.675138000
H	2.349544000	-1.999994000	4.480606000
O	1.625742000	-0.766180000	-2.060809000
N	-1.502102000	-1.253187000	0.708038000
N	-0.577459000	1.685501000	-1.117250000
N	-0.911259000	1.274011000	1.564729000
N	1.653705000	0.339317000	1.264463000
O	0.715069000	-1.257470000	-1.334399000

131
E(B3LYP-D3BJ) = -4355.588338317102 Eh
[Cu^{II}(OAr-Me)(1)]$^{+}$ (conformer 1) (S = 1/2)

C	2.288795000	-1.107689000	1.620946000
H	2.649242000	-1.832640000	2.361051000
H	2.950294000	-0.240035000	1.680901000
C	2.382612000	-1.694152000	0.233757000
C	3.358252000	-2.613331000	-0.123361000
H	4.093999000	-2.932560000	0.603101000
C	3.351299000	-3.141841000	-1.405565000
H	4.080757000	-3.886208000	-1.696174000
C	2.356087000	-2.762540000	-2.302054000
C	1.473674000	-1.771820000	-1.889358000
H	0.710308000	-1.389073000	-2.552623000
C	2.108877000	-3.464538000	-3.584582000
C	0.916720000	-4.207305000	-3.692282000

C	0.631635000	-4.868191000	-4.884649000
H	-0.282410000	-5.441741000	-4.966659000
C	1.527341000	-4.821108000	-5.944371000
H	1.301818000	-5.342174000	-6.865608000
C	2.712226000	-4.110842000	-5.824522000
H	3.403525000	-4.065010000	-6.655652000
C	3.017435000	-3.415866000	-4.652043000
C	4.277648000	-2.638370000	-4.562403000
C	4.271823000	-1.284595000	-4.215906000
H	3.331110000	-0.785861000	-4.024284000
C	5.457751000	-0.567259000	-4.140448000
H	5.433608000	0.484128000	-3.883758000
C	6.671195000	-1.192530000	-4.406325000
H	7.595965000	-0.633649000	-4.346668000
C	6.689006000	-2.536831000	-4.759066000
H	7.628758000	-3.029992000	-4.971517000
C	5.501074000	-3.252853000	-4.839601000
H	5.519603000	-4.302085000	-5.106475000
C	-0.010469000	-4.316084000	-2.531852000
C	-1.303348000	-3.799229000	-2.597239000
H	-1.642053000	-3.331945000	-3.512872000
C	-2.139818000	-3.840721000	-1.487714000
H	-3.132653000	-3.420834000	-1.554550000
C	-1.702319000	-4.403225000	-0.296016000
H	-2.358144000	-4.429142000	0.564748000
C	-0.422544000	-4.946411000	-0.227221000
H	-0.078388000	-5.416153000	0.685913000
C	0.411837000	-4.904578000	-1.335338000
H	1.404323000	-5.330497000	-1.276277000
C	-0.011856000	-1.738127000	2.206263000
H	0.153112000	-2.170402000	3.200187000
H	0.153251000	-2.528703000	1.469112000
C	-1.430835000	-1.240863000	2.078315000
C	-2.472447000	-1.675712000	2.877858000
H	-2.289733000	-2.399870000	3.660365000
C	-3.747506000	-1.168350000	2.665114000
H	-4.572301000	-1.503385000	3.278350000
C	-3.962716000	-0.212402000	1.674753000
C	-2.857619000	0.173255000	0.920002000
H	-2.949314000	0.909298000	0.134316000
C	-5.288204000	0.388508000	1.395838000
C	-6.401163000	-0.431488000	1.128913000
C	-7.626358000	0.162612000	0.824935000
H	-8.475766000	-0.472422000	0.610744000
C	-7.753956000	1.541676000	0.764987000
H	-8.708452000	1.987499000	0.517730000
C	-6.659718000	2.348223000	1.035702000
H	-6.762365000	3.425250000	1.027010000
C	-5.425186000	1.790535000	1.368484000
C	-4.313609000	2.701991000	1.743463000
C	-3.677960000	2.588042000	2.983783000
H	-3.992566000	1.815914000	3.673531000
C	-2.671404000	3.470910000	3.348601000
H	-2.204017000	3.381907000	4.321159000
C	-2.282314000	4.484688000	2.478565000
H	-1.505638000	5.181237000	2.766755000
C	-2.906541000	4.605732000	1.243770000
H	-2.607636000	5.387288000	0.559201000
C	-3.912495000	3.720730000	0.878348000
H	-4.381376000	3.805633000	-0.092893000
C	-6.321307000	-1.915723000	1.150309000
C	-7.139212000	-2.638190000	2.021870000
H	-7.808793000	-2.104402000	2.684699000
C	-7.090856000	-4.026506000	2.056660000
H	-7.727488000	-4.569224000	2.743369000
C	-6.232345000	-4.714840000	1.208284000
H	-6.199483000	-5.796319000	1.226409000
C	-5.423989000	-4.005766000	0.326267000
H	-4.772291000	-4.540647000	-0.351686000
C	-5.463544000	-2.618015000	0.300828000
H	-4.836906000	-2.071688000	-0.391474000
C	0.937921000	0.332157000	3.032213000
H	-0.067450000	0.721299000	3.181311000
H	1.593073000	1.163599000	2.783657000
H	1.281149000	-0.125215000	3.968179000
Cu	0.000000000	0.000000000	0.000000000
N	0.933784000	-0.640480000	1.932267000
N	1.487876000	-1.258926000	-0.661042000
N	-1.643747000	-0.335867000	1.107547000
O	-0.416633000	0.778999000	-1.695411000
C	-1.096830000	1.869035000	-1.948688000
C	-0.589358000	3.162639000	-1.569985000
C	-2.328723000	1.779707000	-2.688069000
C	-1.381285000	4.268567000	-1.820214000
C	-3.069151000	2.937280000	-2.869744000
C	-2.634268000	4.183329000	-2.426105000
H	-1.028798000	5.251150000	-1.546211000
H	-4.009955000	2.893347000	-3.397069000
C	-3.473825000	5.413603000	-2.609778000
H	-2.886035000	6.238022000	-3.018760000
H	-4.314490000	5.229879000	-3.278297000
H	-3.881104000	5.759255000	-1.654069000
C	0.797181000	3.354683000	-0.929030000
C	1.890376000	2.584260000	-1.697440000
C	0.760619000	2.917848000	0.544239000
H	0.008997000	3.475412000	1.103108000
H	0.511496000	1.859662000	0.656439000
H	1.735110000	3.073507000	1.012928000
H	1.727850000	1.511886000	-1.686664000
H	2.866062000	2.797603000	-1.255175000
H	1.918556000	2.908587000	-2.739227000
C	-2.778202000	0.466429000	-3.345777000
C	-3.102324000	-0.608750000	-2.298323000
C	-1.666501000	-0.039831000	-4.288606000
H	-1.452914000	0.702096000	-5.059870000
H	-0.747722000	-0.242501000	-3.746032000
H	-1.991612000	-0.956275000	-4.785825000
H	-2.239151000	-0.858862000	-1.689545000
H	-3.438890000	-1.515611000	-2.803239000
H	-3.913333000	-0.275760000	-1.648037000
C	1.225776000	4.833299000	-0.929391000
H	0.585135000	5.453477000	-0.301412000
H	2.238588000	4.909714000	-0.531646000
H	1.231602000	5.250706000	-1.936983000

C	-4.043359000	0.651939000	-4.198317000
H	-3.896782000	1.380576000	-4.996669000
H	-4.297286000	-0.300802000	-4.665157000
H	-4.901631000	0.961432000	-3.599322000

131

E(B3LYP-D3BJ) = -4355.592132087560 Eh

[Cu^{II}(OAr-Me)(1)]$^+$ (conformer 2) (*S* = 1/2)

C	2.284267000	-1.236510000	1.393277000
H	2.651018000	-2.004669000	2.083527000
H	2.993287000	-0.406316000	1.441405000
C	2.283296000	-1.760970000	-0.022629000
C	3.217175000	-2.690584000	-0.452732000
H	3.916016000	-3.122748000	0.251199000
C	3.258744000	-3.034934000	-1.793249000
H	3.998633000	-3.737368000	-2.151961000
C	2.349481000	-2.471039000	-2.687072000
C	1.423612000	-1.574242000	-2.167156000
H	0.691777000	-1.104325000	-2.807911000
C	2.401892000	-2.772050000	-4.141342000
C	1.319516000	-3.379257000	-4.809611000
C	1.434307000	-3.630117000	-6.181180000
H	0.620370000	-4.125575000	-6.691851000
C	2.583574000	-3.304854000	-6.880552000
H	2.650954000	-3.515098000	-7.939898000
C	3.650684000	-2.722434000	-6.216696000
H	4.549849000	-2.456515000	-6.756192000
C	3.575787000	-2.447097000	-4.851644000
C	4.749664000	-1.813968000	-4.196380000
C	4.639987000	-0.610304000	-3.496338000
H	3.679100000	-0.119006000	-3.431895000
C	5.752532000	-0.039039000	-2.894368000
H	5.653294000	0.895760000	-2.358728000
C	6.994225000	-0.659762000	-2.981867000
H	7.860588000	-0.213133000	-2.511738000
C	7.117277000	-1.851943000	-3.684926000
H	8.079950000	-2.340458000	-3.762615000
C	6.003827000	-2.422077000	-4.289885000
H	6.101101000	-3.355864000	-4.829322000
C	0.074660000	-3.827742000	-4.133761000
C	-1.158541000	-3.654218000	-4.771717000
H	-1.197015000	-3.122155000	-5.712755000
C	-2.331974000	-4.142750000	-4.215627000
H	-3.272413000	-3.989469000	-4.728524000
C	-2.301421000	-4.811404000	-2.998623000
H	-3.214803000	-5.198092000	-2.565903000
C	-1.085946000	-4.989173000	-2.348060000
H	-1.047162000	-5.528218000	-1.409901000
C	0.088627000	-4.506674000	-2.910315000
H	1.027597000	-4.692583000	-2.409721000
C	0.035894000	-1.781493000	2.203662000
H	0.267464000	-2.155790000	3.206895000
H	0.157255000	-2.612704000	1.504077000
C	-1.385882000	-1.280259000	2.131416000
C	-2.400429000	-1.675465000	2.985531000
H	-2.198821000	-2.370060000	3.790059000
C	-3.677679000	-1.162049000	2.793565000
H	-4.484996000	-1.459282000	3.448845000
C	-3.920224000	-0.250519000	1.768038000
C	-2.843347000	0.085372000	0.954138000
H	-2.947661000	0.774207000	0.129371000
C	-5.246002000	0.371387000	1.540700000
C	-6.379916000	-0.432404000	1.323500000
C	-7.621814000	0.179600000	1.152981000
H	-8.492081000	-0.439414000	0.978702000
C	-7.739335000	1.561441000	1.159094000
H	-8.707991000	2.022471000	1.017039000
C	-6.613210000	2.353077000	1.331498000
H	-6.700230000	3.431668000	1.334493000
C	-5.360822000	1.774670000	1.531568000
C	-4.171334000	2.645760000	1.722613000
C	-3.406118000	2.586191000	2.890038000
H	-3.710242000	1.925432000	3.691454000
C	-2.266101000	3.365937000	3.030595000
H	-1.692513000	3.318467000	3.947858000
C	-1.870510000	4.217403000	2.004146000
H	-0.979765000	4.822583000	2.111130000
C	-2.633651000	4.296896000	0.845548000
H	-2.336396000	4.960419000	0.043798000
C	-3.778739000	3.521650000	0.708978000
H	-4.360941000	3.572761000	-0.202408000
C	-6.271633000	-1.908622000	1.206935000
C	-7.048389000	-2.748730000	2.006437000
H	-7.725998000	-2.315143000	2.731326000
C	-6.945582000	-4.130360000	1.891311000
H	-7.550028000	-4.768184000	2.523200000
C	-6.070615000	-4.691709000	0.968543000
H	-5.993881000	-5.767393000	0.877530000
C	-5.303176000	-3.863080000	0.156120000
H	-4.630062000	-4.289039000	-0.577087000
C	-5.402688000	-2.484587000	0.275379000
H	-4.811064000	-1.845952000	-0.366703000
C	1.095745000	0.301862000	2.861309000
H	0.110515000	0.702955000	3.092227000
H	1.725896000	1.117301000	2.512642000
H	1.529713000	-0.122123000	3.774269000
Cu	0.000000000	0.000000000	0.000000000
N	0.975687000	-0.713876000	1.803395000
N	1.400681000	-1.221536000	-0.877134000
N	-1.628780000	-0.420088000	1.133225000
O	-0.843218000	1.143088000	-1.248221000
C	-0.300288000	1.215155000	-2.442715000
C	-0.994947000	0.625208000	-3.555614000
C	0.978509000	1.816229000	-2.635718000
C	-0.312830000	0.486952000	-4.749277000
C	1.598186000	1.624950000	-3.868809000
C	1.003169000	0.934066000	-4.916031000
H	-0.792197000	0.007434000	-5.590090000
H	2.585874000	2.034639000	-4.035145000
C	1.732582000	0.682963000	-6.203667000
H	2.735566000	1.108125000	-6.183282000
H	1.826589000	-0.389080000	-6.393042000
H	1.197287000	1.114890000	-7.052903000
C	1.720089000	2.698199000	-1.609676000

C	3.125120000	2.126557000	-1.343298000
C	1.040640000	2.890765000	-0.246147000
H	0.006718000	3.211463000	-0.326579000
H	1.055947000	1.976876000	0.355635000
H	1.600653000	3.642906000	0.314617000
H	3.064605000	1.111701000	-0.944917000
H	3.651616000	2.749957000	-0.617454000
H	3.729701000	2.092450000	-2.247387000
C	-2.443126000	0.139334000	-3.403664000
C	-2.504955000	-1.070874000	-2.452062000
C	-3.037547000	-0.302456000	-4.749210000
H	-3.014467000	0.502410000	-5.485478000
H	-2.517721000	-1.165838000	-5.162807000
H	-4.080149000	-0.587761000	-4.601575000
H	-1.850791000	-1.869620000	-2.796540000
H	-3.522009000	-1.468628000	-2.424931000
H	-2.215973000	-0.801605000	-1.443431000
C	-3.330520000	1.284320000	-2.874612000
H	-3.364370000	2.103644000	-3.595501000
H	-4.351873000	0.924541000	-2.729664000
H	-2.962823000	1.678917000	-1.931284000
C	1.857019000	4.107022000	-2.230179000
H	2.381175000	4.082417000	-3.184596000
H	0.872764000	4.546069000	-2.400932000
H	2.412212000	4.762235000	-1.555781000

131
E(B3LYP-D3BJ) = -4355.582644013265 Eh
[Cu^{II}(OAr-Me)(1)]$^+$ (conformer 3) (*S* = 1/2)

C	2.309594000	-1.300389000	1.204128000
H	2.005748000	-2.315016000	0.935898000
H	3.117687000	-1.383780000	1.938486000
C	2.798787000	-0.597890000	-0.036715000
C	4.142957000	-0.461255000	-0.330006000
H	4.887411000	-0.838566000	0.357931000
C	4.515346000	0.187195000	-1.496125000
H	5.560835000	0.341097000	-1.725361000
C	3.540888000	0.657264000	-2.371374000
C	2.209273000	0.458374000	-2.014003000
H	1.405729000	0.803141000	-2.652159000
C	3.896225000	1.427810000	-3.589685000
C	3.595863000	0.938602000	-4.873723000
C	3.893540000	1.736897000	-5.981934000
H	3.681765000	1.356403000	-6.972001000
C	4.502274000	2.971945000	-5.832741000
H	4.731958000	3.570978000	-6.704229000
C	4.843305000	3.426425000	-4.567855000
H	5.332662000	4.383438000	-4.444234000
C	4.545643000	2.668473000	-3.436209000
C	4.951414000	3.184224000	-2.100796000
C	4.021679000	3.439736000	-1.091070000
H	2.970402000	3.282075000	-1.282232000
C	4.431187000	3.908382000	0.149650000
H	3.693735000	4.117993000	0.914535000
C	5.780964000	4.126151000	0.405507000
H	6.101146000	4.491610000	1.372495000
C	6.715306000	3.884499000	-0.594522000
H	7.767451000	4.056649000	-0.408044000
C	6.302419000	3.422105000	-1.838546000
H	7.033938000	3.228666000	-2.613144000
C	3.065515000	-0.426658000	-5.114227000
C	2.042595000	-0.630933000	-6.042348000
H	1.577573000	0.223415000	-6.512517000
C	1.615577000	-1.911741000	-6.362516000
H	0.823676000	-2.046798000	-7.087698000
C	2.199856000	-3.016438000	-5.757190000
H	1.870601000	-4.015635000	-6.010370000
C	3.214469000	-2.828970000	-4.825596000
H	3.687711000	-3.683687000	-4.359534000
C	3.643131000	-1.547607000	-4.508556000
H	4.462935000	-1.419760000	-3.815897000
C	0.233109000	-1.447914000	2.530734000
H	0.577408000	-1.567406000	3.564140000
H	0.218148000	-2.441597000	2.076856000
C	-1.166782000	-0.880375000	2.500569000
C	-2.128582000	-1.181936000	3.450502000
H	-1.884651000	-1.820007000	4.289723000
C	-3.404172000	-0.657227000	3.302755000
H	-4.175195000	-0.885518000	4.026478000
C	-3.691534000	0.185297000	2.231235000
C	-2.671420000	0.423015000	1.318985000
H	-2.826569000	1.028369000	0.437667000
C	-5.018165000	0.826554000	2.058735000
C	-6.150675000	0.037178000	1.796583000
C	-7.394096000	0.656734000	1.671950000
H	-8.265163000	0.049342000	1.463933000
C	-7.511024000	2.035418000	1.771572000
H	-8.480230000	2.504766000	1.664818000
C	-6.385733000	2.814177000	1.998433000
H	-6.475482000	3.889310000	2.082009000
C	-5.130984000	2.224870000	2.154589000
C	-3.944748000	3.079671000	2.419290000
C	-3.152997000	2.894973000	3.556284000
H	-3.424789000	2.134789000	4.276162000
C	-2.035295000	3.686814000	3.779771000
H	-1.443860000	3.541094000	4.675015000
C	-1.687404000	4.678095000	2.867283000
H	-0.818218000	5.298987000	3.042121000
C	-2.474824000	4.879619000	1.740472000
H	-2.216823000	5.653823000	1.029412000
C	-3.596856000	4.089819000	1.521479000
H	-4.200074000	4.240220000	0.635850000
C	-6.033893000	-1.430401000	1.601473000
C	-6.760784000	-2.316526000	2.397513000
H	-7.409075000	-1.925849000	3.172007000
C	-6.646161000	-3.690018000	2.214591000
H	-7.211978000	-4.364382000	2.844373000
C	-5.809800000	-4.196253000	1.226657000
H	-5.724495000	-5.265416000	1.082309000
C	-5.092934000	-3.321127000	0.416788000
H	-4.454774000	-3.704212000	-0.369044000
C	-5.203750000	-1.950971000	0.604381000
H	-4.655408000	-1.275161000	-0.038508000
C	1.521902000	0.618105000	2.475235000

H	0.627533000	1.163943000	2.769264000
H	2.121676000	1.257954000	1.829611000
H	2.104220000	0.374091000	3.371130000
Cu	0.000000000	0.000000000	0.000000000
N	1.140913000	-0.603205000	1.746366000
N	1.850165000	-0.123155000	-0.865575000
N	-1.456885000	-0.104338000	1.450141000
O	-1.300712000	0.535207000	-1.274627000
C	-1.186460000	0.544655000	-2.586507000
C	-1.025380000	1.804175000	-3.259123000
C	-1.293736000	-0.660895000	-3.342491000
C	-0.976805000	1.808918000	-4.643013000
C	-1.251375000	-0.559630000	-4.729774000
C	-1.101118000	0.644527000	-5.400387000
H	-0.867983000	2.742680000	-5.173793000
H	-1.351464000	-1.456228000	-5.325509000
C	-1.130195000	0.708522000	-6.901329000
H	-0.864395000	-0.248944000	-7.349561000
H	-2.131001000	0.966874000	-7.259833000
H	-0.448416000	1.470347000	-7.283924000
C	-1.519826000	-2.083616000	-2.778994000
C	-2.911887000	-2.566074000	-3.241140000
C	-1.491583000	-2.251619000	-1.253687000
H	-0.498029000	-2.036044000	-0.845862000
H	-2.224499000	-1.633241000	-0.747871000
H	-1.699072000	-3.298108000	-1.018685000
H	-3.695971000	-1.924623000	-2.834239000
H	-3.090387000	-3.587125000	-2.895922000
H	-3.005229000	-2.558657000	-4.326180000
C	-1.034814000	3.133455000	-2.483367000
C	-2.413667000	3.316955000	-1.821257000
C	-0.807733000	4.340044000	-3.409239000
H	0.148296000	4.278135000	-3.932462000
H	-1.601040000	4.446067000	-4.149805000
H	-0.797344000	5.251481000	-2.809804000
H	-3.203760000	3.291684000	-2.573624000
H	-2.456942000	4.282578000	-1.314417000
H	-2.608796000	2.537669000	-1.090307000
C	0.071123000	3.203790000	-1.415345000
H	1.050587000	3.106735000	-1.883608000
H	0.037856000	4.172149000	-0.912557000
H	-0.034748000	2.432601000	-0.656793000
C	-0.434520000	-3.024234000	-3.338435000
H	-0.455419000	-3.087672000	-4.423568000
H	0.561336000	-2.679434000	-3.053620000
H	-0.576126000	-4.033226000	-2.944470000

70
E(B3LYP-D3BJ) = -2997.447492762093 Eh
$[Cu^{II}(\eta^1\text{-}O_2^{\bullet-})(TMGMP)]^+$ (S = 1)

C	1.983701000	1.870902000	1.046440000
C	2.906892000	2.907804000	1.050675000
H	3.486480000	3.141227000	1.931126000
C	3.075723000	3.645849000	-0.116268000
H	3.788388000	4.460216000	-0.135706000
C	2.338169000	3.350724000	-1.257936000
H	2.470249000	3.934439000	-2.156598000
C	1.429182000	2.302192000	-1.202946000
C	1.734017000	0.870608000	2.193037000
C	0.439693000	1.901014000	-2.319022000
Cu	-0.041064000	0.173854000	-0.013098000
N	0.382458000	0.315398000	1.948824000
N	1.299156000	1.621751000	-0.067411000
N	0.016204000	0.517952000	-1.999154000
O	-1.400842000	-1.217946000	-0.047422000
O	-1.094310000	-2.252594000	0.675042000
C	-0.471029000	-0.077380000	2.870933000
N	-0.143011000	-0.769555000	4.001163000
N	-1.791576000	0.199367000	2.704902000
C	-0.215112000	-0.443182000	-2.869373000
N	0.208377000	-1.703828000	-2.588237000
N	-0.882338000	-0.286720000	-4.050236000
C	-2.053802000	0.561941000	-4.165286000
H	-1.864846000	1.449768000	-4.775899000
H	-2.857120000	-0.005693000	-4.641863000
H	-2.384185000	0.865819000	-3.177162000
C	1.357214000	-1.950141000	-1.732237000
H	1.995636000	-1.071389000	-1.709144000
H	1.054580000	-2.212045000	-0.713752000
H	1.924091000	-2.789547000	-2.140308000
C	-0.523362000	-1.024727000	-5.251727000
H	-0.425003000	-0.325089000	-6.085735000
H	0.428536000	-1.527477000	-5.107717000
H	-1.281098000	-1.766428000	-5.518722000
C	-0.608195000	-2.879545000	-2.865737000
H	-0.101976000	-3.562593000	-3.551925000
H	-0.805886000	-3.400927000	-1.927120000
H	-1.560262000	-2.581453000	-3.293066000
C	-0.720322000	2.912117000	-2.196007000
H	-0.340933000	3.933937000	-2.225875000
H	-1.435659000	2.798805000	-3.004903000
H	-1.240533000	2.770578000	-1.247640000
C	1.116245000	2.014414000	-3.687372000
H	1.958079000	1.324978000	-3.763636000
H	0.416750000	1.795549000	-4.489325000
H	1.485627000	3.025758000	-3.850041000
C	1.840757000	1.572837000	3.548754000
H	1.087490000	2.356326000	3.641893000
H	1.705239000	0.864162000	4.361556000
H	2.823942000	2.024312000	3.673150000
C	2.855355000	-0.181910000	2.039718000
H	2.863303000	-0.875661000	2.873831000
H	2.719254000	-0.745641000	1.116390000
H	3.829663000	0.306239000	2.000613000
C	-0.827767000	-0.559609000	5.267025000
H	-1.412015000	0.355480000	5.226003000
H	-1.489009000	-1.392391000	5.522700000
H	-0.084271000	-0.462325000	6.062069000
C	0.793360000	-1.879654000	3.974475000
H	1.701034000	-1.666544000	4.545975000
H	0.315523000	-2.756148000	4.420152000
H	1.048558000	-2.116137000	2.946616000
C	-2.829766000	-0.771659000	3.029822000
H	-3.409497000	-0.982132000	2.129398000

H	-2.379404000	-1.702281000	3.358241000
H	-3.501688000	-0.395243000	3.804661000
C	-2.237047000	1.323606000	1.901328000
H	-1.454058000	2.074740000	1.848661000
H	-2.509010000	1.012056000	0.887688000

H	-3.122194000	1.761006000	2.367666000

References

1) Quek, S.; Debnath, S.; Laxmi, S.; Gastel, M.; Krämer, T.; England, J. Sterically Stabilized End-On Superoxocopper(II) Complexes and Mechanistic Insights Into Their Reactivity With O-H, N-H and C-H Substrates. *J. Am. Chem. Soc.* **2021**, *143*, 19731−19747.
2) Liang, H.-C.; Kim, E.; Incarvito, C. D.; Rheingold, A. L.; Karlin, K. D. A Bis-Acetonitrile Two-Coordinate Copper(I) Complex: Synthesis and Characterization of Highly Soluble $B(C_6F_5)_4^-$ Salts of $[Cu(MeCN)_2]^+$ and $[Cu(MeCN)_4]^+$. *Inorg. Chem.* **2002**, *41*, 2209-2212.
3) *SAINT and SADABS*, Bruker AXS Inc.: Madison, WI, 2007.
4) Sheldrick, G., A short history of SHELX. *Acta Crystallographica Section A* **2008,** *64*, 112-122.
5) Sheldrick, G., SHELXT - Integrated space-group and crystal-structure determination. *Acta Crystallographica Section A* **2015,** *71*, 3-8.
6) Finkelstein, K. D.; Pollock, C. J.; Lyndaker, A.; Krawcyk, T.; Conrad, J. Dual-Array Valence Emission Spectrometer (DAVES): A New Approach for Hard X-ray Photon-in Photon-out Spectroscopies. In *Proceedings of the 12th International Conference on Synchrotron Radiation Instrumentation,* New York, NY USA, July 6-10, 2015.; AIP Conference Proceedings, 2016; Vol. 1741, p 030009.
7) Solé, V. A.; Papillon, E.; Cotte, M.; Walter, Ph.; Susini, J. A Multiplatform Code for the Analysis of Energy-Dispersive X-Ray Fluorescence Spectra. *Spectrochim. Acta B* **2007**, *62*, 63-68.
8) Bordwell, F. G.; Zhang, X.-M. Acidities and homolytic bond dissociation enthalpies of 4-substituted-2,6-di-tert-butylphenols. *J. Phys. Org. Chem.* **1995**, *8*, 529-535.
9) Lee, J. Y.; Peterson, R. L.; Ohkubo, K.; Garcia-Bosch, I.; Himes, R. A.; Woertink, J.; Moore, C. D.; Solomon, E. I.; Fukuzumi, S.; Karlin, K. D. Mechanistic Insights into the Oxidation of Substituted Phenols via Hydrogen Atom Abstraction by a Cupric-Superoxo Complex. *J. Am. Chem. Soc.* **2014,** *136*, 9925-9937.
10) Zhu, X.-Q.; Zhang, M.-T.; Yu, A.; Wang, C.-H.; Cheng, J.-P. Hydride, Hydrogen Atom, Proton, and Electron Transfer Driving Forces of Various Five-Membered Heterocyclic Organic Hydrides and Their Reaction Intermediates in Acetonitrile. *J. Am. Chem. Soc.* **2008**, *130*, 2501-2516.
11) Warren, J. J.; Tronic, T. A.; Mayer, J. M. Thermochemistry of Proton-Coupled Electron Transfer Reagents and its Implications. *Chem. Rev.* **2010**,*110*, 6961-7001.

12) Neese, F. Software Update: The ORCA program system – Version 5.0. *Wiley Interdiscip. Rev.: Comput. Mol. Sci.*, **2022**, e1606.
13) Becke, A. D. Density-functional thermochemistry. III. The role of exact exchange. *J. Chem. Phys.* **1993**, *98*, 5648–5652.
14) Stephens, P. J.; Devlin, F. J.; Chabalowski, C. F.; Frisch, M. J. Ab initio calculation of vibrational absorption and circular dichroism spectra using density functional force fields. *J. Phys. Chem.* **1994**, *98*, 11623–11627.
15) Lee, C.; Yang, W.; Parr, R. G. Development of the Colle-Salvetti correlation-energy formula into a functional of the electron density. *Phys. Rev. B* **1988**, *37*, 785–789.
16) Vosko, S. H.; Wilk, L.; Nusair, M. Accurate spin-dependent electron liquid correlation energies for local spin density calculations: a critical analysis. *Can. J. Phys.* **1980**, *58*, 1200–1211.
17) Neese, F.; Wennmohs, F.; Hansen, A.; Becker, U. Efficient, approximate and parallel Hartree-Fock and hybrid DFT calculations. A 'chain-of-spheres' algorithm for the Hartree-Fock exchange. *Chem. Phys*. **2009**, *356*, 98–109.
18) Weigend, F.; Ahlrichs, R. Balanced basis sets of split valence, triple zeta valence and quadruple zeta valence quality for H to Rn: Design and assessment of accuracy. *Phys. Chem. Chem. Phys*. **2005**, *7*, 3297–3305.
19) Weigend, F. Accurate Coulomb-fitting basis sets for H to Rn. *Phys. Chem. Chem. Phys*. **2006**, *8*, 1057–1065.
20) Grimme, S.; Ehrlich, S.; Goerigk, L. Effect of the damping function in dispersion corrected density functional theory. *J. Comput. Chem.* **2011**, *32*, 1456–1465.
21) Grimme, S.; Antony, J.; Ehrlich, S.; Krieg, H. A consistent and accurate ab initio parametrization of density functional dispersion correction (DFT-D) for the 94 elements H-Pu. *J. Chem. Phys.* **2010**, *132*, 154104.
22) Noodleman, L. Valence bond description of antiferromagnetic coupling in transition metal dimers. *J. Chem. Phys.* **1981**, *74*, 5737–5743.
23) Yanai, Y.; Tew, D. P.; Handy, N. C. A new hybrid exchange-correlation functional using the Coulomb-attenuating method (CAM-B3LYP). *Chem. Phys. Lett*., **2004**, *393*, 51–57.
24) Balabanov, N. B.; Peterson, K. A. Systematically converged basis sets for transition metals. I. All-electron correlation consistent basis sets for the 3d elements Sc-Zn. *J. Chem. Phys*. **2005**, *123*, 064107.

25) Balabanov, N. B.; Peterson, K. A. Basis set limit electronic exchange energies, ionization potentials, and electron affinities for the 3d transition metal atoms: coupled cluster and multireference methods. *J. Chem. Phys*. **2006**, *125*, 074110.

26) Dunning, T. H. Gaussian basis sets for use in correlated molecular calculations. I. The atoms boron through neon and hydrogen. *J. Chem. Phys*. **1989**, *90*, 1007–1023.

27) Kendall, R. A.; Dunning, T. H.; Harrison R. J. Electron affinities of the first-row atoms revisited. Systematic basis sets and wave functions. *J. Chem. Phys*. **1992**, *96*, 6796–6806.

28) Liang, J.; Feng, X.; Hait, D.; Head-Gordon, M. Revisiting the Performance of Time-Dependent Density Functional Theory for Electronic Excitations: Assessment of 43 Popular and Recently Developed Functionals from Rungs One to Four. *J. Chem. Theory Comput*. **2022**, *18*, 3460−3473.

29) Cammi, R.; Mennucci, B.; Tomasi, J. Fast evaluation of geometries and properties of excited molecules in solution: a Tamm-Dancoff model with application to 4-dimethylaminobenzonitrile. *J. Phys. Chem. A* **2000**, *104*, 5631–5637.

30) Adamo, C.; Barone, V. Toward Reliable Density Functional Methods without Adjustable Parameters: The PBE0 Model. *J. Chem. Phys*. **1999**, *110*, 6158–6170.

31) Pantazis, D.A.; Chen, X.-Y.; Landis, C.R.; Neese, F. All-Electron Scalar Relativistic Basis Sets for Third-Row Transition Metal Atoms. *J. Chem. Theory Comput*. **2008**, *4*, 908–919.

32) Van Lenthe, E.; Baerends, E.J.; Snijders, J.G. Relativistic Regular Two-component Hamiltonians. *J. Chem. Phys.* **1993**, *99*, 4597–4610.

33) van Lenthe, E.; Baerends, E.J.; Snijders, J.G. Relativistic Total Energy Using Regular Approximations. *J. Chem. Phys.* **1994**, *101*, 9783–9792.

34) Heß, B.A.; Marian, C.M.; Wahlgren, U.; Gropen, O. A Mean-Field Spin-Orbit Method Applicable to Correlated Wavefunctions. *Chem. Phys. Lett.* **1996**, *251*, 365–371.

35) Neese, F. Efficient and Accurate Approximations to the Molecular Spin-Orbit Coupling Operator and Their Use in Molecular g-Tensor Calculations. *J. Chem. Phys.* **2005**, *122*, 034107

36) Neese, F. Calculation of the Zero-Field Splitting Tensor on the Basis of Hybrid Density Functional and Hartree-Fock Theory. *J. Chem. Phys.* **2007**, *127*, 164112.

37) George, S. D.; Petrenko, T.; Neese, F. Time-Dependent Density Functional Calculations of Ligand K-Edge X-Ray Absorption Spectra. *Inorganica Chimica Acta* **2008**, *361*, 965–972.

38) Siegbahn, P. E. M.; Heiberg, A.; Roos, B. O.; Levy, B. Comparison of the Super-CI and the Newton-Raphson Scheme in the Complete Active Space SCF Method, *Physica Scripta*, **1980**, *21*, 323–327.

39) Roos, B. O.; Taylor, P. R.; Siegbahn, P. E. M. A Complete Active Space SCF Method (CASSCF) Using a Density-Matrix Formulated Super-CI Approach, *Chem. Phys*., **1980**, *48*, 157–173.

40) Siegbahn, P. E. M.; Almlöf, J., Heiberg, A.; Roos, B. O. The Complete Active Space SCF (CASSCF) Method in a Newton-Raphson Formulation with Application to the HNO Molecule. *J. Chem. Phys*., **1981**, *74*, 2384–2396.

41) Angeli, C.; Cimiraglia, R.; Evangelisti, S.; Leininger, T.; Malrieu, J.-P. Introduction of n-electron valence states for multireference perturbation theory *J. Chem. Phys*., **2001**, *114*, 10252–10264.

42) Angeli, C.; Cimiraglia, R.; Malrieu, J.-P. n-electron valence state perturbation theory: A spinless formulation and an efficient implementation of the strongly contracted and of the partially contracted variants *J. Chem. Phys*., **2002**, *117*, 9138–9153.

43) Larsson, E. D.; Dong, G.; Veryazov, V.; Ryde, U.; Hedegård, E. D. Is density functional theory accurate for lytic polysaccharide monooxygenase enzymes? *Dalton Trans*. **2020**, *49*, 1501–1512.

44) Huber, S. M.; Shahi, A. R. M.; Aquilante, F.; Cramer, C. J.; Gagliardi, L. What Active Space Adequately Describes Oxygen Activation by a Late Transition Metal? CASPT2 and RASPT2 Applied to Intermediates from the Reaction of O_2 with a Cu(I)-α-Ketocarboxylate. *J. Chem. Theory Comput.* **2009***, 5,* 2967–2976.

45) Hess, B. A. Relativistic electronic-structure calculations employing a two-component no-pair formalism with external-field projection operators. *Phys. Rev. A* **1986**, *33*, 3742–3748.

46) Reiher, M.; Wolf, A. Exact Decoupling of the Dirac Hamiltonian. I. General Theory. *J. Chem. Phys*. **2004**, *121*, 2037–2047.

47) Reiher, M.; Wolf, A. Exact Decoupling of the Dirac Hamiltonian. II. The Generalized Douglas-Kroll-Hess Transformation up to Arbitrary Order. *J. Chem. Phys*., **2004**, *121*, 10945–10956.

48) Zhurko, G. A. Chemcraft - Graphical Program for Visualization of Quantum Chemistry Computations. Version 1.8, Ivanovo, 2005, https://www.chemcraftprog.com.

49) Falivene, L.; Credendino, R.; Poater, A.; Petta, A.; Serra, L.; Oliva, R.; Scarano, V.; Cavallo, L. SambVca 2. A Web Tool for Analyzing Catalytic Pockets with Topographic Steric Maps. *Organometallics* **2016**, *35*, 2286−2293.

50) Falivene, L.; Cao, Z.; Petta, A.; Serra, L.; Poater, A.; Oliva, R.; Scarano, V.; Cavallo, L. Towards the online computer-aided design of catalytic pockets. *Nat. Chem.* **2019**, *11*, 872−879.

51) Yang, L.; Powell, D. R.; Houser, R. P. Structural variation in copper(I) complexes with pyridylmethylamide ligands: structural analysis with a new four-coordinate geometry index, τ4. *Dalton Trans.* **2007,** 955-964.
